# Systematic Elucidation of the Potential Mechanism of Erzhi Pill against Drug-Induced Liver Injury via Network Pharmacology Approach

**DOI:** 10.1155/2020/6219432

**Published:** 2020-01-06

**Authors:** Shao-jie Huang, Fei Mu, Fei Li, Wen-jun Wang, Wei Zhang, Lu Lei, Yang Ma, Jing-wen Wang

**Affiliations:** Department of Pharmacy, Xijing Hospital, Fourth Military Medical University, Xi'an 710032, China

## Abstract

**Objective:**

The purpose of this work was to investigate the bioactive compounds, core genes, and pharmacological mechanisms and to provide a further research orientation of Erzhi pill (EZP) on drug-induced liver injury (DILI).

**Methods:**

At first, we collected information of bioactive compounds of EZP from Traditional Chinese Medicine Systems Pharmacology Database and Analysis Platform (TCMSP) and previous studies. And then, the targets related to bioactive compounds and DILI were obtained from 4 public databases. At last, Cytoscape was used to establish a visual network. Moreover, Gene Ontology (GO) and Kyoto Encyclopedia of Genes and Genomes (KEGG) pathway analyses and network analysis were performed to investigate potential mechanism of EZP against DILI.

**Results:**

A total of 23 bioactive compounds and 89 major proteins of EZP were screened out as potential players against DILI. Association for bioactive compounds, core targets, and related pathways was analyzed, implying that core targets related to these pathways are ALB, AKT1, MAPK1, EGFR, SRC, MAPK8, IGF1, CASP3, HSP90AA1, and MMP9, and potential mechanisms of EZP acting on DILI are closely related to negative regulation of apoptosis process, improvement of lipid metabolism, and positive regulation of liver regeneration process.

**Conclusion:**

This study demonstrated the multicompound, multitarget, and multichannel characteristics of EZP, which provided a novel approach for further research the mechanism of EZP in the treatment of DILI.

## 1. Introduction

Drug-induced liver injury (DILI), which is defined as a liver injury due to medications, xenobiotics, and herbs that leads to liver dysfunction or abnormal liver serology, has been an important health concern around the world [1]. Crude annual incidence rate of DILI was 19.1 cases per 100000 inhabitants [2]. DILI can cause serious consequences. However, there are few drugs that have liver-protective effect. Therefore, safe and effective drugs against DILI are urgently needed. For treating DILI, traditional Chinese medicine (TCM) has unique advantages and has been proven to have liver-protective effects [3].

Erzhi pill (EZP), which is composed of *Ligustri lucidi fructus* (LLF) and *Ecliptae herba* (EH) on the ratio of 1 : 1, is a classic TCM formula and widely used to treat hepatic disease with a long history in China. The history of EZP treating hepatic disease can be traced back to Wu Minji's series “Fu shou Jing Fang” in the Ming Dynasty. In TCM theory, EZP can tonify liver and kidney, nourish Yin, and stop bleeding, [4] which is applied for collapse of liver and kidney Yin deficiency. In our previous study, we have found that the bioactive compound of EZP shows liver-protective effect via enhancing the antioxidative defense system, suppressing the inflammatory response and cell apoptosis of liver [5]. However, this study still focused on “single target and single pathway.” A holistic “multiple compounds, multiple targets, and multiple pathways” study is necessary to clarify how EZP produces liver-protective effect on DILI.

Network pharmacology, first proposed in 2007 [6], has become an effective tool to systematically analyze the mechanism of TCM formula with multiple compounds. Applications of network pharmacology to investigate mechanism of TCM have become an indispensable method for development of TCM [7]. In many previous studies, network pharmacology has successfully predicted potential targets and pathways of TCM [8–10]. Therefore, network pharmacology has been proved to be an effective method to explore potential targets and pathways of TCM by analyzing network of biological systems.

**Figure 1:**
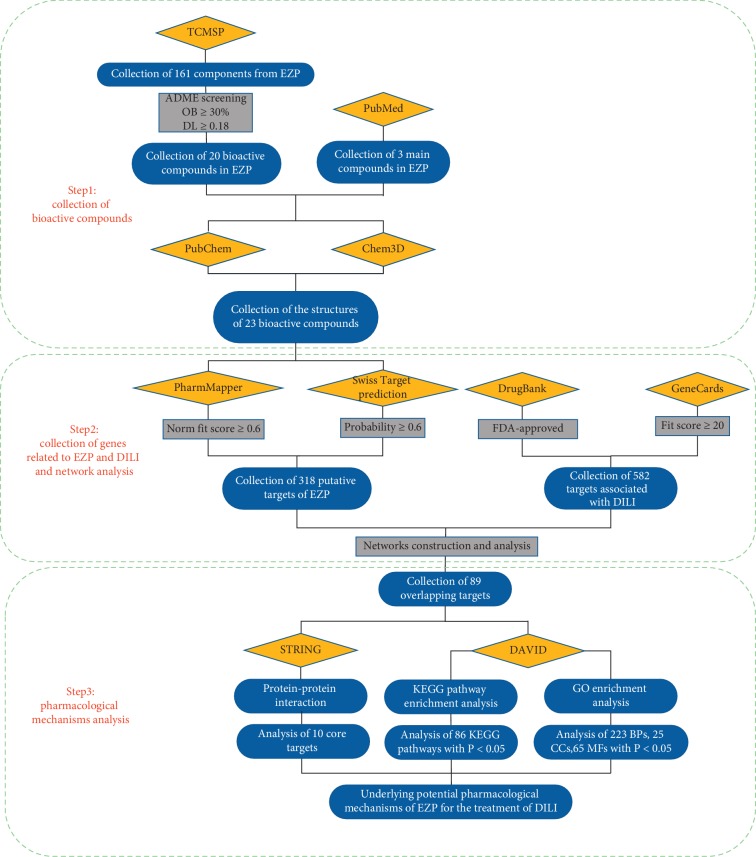
Workflow of network pharmacology analysis of EZP on DILI.

However, studies about the liver-protective effect of EZP on DILI are absenct. For the first time, this study explored the protective effect of EZP on DILI through network pharmacology and bioinformatic analysis. Workflow of this work is shown in detail in Figure 1.

## 2. Material and Methods

### 2.1. Collection of Bioactive Compounds of EZP

Information of compounds of EZP was collected from Traditional Chinese Medicine Systems Pharmacology Database (TCMSP, http://lsp.nwu.edu.cn/tcmsp.php, Version: 2.3), a website that can provide information of herbal ingredients and structures. In addition, TCMSP also provides absorption, distribution, metabolism, and excretion (ADME)-related parameters of herbal ingredients, such as oral bioavailability (OB), drug-likeness (DL), and half-life [11]. OB and DL were used to filter bioactive compounds of EZP after data were collected from TCMSP. OB, a major pharmacokinetic parameter of orally administered drugs, is used to measure the speed and extent of drug absorption into blood circulatory system [12]. DL is a qualitative principle to predict possibility of a chemical compound to become a drug, which can be applied to help optimize pharmacokinetics and pharmaceutical features in drug development [9]. Only compounds with OB ≥ 30% and DL ≥ 0.18 were identified for further study. As per this consideration, some compounds were removed by ADME screening, but these ingredients were identified as the main constituents of EZP in previous studies. So, we also identified these compounds as bioactive molecules.

### 2.2. Establishment of Bioactive Compounds Potential Targets Database

All the targets related to bioactive compounds of EZP were collected from PharmMapper (http://lilab-ecust.cn/pharmmapper/, Version 2017) and Swiss TargetPrediction (http://www.Swiss.Target.Prediction.ch/, 2019 version) by uploading the structure of bioactive compounds, which was acquired from The PubChem Compound Database (https://www.ncbi.nlm.nih.gov/pccompound) or drawn by Chem3D 16.0. PharmMapper and Swiss TargetPrediction are web servers for potential drug target prediction by reversed pharmacophore matching query compound against an in-house pharmacophore model database [13]. Only targets with a norm fit score (in PharmMapper) or Probability (in Swiss TargetPrediction) higher than 0.60 would be selected; the purpose of doing this is to ensure the reliability of prediction.

### 2.3. Construction of Target Database of DILI

The targets related to DILI were acquired form DrugBank (https://www.drugbank.ca/, version 5.1.4) and GeneCards (https://www.genecards.org/). These two databases illuminate relationship between targets and disease from different perspectives. DrugBank is a comprehensive, freely available database, from which the user can obtain information on detailed drug, drug target, drug action, and drug interaction of FDA-approved drugs or experimental drugs going through the FDA approval process [14]. GeneCards is also a comprehensive, freely available database, which provides information about targets related to disease, mutations and polymorphisms, gene expression, gene function, pathways, protein-protein interactions, and so on [15]. By searching the key word “drug-induced liver injury,” the targets related to DILI were collected. On the website of DrugBank, targets related to DILI were filtered by approved drug by the FDA. For keeping the reliability of the target prediction, we only chose the FDA-approved drugs in DrugBank and the targets with a norm fit score higher than 20 in GeneCards.

### 2.4. Network Establishment and Pathway Analyses

In order to investigate the possible mechanisms of EZP on DILI, common targets that related to DILI and putative targets of bioactive compounds were selected as EZP's targets against DILI. The nodes of network are bioactive compounds of LLF and EH networked with relevant disease targets [9]. All the targets were transferred to “ENTRY” by UniProt (https://www.uniprot.org/) before the establishment of network. The networks were established by Cytoscape 3.7.1, an open-source software project that is used for integrating biomolecular interaction networks with high-throughput expression data and other molecular states into a unified conceptual framework [16]. The pathways of EZP related to DILI were analyzed based on The Database for Annotation, Visualization and Integrated Discovery (DAVID, https://david.ncifcrf.gov/home.jsp, Vision 6.8), and KEGG (https://www.kegg.jp/, Release 91.0). The results of GO and KEGG pathway enrichment were considered to have statistically significant and necessary functional mechanisms of DILI, when *P* < 0.05.

### 2.5. Protein-Protein Interaction (PPI) Data Collection

Search Tool for the Retrieval of Interacting Genes (STRING, https://string-db.org/) was used to collect possible protein-protein interactions (PPI) by uploading 89 common targets that related to DILI and putative targets of active compounds. Species was limited to “*Homo sapiens*” with a confidence score >0.4.

## 3. Results

### 3.1. Bioactive Compounds' Screen of EZP

**Figure 2:**
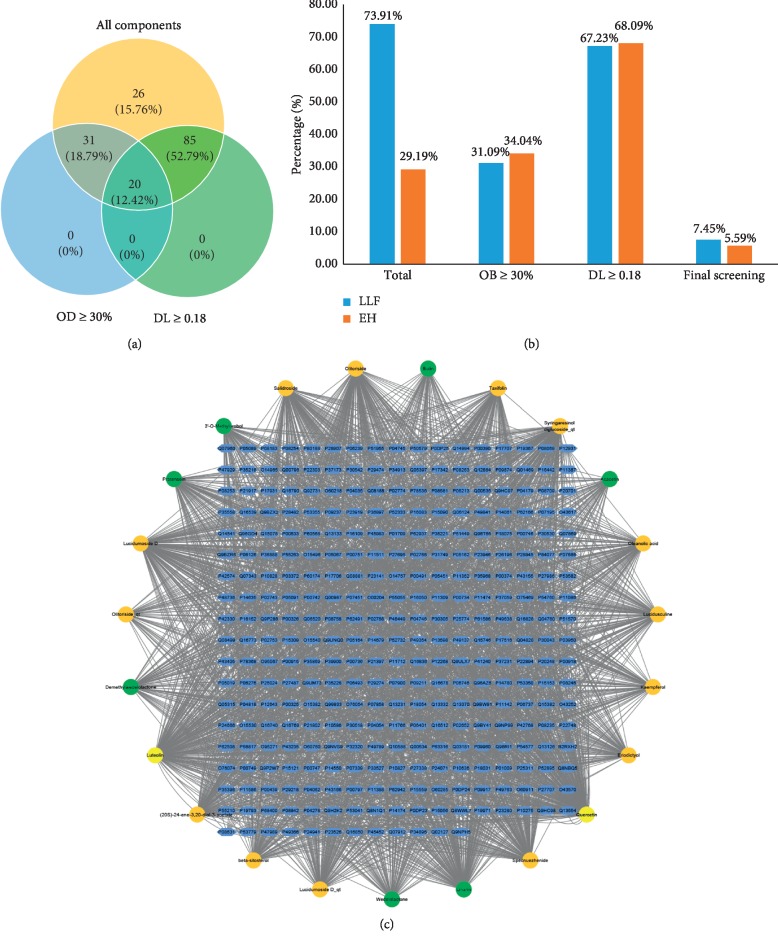
EZP compound-targets network. (a) Wayne figure: 166 compounds (yellow section), and 20 bioactive compounds screened by two ADME-related parameters (blue section stands for the compounds of OB ≥ 30%, green section stands for DL ≥ 0.18). (b) Distributions of different herbs. (c) Construction of EZP compound-target visual network, including 341 nodes and 2691 edges. Green nodes stand for bioactive compounds from EH, orange nodes stand for bioactive compounds from LLF, yellow nodes stand for duplicated compounds of EH and LLF, and blue nodes stand for putative targets.

A total of 166 compounds were collected from TCMSP: 119 in LLF and 47 in EH; among them, 5 compounds were duplicated and removed. Therefore, 161 compounds were identified from EZP. After ADME screening by OB ≥ 30% and DL ≥ 0.18, 20 compounds, 13 compounds from LLF and 9 compounds from EH with two repeated compounds (luteolin and quercetin), were identified as bioactive compounds of EZP (Figures 2(a) and 2(b)). Furthermore, some compounds (oleanolic acid, salidroside, and specnuezhenide) were removed by ADME screening, but these ingredients were identified as the main constituents of EZP in previous studies [17, 18]. At last, 23 compounds were identified as potential bioactive molecules for further study. The results of selected 23 compounds from LLF and EH are presented in Table 1.

### 3.2. EZP Putative Targets of EZP and Construction of Compounds-Targets Network

311 putative targets of LLF and 249 putative targets of EH were predicted by PharmMapper and Swiss TargetPrediction. After removing duplicated putative targets of LLF and EH, 318 putative targets linked to 23 compounds of EZP were collected. A visual EZP compounds-targets network with 341 nodes and 2691 edges was established by Cytoscape (Figure 2(c)). Quercetin, luteolin, linarin, lucidumoside D, and syringaresinol diglucoside_qt are top 5 bioactive compounds with maximum degree in network. These compounds are mainly flavonoids and their glycosides. Numerous studies have indicated that these compounds have liver-protective effect by regulating cell cycle or lipid metabolism [19–22]. Detailed information of putative targets was provided in Supplementary [Supplementary-material supplementary-material-1].

### 3.3. Target Database Establishment of DILI and Common-Target Network Analysis

**Figure 3:**
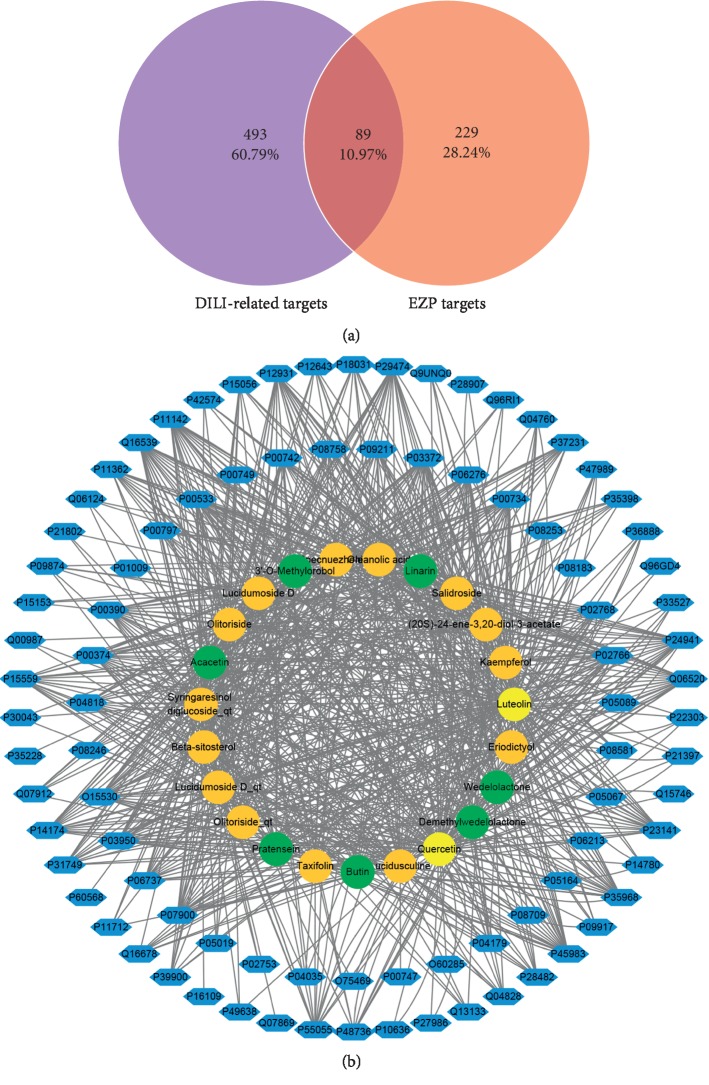
Common-target network. (a) 89 targets that are common to EZP and DILI. (b) Common-target network, including 112 nodes and 883 edges. Green nodes stand for bioactive compounds from EH, orange nodes stand for bioactive compounds from LLF, yellow nodes stand for duplicated compounds of EH and LLF, and blue nodes stand for putative targets.

At last, 582 targets related to DILI were obtained (267 targets from DrugBank and 357 targets from GeneCards with 42 targets duplicated). Detailed information on DILI-related targets is presented in Supplementary [Supplementary-material supplementary-material-1]. Based on previous study, 582 targets related to DILI and 318 putative targets of EZP, 89 common targets were selected (Figure 3(a)). Active compounds associated with selected overlapping targets are listed in Supplementary [Supplementary-material supplementary-material-1]. A visual EZP common-target network with 112 nodes (including 23 bioactive compounds and 89 targets) and 883 edges was established by Cytoscape (Figure 3(b)).

### 3.4. PPI Network of Common Targets

**Figure 4:**
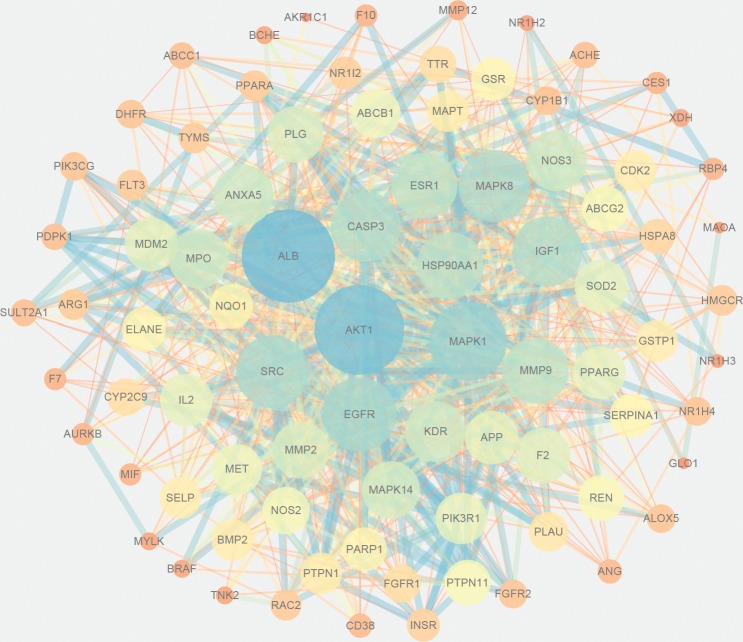
Protein-protein interaction (PPI) network of active compounds of EZP against DILI. Each node stands for a related target gene. The protein with greater degree is described by larger node and darker color, and the edge with greater combined score is described by thicker and darker line.

PPI network was obtained from STRING database by uploading 89 common targets. A combined score >0.4 and “*Homo sapiens*” was selected. And then, we established PPI network, which had 84 nodes and 811 edges by Cytoscape. In this network, the protein with greater degree was described by larger node and darker color, and the edge with greater combined score was described by thicker and darker line (Figure 4). 10 targets with highest degree score were select as core targets for DILI. The core targets, which may play an essential role against DILI, were serum albumin (ALB), RAC-alpha serine/threonine-protein kinase (AKT1), mitogen-activated protein kinase 1 (MAPK1), epidermal growth factor receptor (EGFR), insulin-like growth factor I (IGF1), caspase-3 (CASP3), proto-oncogene tyrosine-protein kinase Src (SRC), mitogen-activated protein kinase 8 (MAPK8), heat shock protein HSP 90-alpha (HSP90AA1), and matrix metalloproteinase-9 (MMP9).

### 3.5. GO and KEGG Pathway Enrichment Analysis

**Figure 5:**
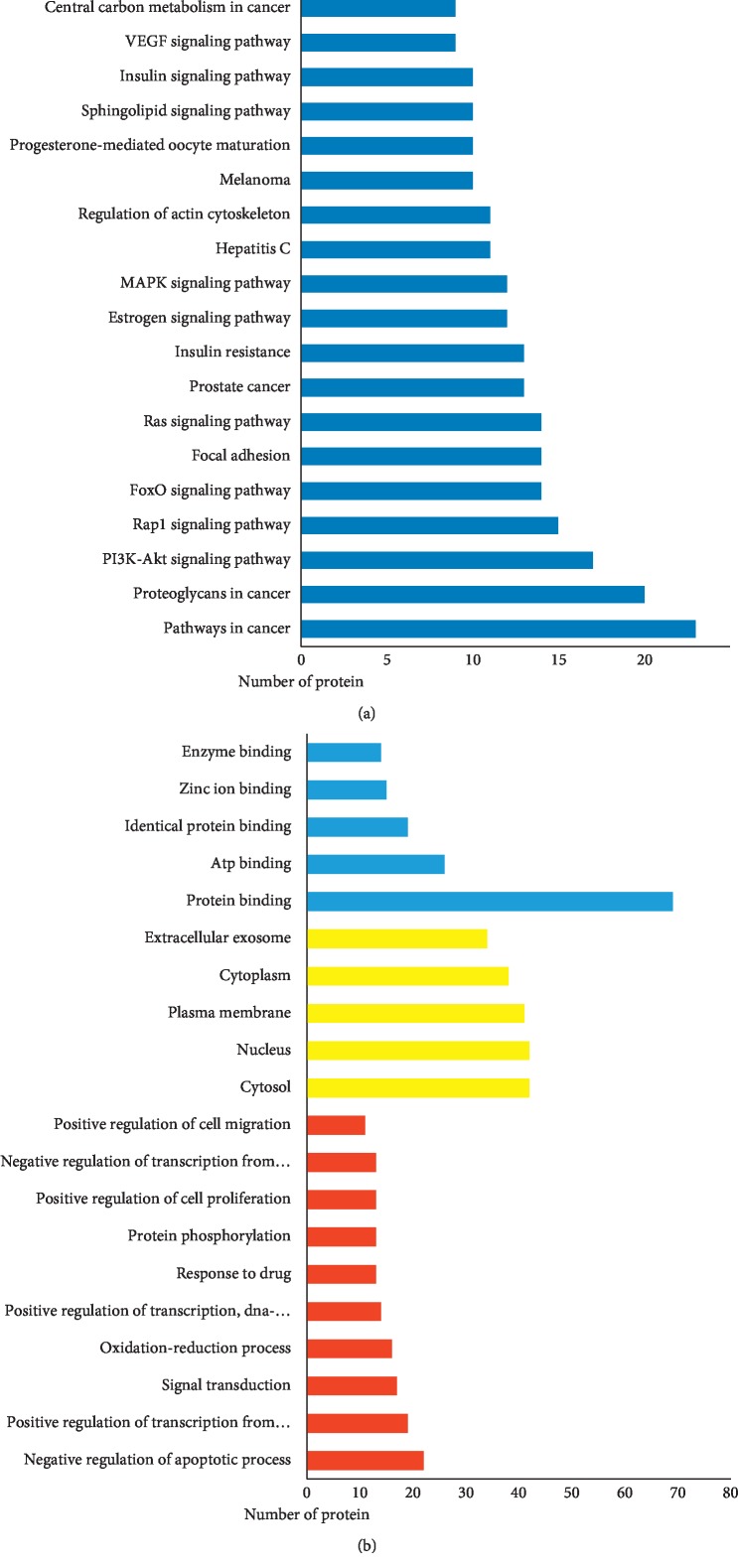
KEGG pathways and GO analysis. (a) KEGG pathway enrichment. (b) GO term analysis: red bars stand for BPs, yellow bars stand for CCs, and blue bars stand for MFs.

In order to explore possible mechanism of EZP against DILI, we analyzed GO term and KEGG pathway enrichment results executed by DAVID. GO term enrichment results were divided into biological process (BP), cell compound (CC), and molecular function (MF). Top 10 enriched conditions in BP and top 5 in CC and MF were shown in Figure 5(b). As shown in Supplementary [Supplementary-material supplementary-material-1], 223 BPs, 25 CCs, and 65 MFs enriched for these targets have a *Pvalue* less than 0.05. In GO term enrichment, the biological process of EZP against DILI may relate to negative regulation of apoptotic process, oxidation-reduction process, positive regulation of transcription from RNA polymerase II promoter, positive regulation of transcription, signal transduction, response to drug, and so on. Mainly, molecular functions are protein binging on 77.53% and ATP binging on 29.21%. Cell compound analysis showed that cytosol, nucleus, and plasma membrane accounted for the top 3 proportion (41, 41, and 38 targets, respectively). In addition, 86 KEGG pathways were recognized as *P* < 0.05. Top 20 KEGG pathways' enrichment analysis is shown in Figure 5(a) and Table 2. The results of KEGG enrichment analysis showed that the pathways of EZP against DILI mainly focus on multiple signaling pathways (including PI3K-Akt, FoxO, MAPK, sphingolipid, and VEGF signaling pathways), regulation of actin cytoskeleton, progesterone-mediated oocyte maturation, and so on. Interestingly, the results of pathways enrichment analysis can be divided into two function modules, including cell cycle regulation (such as MAPK, PI3K-Akt, and VEGF signaling pathways) and metabolic pathway (such as insulin signaling pathway, and central carbon metabolism in cancer).

## 4. Discussion

DILI, which carries a high mortality rate, [23] has been a major public concern impacting patients, doctors, drug researchers, and drug regulators [24]. TCM has its unique advantages to treat complex disease for “holistic treatment concept.” However, multicompound and multitarget characteristics of TCM also brought a lot of difficulties for Chinese medicine research and restrained the development of TCM. Fortunately, network pharmacology, which is especially suitable for multicompound and multitarget research, provides a prospective method to solve this problem. In this study, we predict and analyze the potential mechanisms of EZP from the perspective of systematic network pharmacology method. The results of GO and KEGG enrichment analysis indicated that mechanisms of EZP against DILI may be closely associated with negative regulation of apoptosis process, improvement of lipid metabolism, and positive regulation of liver regeneration process.

According to GO term enrichment results, negative regulation of apoptotic process was the biological process with most targets (22 targets) involved with *P* < 0.05. Necrosis and apoptosis of hepatocytes, cholangiocytes, and endothelial cells are typical features of DILI. IGF1, SRC, ALB, MMP9, CASP3, EGFR, AKT1, and MAKP8, which are involved in this biological process, are included in the top ten targets of the PPI network with highest degree. As we know, CASP3 is a key enzyme in the execution of apoptosis. Evidence has shown that there is a significant upregulation of CASP3 in DILI [25]. In addition, there are two MAPK proteins involved in core targets. MAPK1 (extensively known as extracellular signal‐regulated kinase 2, ERK2) takes party in multiple cellular processes such as cell proliferation, differentiation, adhesion, migration, and survival [26]. MAPK8 (known as c-Jun *N*-terminal kinase 1, JNK1) has diverse functions in cell cycle, such as cell death, regeneration, and differentiation [27]. These 3 genes (CASP3, MAPK1, and MAPK8) are all involved in MAPK signaling pathway (Table 2). These results indicated that negative regulation of apoptotic process and these proteins may play an essential role in EZP against DILI. In addition, KEGG enrichment analysis also showed that the mechanisms of EZP against DILI are closely related to PI3K-Akt, FoxO, MAPK, and VEGF signaling pathways. It is interesting to note that those pathways are all associated with cell cycle. In a previous study, it has been proved that the effect of EZP inhibition of hepatocyte apoptosis was closely associated with PI3K-Akt signaling pathway [28]. MAPK signaling pathway comprises the classic MAP kinase pathway, JNK and P38 MAP kinase pathway, and ERK5 pathway. Among them, JNK and P38 MAP kinase pathway is closely related to DILI. Drugs can be metabolized by P450s to reactive metabolites, which can activate JNK pathway to induce apoptosis through the recruitment of Bax [27].

By analyzing the results of KEGG pathway enrichment, we also found that insulin signaling pathway and insulin resistance have a significant result in KEGG pathway enrichment analysis as shown in Figure 5(a). These pathways are emerged as key players in glucose and lipid metabolism. Drug-induced steatohepatitis (DIS), which pathological feature is intracellular accumulation of lipids in hepatocytes, is another form of DILI [29]. The mechanisms of DIS can be aligned with the four aspects: increased fatty acid synthesis; decreased lipoprotein export; decreased fatty acid *β*-oxidation; and increased mobilization and uptake of fatty acids [29–31]. These pathways show that EZP may have potential for improving lipid metabolism function, which is beneficial to ameliorate DILI, by mediating the inhibitory action of insulin or insulin-like growth factor. In addition, these pathways also take part in cell metabolism, differentiation, oxidative stress, autophagy, and aging [32].

PPI network analysis, as well as GO and KEGG pathway analysis indicated that there were 1 core target and 2 pathways closely associated with liver regeneration, namely VEGF, VEGF signaling pathway, and PI3K-Akt signaling pathway. The liver is an organ with strong ability to regenerate. There are three phases, priming stage, proliferative phase, and termination phase, involved in the overall process of liver regeneration [33]. The VEGF, a core target of PPI network, belongs to the angiogenic factors that potently involves in endothelial cell proliferation and survival in liver regeneration following damage [34]. VEGF promotes proliferation of hepatocytes through reconstruction of liver sinusoids by proliferation of sinusoidal endothelial cells [35]. As described above, PI3K-Akt signaling pathway emerged as a key player in negative regulation of apoptosis. However, the regulation of liver regeneration is dual through PI3K-Akt signaling pathway. On the one hand, PI3K-Akt signaling pathway plays an essential role in liver regeneration, which has been testified [36]. On the other hand, PI3K inhibition can diminish the expression of IL‐6 and TNF‐*α*, which ultimately leads to attenuated regeneration [34]. Hence, EZP's effect on liver regeneration via PI3K-Akt signaling pathway is complex and needs further research.

In this work, we investigate the potential mechanism of EZP against DILI; however, network pharmacology is just a prediction. Whether EZP acts against DILI by regulating these pathways and proteins needs further experimental verification.

## 5. Conclusion

In summary, this study explored the protective effect of EZP on DILI through network pharmacology and bioinformatic analysis for the first time. 23 bioactive compounds of EZP and 89 targets associated with DILI were identified, and 10 core targets were identified by analyzing PPI network analysis. GO and KEGG pathway enrichment analysis indicates that the mechanisms of EZP against DILI may be related to negative regulation of apoptosis process, improvement of lipid metabolism, and positive regulation of liver regeneration process through PI3K-Akt, MAPK, Foxo, VEGF, and insulin signaling pathways, as well as insulin resistance.

## Figures and Tables

**Table 1:** A list of the final selected compounds from EZP for network analysis.

No.	Molecule name	Structure	OB (%)	DL	Herb
1	Luteolin	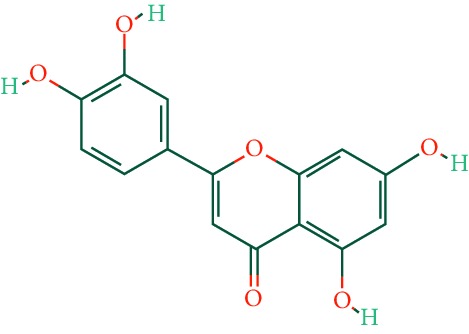	36.16	0.25	LLF, EH
2	Quercetin	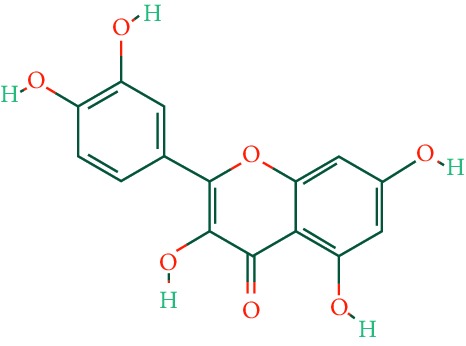	46.43	0.28	LLF, EH
3	Beta-sitosterol	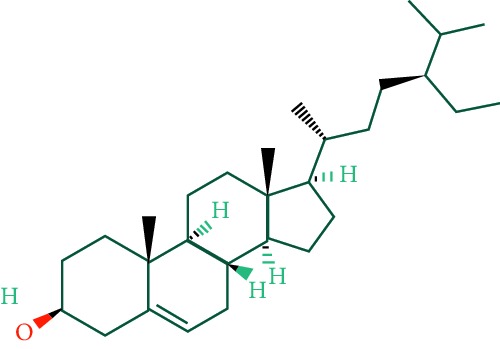	36.91	0.75	LLF
4	Kaempferol	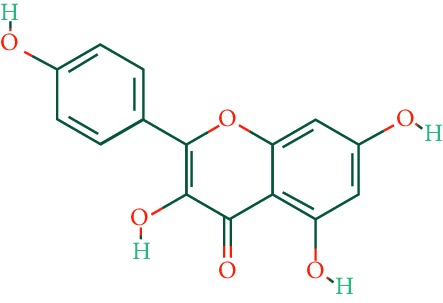	41.88	0.24	LLF
5	Acacetin	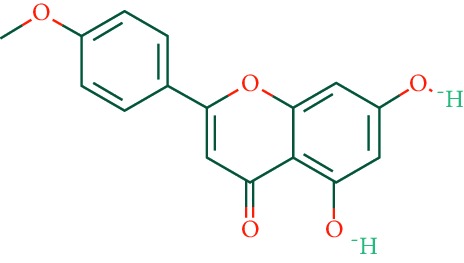	34.97	0.24	EH
6	Linarin	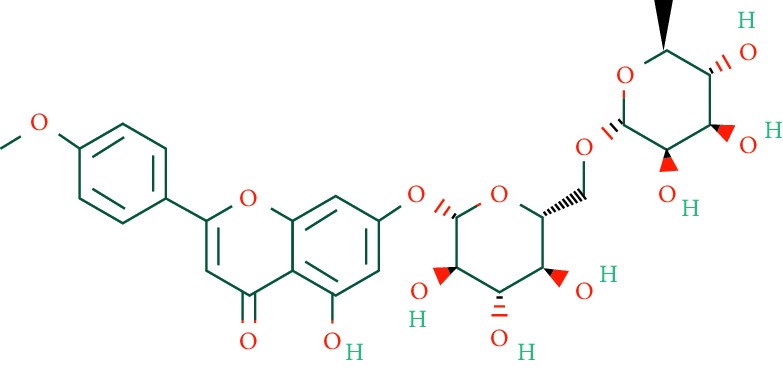	39.84	0.71	EH
7	Butin	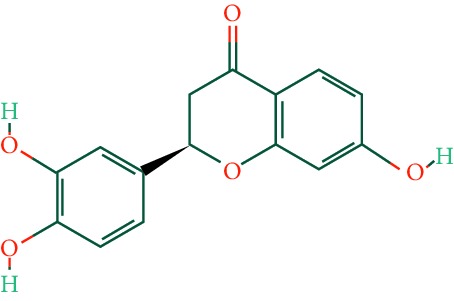	69.94	0.21	EH
8	3′-O-Methylorobol	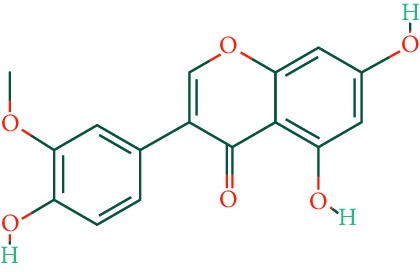	57.41	0.27	EH
9	Pratensein	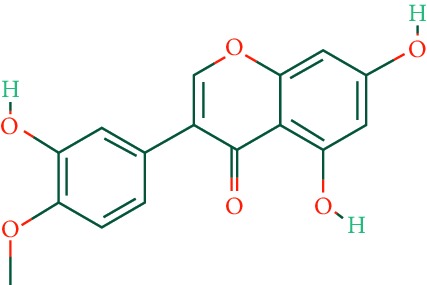	39.06	0.28	EH
10	Demethylwedelolactone	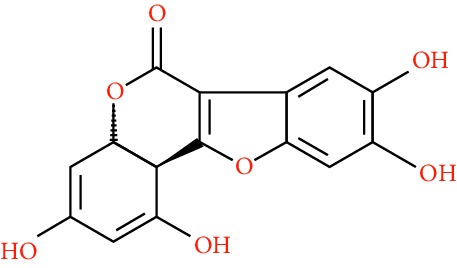	72.13	0.43	EH
11	Wedelolactone	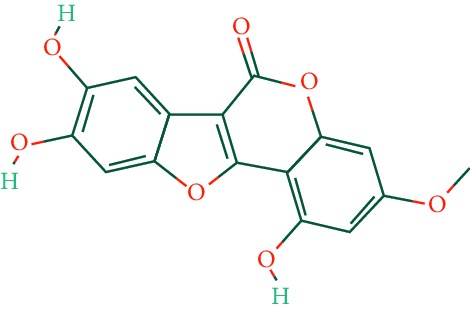	49.60	0.48	EH
12	Taxifolin	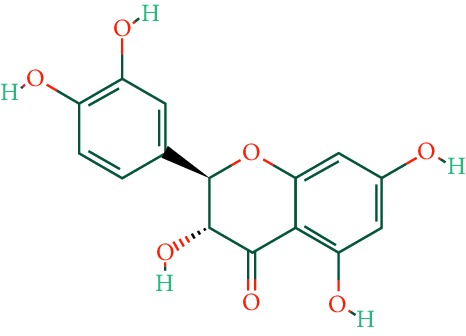	57.84	0.27	LLF
13	Lucidumoside D	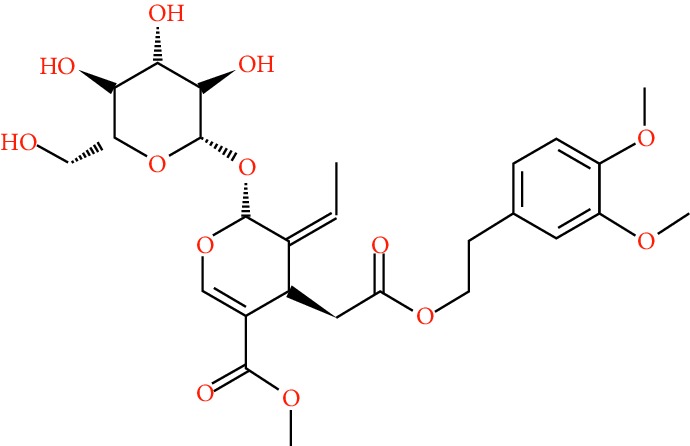	48.87	0.71	LLF
14	Lucidumoside D_qt	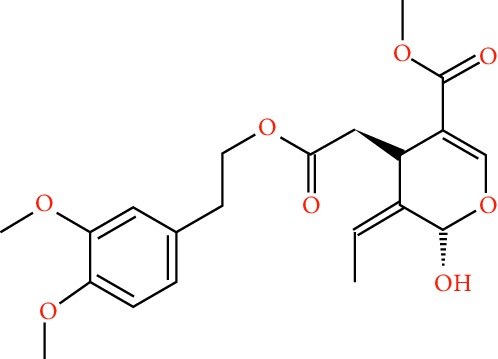	54.41	0.47	LLF
15	(20S)-24-ene-3*β*, 20-diol-3-acetate	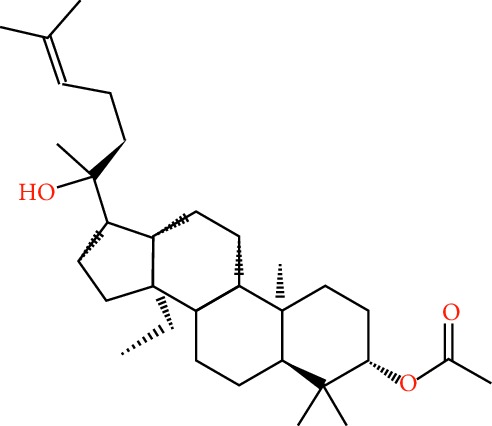	40.23	0.82	LLF
16	Eriodictyol	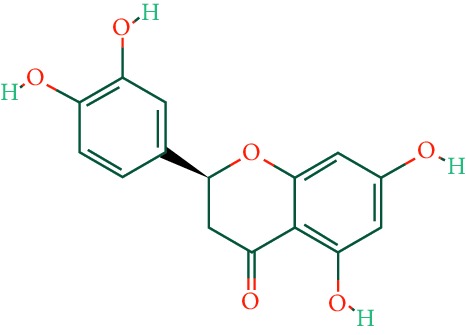	71.79	0.24	LLF
17	Syringaresinol diglucoside_qt	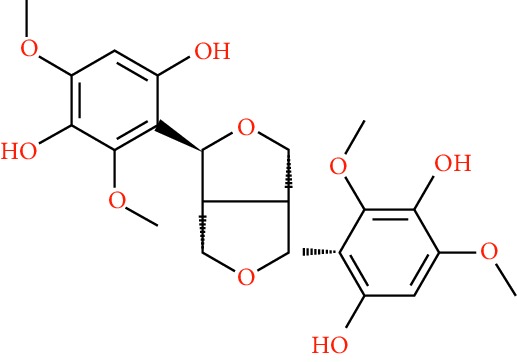	83.12	0.80	LLF
18	Lucidusculine	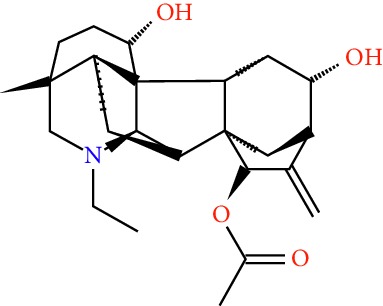	30.11	0.75	LLF
19	Olitoriside	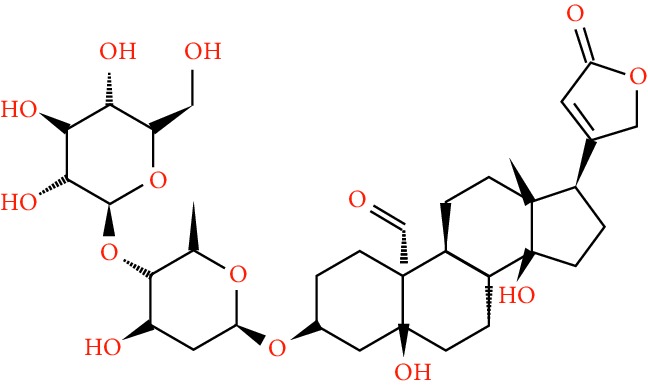	65.45	0.23	LLF
20	Olitoriside_qt	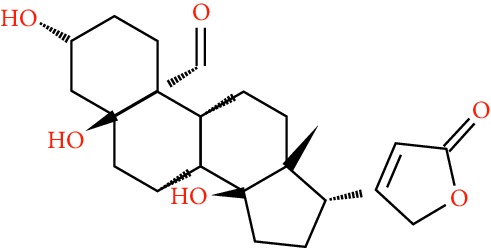	103.23	0.78	LLF
21	Oleanolic acid	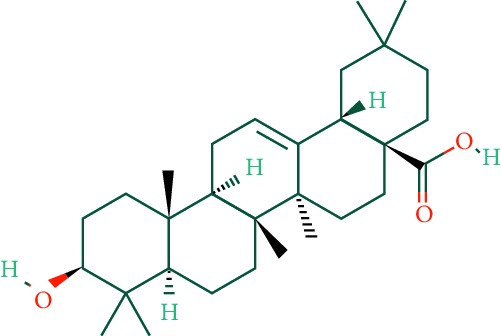	29.02	0.76	LLF
22	Salidroside	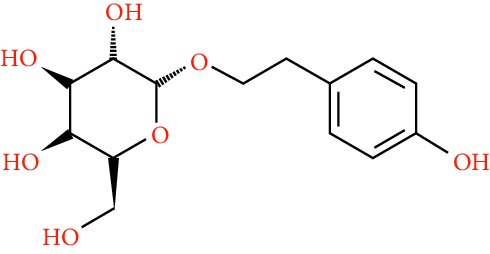	7.01	0.20	LLF
23	Specnuezhenide	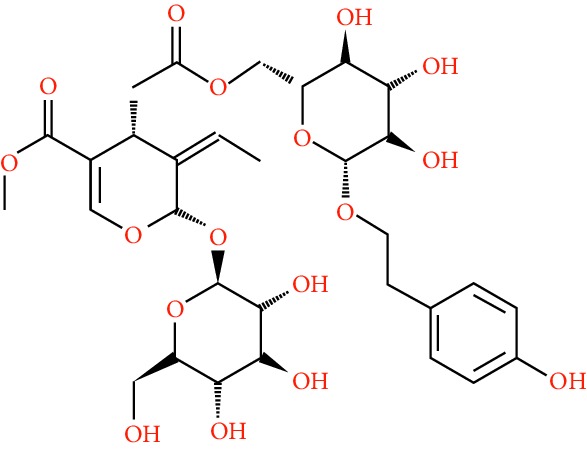	19.30	0.50	LLF

**Table 2:** Functions of potential target genes based on KEGG pathway analysis.

Term	Number of pathway gene	*P* value
Pathways in cancer	IGF1, FLT3, FGFR1, MET, PIK3CG, MMP9, NOS2, CDK2, HSP90AA1, PIK3R1, MDM2, RAC2, GSTP1, MMP2, MAPK1, FGFR2, CASP3, PPARG, EGFR, BMP2, AKT1, BRAF, MAPK8	1.05*E* – 10

Proteoglycans in cancer	IGF1, SRC, FGFR1, MET, PIK3CG, MMP9, ESR1, PIK3R1, KDR, PDPK1, MDM2, MMP2, PTPN11, MAPK1, CASP3, PLAU, MAPK14, EGFR, AKT1, BRAF	2.44*E* – 13

PI3K-Akt signaling pathway	IGF1, FGFR1, MET, PIK3CG, CDK2, IL2, HSP90AA1, PIK3R1, KDR, PDPK1, MDM2, NOS3, FGFR2, MAPK1, INSR, EGFR, AKT1	7.80*E* – 07

Rap1 signaling pathway	IGF1, SRC, FGFR1, MET, PIK3CG, PIK3R1, KDR, RAC2, FGFR2, MAPK1, INSR, MAPK14, EGFR, AKT1, BRAF,	5.12*E* – 08

FoxO signaling pathway	IGF1, PIK3CG, CDK2, PIK3R1, PDPK1, MDM2, SOD2, MAPK1, INSR, MAPK14, EGFR, AKT1, MAPK8, BRAF	1.74*E* – 09

Focal adhesion	IGF1, SRC, MET, PIK3CG, PIK3R1, KDR, MYLK, PDPK1, RAC2, MAPK1, EGFR, AKT1, MAPK8, BRAF	3.09*E* – 07

Ras signaling pathway	IGF1, PIK3R1, KDR, RAC2, FGFR1, PTPN11, MET, MAPK1, FGFR2, PIK3CG, INSR, EGFR, AKT1, MAPK8	8.97*E* – 07

Prostate cancer	IGF1, FGFR1, PIK3CG, CDK2, PIK3R1, HSP90AA1, PDPK1, MDM2, FGFR2, MAPK1, EGFR, AKT1, BRAF	1.42*E* – 10

Insulin resistance	NR1H3, PIK3CG, PYGL, PTPN1, PIK3R1, PDPK1, NOS3, PTPN11, INSR, PPARA, AKT1, NR1H2, MAPK8	1.62*E* – 09

Estrogen signaling pathway	HSP90AA1, PIK3R1, HSPA8, ESR1, SRC, NOS3, MMP2, MAPK1, PIK3CG, MMP9, EGFR, AKT1	8.25*E* – 09

MAPK signaling pathway	MAPT, HSPA8, RAC2, FGFR1, MAPK1, FGFR2, CASP3, MAPK14, EGFR, AKT1, BRAF, MAPK8	9.21*E* – 05

Hepatitis C	PIK3R1, NR1H3, PDPK1, MAPK1, PIK3CG, MAPK14, PPARA, EGFR, AKT1, BRAF, MAPK8	1.68*E* – 06

Regulation of actin cytoskeleton	PIK3R1, MYLK, SRC, RAC2, FGFR1, MAPK1, FGFR2, PIK3CG, EGFR, F2, BRAF	9.31*E* – 05

Melanoma	PIK3R1, IGF1, MDM2, FGFR1, MET, MAPK1, PIK3CG, EGFR, AKT1, BRAF	6.49*E* – 08

Progesterone-mediated oocyte maturation	HSP90AA1, PIK3R1, IGF1, MAPK1, PIK3CG, MAPK14, CDK2, AKT1, BRAF, MAPK8	3.88*E* – 07

Sphingolipid signaling pathway	PIK3R1, PDPK1, RAC2, ABCC1, NOS3, MAPK1, PIK3CG, MAPK14, AKT1, MAPK8	5.91*E* – 06

Insulin signaling pathway	PIK3R1, PDPK1, MAPK1, PIK3CG, INSR, PYGL, PTPN1, AKT1, BRAF, MAPK8	1.85*E* – 05

VEGF signaling pathway	PIK3R1, KDR, SRC, RAC2, NOS3, MAPK1, PIK3CG, MAPK14, AKT1	2.81*E* – 07

Central carbon metabolism in cancer	PIK3R1, FLT3, FGFR1, MET, MAPK1, FGFR2, PIK3CG, EGFR, AKT1	4.11*E* – 07

HIF-1 signaling pathway	PIK3R1, IGF1, NOS3, MAPK1, PIK3CG, INSR, NOS2, EGFR, AKT1	9.27*E* – 06

## Data Availability

The data used to support the findings of this study are included within the article and the supplementary information file(s).

## Abbreviations

DILI: Drug-induced liver injury
EZP: Erzhi pill
TCMSP: Traditional Chinese medicine systems pharmacology database
ADME: Absorption: distribution: metabolism: and excretion
GO: Gene ontology
KEGG: Kyoto encyclopedia of genes and genomes
David: The database for annotation: visualization and integrated discovery
TCM: Traditional Chinese medicine
LLF: *Ligustri lucidi fructus*
EH: *Ecliptae herba*
OB: Oral bioavailability
DL: Drug-likeness
PPI: Protein-protein interaction
ALB: Serum albumin
AKT1: RAC-alpha serine/threonine-protein kinase
MAPK1: Mitogen-activated protein kinase 1
EGFR: Epidermal growth factor receptor
SRC: Proto-oncogene tyrosine-protein kinase Src
MAPK8: Mitogen-activated protein kinase 8
IGF1: Insulin-like growth factor I
CASP3: Caspase-3
HSP90AA1: Heat shock protein HSP 90-alpha
MMP9: Matrix metalloproteinase-9
BP: Biological process
CC: Cell compound
MF: Molecular function
ERK2: Extracellular signal‐regulated kinase 2
JNK: c-Jun *N*-terminal kinase 1
DIS: Drug-induced steatohepatitis.

## Supplementary Materials

https://www.uniprot.org/ Supplementary Materials Table S1: putative targets for chemicals contained in EZP. Chemname was obtained from TCMSP. Entry, Entry name, and Protein name were obtained from PharmMapper. Entry was converted to Gene name by Uniprot. (https://www.uniprot.org/) Table S2: targets related to DILI. Detailed information of targets related to DILI, there were 267 targets from DrugBank and 357 targets from GeneCards with 42 targets duplicated. Table S3: common targets related to bioactive compounds. Detailed information of 89 common targets related to 23 bioactive compounds. Table S4: the results of GO and KEGG pathway enrichment. 223 BPs, 25 CCs, 65 MFs enriched have a *P* value less than 0.05, and 86 KEGG pathways were recognized as *P* < 0.05.
