# Supplementary material for: Systematic Elucidation of the Potential Mechanism of Erzhi Pill against Drug-Induced Liver Injury via Network Pharmacology Approach
Source: Evid Based Complement Alternat Med. 2020 Jan 6;2020:6219432. doi: 10.1155/2020/6219432 (PMC6970004; doi:10.1155/2020/6219432)
Supplement: https://www.uniprot.org/ Supplementary Materials — Table S1: putative targets for chemicals contained in EZP. Chemname was obtained from TCMSP. Entry, Entry name, and Protein name were obtained from PharmMapper. Entry was converted to Gene name by Uniprot. (https://www.uniprot.org/) Table S2: targets related to DILI. Detailed information of targets related to DILI, there were 267 targets from DrugBank and 357 targets from GeneCards with 42 targets duplicated. Table S3: common targets related to bioactive compounds. Detailed information of 89 common targets related to 23 bioactive compounds. Table S4: the results of GO and KEGG pathway enrichment. 223 BPs, 25 CCs, 65 MFs enriched have a P value less than 0.05, and 86 KEGG pathways were recognized as P < 0.05. [file 6219432.f1.zip › 6219432.f1/Supplementary Table S1 (1).pdf]

Title: Uncovering the pharmacological mechanism of Erzhi pill on drug-induced liver injury based on network pharmacology

Table S1 Putative targets for chemicals contained in EZP

Chemname	Entry	Entry name	Organism
beta-sitosterol	P52895	AK1C2_HUMAN	Homo sapiens (Human)
beta-sitosterol	P27338	AOFB_HUMAN	Homo sapiens (Human)
beta-sitosterol	P11309	PIM1_HUMAN	Homo sapiens (Human)
beta-sitosterol	P12643	BMP2_HUMAN	Homo sapiens (Human)
beta-sitosterol	P08842	STS_HUMAN	Homo sapiens (Human)
beta-sitosterol	P49137	MAPK2_HUMAN	Homo sapiens (Human)
beta-sitosterol	P02652	APOA2_HUMAN	Homo sapiens (Human)
beta-sitosterol	P55210	CASP7_HUMAN	Homo sapiens (Human)
beta-sitosterol	P10828	THB_HUMAN	Homo sapiens (Human)
beta-sitosterol	P00918	CAH2_HUMAN	Homo sapiens (Human)
beta-sitosterol	P02768	ALBU_HUMAN	Homo sapiens (Human)
beta-sitosterol	P28482	MK01_HUMAN	Homo sapiens (Human)
beta-sitosterol	P06401	PRGR_HUMAN	Homo sapiens (Human)
beta-sitosterol	P08235	MCR_HUMAN	Homo sapiens (Human)
beta-sitosterol	P14061	DHB1_HUMAN	Homo sapiens (Human)
beta-sitosterol	P45452	MMP13_HUMAN	Homo sapiens (Human)
beta-sitosterol	P52732	KIF11_HUMAN	Homo sapiens (Human)
beta-sitosterol	P35398	RORA_HUMAN	Homo sapiens (Human)
beta-sitosterol	O76054	S14L2_HUMAN	Homo sapiens (Human)
beta-sitosterol	P02774	VTDB_HUMAN	Homo sapiens (Human)
beta-sitosterol	P03372	ESR1_HUMAN	Homo sapiens (Human)
beta-sitosterol	Q06520	ST2A1_HUMAN	Homo sapiens (Human)
beta-sitosterol	P20701	ITAL_HUMAN	Homo sapiens (Human)
beta-sitosterol	P78536	ADA17_HUMAN	Homo sapiens (Human)
beta-sitosterol	Q16539	MK14_HUMAN	Homo sapiens (Human)
beta-sitosterol	P45983	MK08_HUMAN	Homo sapiens (Human)
beta-sitosterol	P28845	DHI1_HUMAN	Homo sapiens (Human)
beta-sitosterol	P02766	TTHY_HUMAN	Homo sapiens (Human)
beta-sitosterol	P24941	CDK2_HUMAN	Homo sapiens (Human)
beta-sitosterol	P10275	ANDR_HUMAN	Homo sapiens (Human)
beta-sitosterol	P12931	SRC_HUMAN	Homo sapiens (Human)
beta-sitosterol	Q07343	PDE4B_HUMAN	Homo sapiens (Human)
beta-sitosterol	P00533	EGFR_HUMAN	Homo sapiens (Human)
beta-sitosterol	Q96RI1	NR1H4_HUMAN	Homo sapiens (Human)
beta-sitosterol	P0DP23	CALM1_HUMAN	Homo sapiens (Human)
beta-sitosterol	P0DP24	CALM2_HUMAN	Homo sapiens (Human)
beta-sitosterol	P0DP25	CALM3_HUMAN	Homo sapiens (Human)
beta-sitosterol	O15530	PDPK1_HUMAN	Homo sapiens (Human)
beta-sitosterol	P63316	TNNC1_HUMAN	Homo sapiens (Human)
beta-sitosterol	P00734	THRB_HUMAN	Homo sapiens (Human)
beta-sitosterol	P56817	BACE1_HUMAN	Homo sapiens (Human)
beta-sitosterol	Q06124	PTN11_HUMAN	Homo sapiens (Human)
beta-sitosterol	P11086	PNMT_HUMAN	Homo sapiens (Human)
beta-sitosterol	P53041	PPP5_HUMAN	Homo sapiens (Human)
beta-sitosterol	P36897	TGFR1_HUMAN	Homo sapiens (Human)
beta-sitosterol	P04278	SHBG_HUMAN	Homo sapiens (Human)
beta-sitosterol	P15056	BRAF_HUMAN	Homo sapiens (Human)
beta-sitosterol	P00915	CAH1_HUMAN	Homo sapiens (Human)
beta-sitosterol	P23141	EST1_HUMAN	Homo sapiens (Human)
beta-sitosterol	P15121	ALDR_HUMAN	Homo sapiens (Human)
beta-sitosterol	P08758	ANXA5_HUMAN	Homo sapiens (Human)
beta-sitosterol	P35968	VGFR2_HUMAN	Homo sapiens (Human)
beta-sitosterol	O14757	CHK1_HUMAN	Homo sapiens (Human)

beta-sitosterol	P37231	PPARG_HUMAN	Homo sapiens (Human)
beta-sitosterol	P27487	DPP4_HUMAN	Homo sapiens (Human)
beta-sitosterol	Q16828	DUS6_HUMAN	Homo sapiens (Human)
beta-sitosterol	O43617	TPPC3_HUMAN	Homo sapiens (Human)
beta-sitosterol	P49638	TTPA_HUMAN	Homo sapiens (Human)
beta-sitosterol	O60760	HPGDS_HUMAN	Homo sapiens (Human)
beta-sitosterol	P50579	MAP2_HUMAN	Homo sapiens (Human)
beta-sitosterol	P04818	TYSY_HUMAN	Homo sapiens (Human)
beta-sitosterol	Q02127	PYRD_HUMAN	Homo sapiens (Human)
beta-sitosterol	Q14994	NR1I3_HUMAN	Homo sapiens (Human)
beta-sitosterol	P08263	GSTA1_HUMAN	Homo sapiens (Human)
beta-sitosterol	P00742	FA10_HUMAN	Homo sapiens (Human)
beta-sitosterol	P06276	CHLE_HUMAN	Homo sapiens (Human)
beta-sitosterol	P55263	ADK_HUMAN	Homo sapiens (Human)
beta-sitosterol	P42768	WASP_HUMAN	Homo sapiens (Human)
beta-sitosterol	Q9BY41	HDAC8_HUMAN	Homo sapiens (Human)
beta-sitosterol	P08069	IGF1R_HUMAN	Homo sapiens (Human)
beta-sitosterol	Q13126	MTAP_HUMAN	Homo sapiens (Human)
beta-sitosterol	Q04828	AK1C1_HUMAN	Homo sapiens (Human)
beta-sitosterol	O00204	ST2B1_HUMAN	Homo sapiens (Human)
beta-sitosterol	P02753	RET4_HUMAN	Homo sapiens (Human)
beta-sitosterol	P29474	NOS3_HUMAN	Homo sapiens (Human)
beta-sitosterol	P43405	KSYK_HUMAN	Homo sapiens (Human)
beta-sitosterol	Q13133	NR1H3_HUMAN	Homo sapiens (Human)
beta-sitosterol	P19793	RXRA_HUMAN	Homo sapiens (Human)
beta-sitosterol	P54760	EPHB4_HUMAN	Homo sapiens (Human)
beta-sitosterol	P11474	ERR1_HUMAN	Homo sapiens (Human)
beta-sitosterol	P15559	NQO1_HUMAN	Homo sapiens (Human)
beta-sitosterol	P62508	ERR3_HUMAN	Homo sapiens (Human)
beta-sitosterol	P55055	NR1H2_HUMAN	Homo sapiens (Human)
beta-sitosterol	P00326	ADH1G_HUMAN	Homo sapiens (Human)
beta-sitosterol	P04035	HMDH_HUMAN	Homo sapiens (Human)
beta-sitosterol	Q16850	CP51A_HUMAN	Homo sapiens (Human)
kaempferol	Q9NPH5	NOX4_HUMAN	Homo sapiens (Human)
kaempferol	P15121	ALDR_HUMAN	Homo sapiens (Human)
kaempferol	P47989	XDH_HUMAN	Homo sapiens (Human)
kaempferol	P14679	TYRO_HUMAN	Homo sapiens (Human)
kaempferol	P36888	FLT3_HUMAN	Homo sapiens (Human)
kaempferol	P00918	CAH2_HUMAN	Homo sapiens (Human)
kaempferol	P09917	LOX5_HUMAN	Homo sapiens (Human)
kaempferol	P43166	CAH7_HUMAN	Homo sapiens (Human)
kaempferol	P37059	DHB2_HUMAN	Homo sapiens (Human)
kaempferol	P33527	MRP1_HUMAN	Homo sapiens (Human)
kaempferol	P14061	DHB1_HUMAN	Homo sapiens (Human)
kaempferol	P35869	AHR_HUMAN	Homo sapiens (Human)
kaempferol	O43570	CAH12_HUMAN	Homo sapiens (Human)
kaempferol	P11474	ERR1_HUMAN	Homo sapiens (Human)
kaempferol	P08183	MDR1_HUMAN	Homo sapiens (Human)
kaempferol	Q16678	CP1B1_HUMAN	Homo sapiens (Human)
kaempferol	Q9UNQ0	ABCG2_HUMAN	Homo sapiens (Human)
kaempferol	P30542	AA1R_HUMAN	Homo sapiens (Human)
kaempferol	P22748	CAH4_HUMAN	Homo sapiens (Human)
kaempferol	P22303	ACES_HUMAN	Homo sapiens (Human)
kaempferol	P21397	AOFA_HUMAN	Homo sapiens (Human)
kaempferol	Q04760	LGUL_HUMAN	Homo sapiens (Human)
kaempferol	P43405	KSYK_HUMAN	Homo sapiens (Human)
kaempferol	P49841	GSK3B_HUMAN	Homo sapiens (Human)
kaempferol	P14780	MMP9_HUMAN	Homo sapiens (Human)

kaempferol	P08253	MMP2_HUMAN	Homo sapiens (Human)
kaempferol	P16050	LOX15_HUMAN	Homo sapiens (Human)
kaempferol	P18054	LOX12_HUMAN	Homo sapiens (Human)
kaempferol	Q13332	PTPRS_HUMAN	Homo sapiens (Human)
kaempferol	P08631	HCK_HUMAN	Homo sapiens (Human)
kaempferol	P03372	ESR1_HUMAN	Homo sapiens (Human)
kaempferol	P06276	CHLE_HUMAN	Homo sapiens (Human)
kaempferol	P00734	THRB_HUMAN	Homo sapiens (Human)
kaempferol	P11309	PIM1_HUMAN	Homo sapiens (Human)
kaempferol	Q00534	CDK6_HUMAN	Homo sapiens (Human)
kaempferol	P62937	PPIA_HUMAN	Homo sapiens (Human)
kaempferol	P00533	EGFR_HUMAN	Homo sapiens (Human)
kaempferol	P04062	GLCM_HUMAN	Homo sapiens (Human)
kaempferol	P24941	CDK2_HUMAN	Homo sapiens (Human)
kaempferol	P02766	TTHY_HUMAN	Homo sapiens (Human)
kaempferol	Q9NP99	TREM1_HUMAN	Homo sapiens (Human)
kaempferol	P00751	CFAB_HUMAN	Homo sapiens (Human)
kaempferol	P00390	GSHR_HUMAN	Homo sapiens (Human)
kaempferol	P00915	CAH1_HUMAN	Homo sapiens (Human)
kaempferol	P55055	NR1H2_HUMAN	Homo sapiens (Human)
kaempferol	P53779	MK10_HUMAN	Homo sapiens (Human)
kaempferol	Q15078	CD5R1_HUMAN	Homo sapiens (Human)
kaempferol	P03950	ANGI_HUMAN	Homo sapiens (Human)
kaempferol	P10275	ANDR_HUMAN	Homo sapiens (Human)
kaempferol	O15382	BCAT2_HUMAN	Homo sapiens (Human)
kaempferol	P11142	HSP7C_HUMAN	Homo sapiens (Human)
kaempferol	P12931	SRC_HUMAN	Homo sapiens (Human)
kaempferol	P06401	PRGR_HUMAN	Homo sapiens (Human)
kaempferol	P27338	AOFB_HUMAN	Homo sapiens (Human)
kaempferol	P45983	MK08_HUMAN	Homo sapiens (Human)
kaempferol	O14965	AURKA_HUMAN	Homo sapiens (Human)
kaempferol	O15530	PDPK1_HUMAN	Homo sapiens (Human)
kaempferol	P15559	NQO1_HUMAN	Homo sapiens (Human)
kaempferol	P00736	C1R_HUMAN	Homo sapiens (Human)
kaempferol	Q13126	MTAP_HUMAN	Homo sapiens (Human)
kaempferol	Q07343	PDE4B_HUMAN	Homo sapiens (Human)
kaempferol	P06737	PYGL_HUMAN	Homo sapiens (Human)
kaempferol	P07858	CATB_HUMAN	Homo sapiens (Human)
kaempferol	O14757	CHK1_HUMAN	Homo sapiens (Human)
kaempferol	P56817	BACE1_HUMAN	Homo sapiens (Human)
kaempferol	P00491	PNPH_HUMAN	Homo sapiens (Human)
kaempferol	Q08499	PDE4D_HUMAN	Homo sapiens (Human)
kaempferol	P27707	DCK_HUMAN	Homo sapiens (Human)
kaempferol	O15496	PA2GX_HUMAN	Homo sapiens (Human)
kaempferol	P07900	HS90A_HUMAN	Homo sapiens (Human)
kaempferol	P00374	DYR_HUMAN	Homo sapiens (Human)
kaempferol	P09960	LKHA4_HUMAN	Homo sapiens (Human)
kaempferol	P20248	CCNA2_HUMAN	Homo sapiens (Human)
kaempferol	P45452	MMP13_HUMAN	Homo sapiens (Human)
kaempferol	P55210	CASP7_HUMAN	Homo sapiens (Human)
kaempferol	P04278	SHBG_HUMAN	Homo sapiens (Human)
kaempferol	P08254	MMP3_HUMAN	Homo sapiens (Human)
kaempferol	P35558	PCKGC_HUMAN	Homo sapiens (Human)
taxifolin	P43166	CAH7_HUMAN	Homo sapiens (Human)
taxifolin	O43570	CAH12_HUMAN	Homo sapiens (Human)
taxifolin	P22748	CAH4_HUMAN	Homo sapiens (Human)
taxifolin	P39900	MMP12_HUMAN	Homo sapiens (Human)
taxifolin	P11309	PIM1_HUMAN	Homo sapiens (Human)

taxifolin	P00918	CAH2_HUMAN	Homo sapiens (Human)
taxifolin	P11511	CP19A_HUMAN	Homo sapiens (Human)
taxifolin	P00734	THRB_HUMAN	Homo sapiens (Human)
taxifolin	P10275	ANDR_HUMAN	Homo sapiens (Human)
taxifolin	P08631	HCK_HUMAN	Homo sapiens (Human)
taxifolin	P00915	CAH1_HUMAN	Homo sapiens (Human)
taxifolin	P06276	CHLE_HUMAN	Homo sapiens (Human)
taxifolin	P00751	CFAB_HUMAN	Homo sapiens (Human)
taxifolin	P03372	ESR1_HUMAN	Homo sapiens (Human)
taxifolin	P00491	PNPH_HUMAN	Homo sapiens (Human)
taxifolin	P24941	CDK2_HUMAN	Homo sapiens (Human)
taxifolin	P62937	PPIA_HUMAN	Homo sapiens (Human)
taxifolin	P02766	TTHY_HUMAN	Homo sapiens (Human)
taxifolin	P06401	PRGR_HUMAN	Homo sapiens (Human)
taxifolin	P53779	MK10_HUMAN	Homo sapiens (Human)
taxifolin	P09211	GSTP1_HUMAN	Homo sapiens (Human)
taxifolin	P00390	GSHR_HUMAN	Homo sapiens (Human)
taxifolin	P45983	MK08_HUMAN	Homo sapiens (Human)
taxifolin	P00533	EGFR_HUMAN	Homo sapiens (Human)
taxifolin	Q12884	SEPR_HUMAN	Homo sapiens (Human)
taxifolin	Q00534	CDK6_HUMAN	Homo sapiens (Human)
taxifolin	P48736	PK3CG_HUMAN	Homo sapiens (Human)
taxifolin	Q16740	CLPP_HUMAN	Homo sapiens (Human)
taxifolin	P08842	STS_HUMAN	Homo sapiens (Human)
taxifolin	P02652	APOA2_HUMAN	Homo sapiens (Human)
taxifolin	P56817	BACE1_HUMAN	Homo sapiens (Human)
taxifolin	P02768	ALBU_HUMAN	Homo sapiens (Human)
taxifolin	P04062	GLCM_HUMAN	Homo sapiens (Human)
taxifolin	P25774	CATS_HUMAN	Homo sapiens (Human)
taxifolin	Q9NP99	TREM1_HUMAN	Homo sapiens (Human)
taxifolin	P08254	MMP3_HUMAN	Homo sapiens (Human)
taxifolin	P35968	VGFR2_HUMAN	Homo sapiens (Human)
taxifolin	Q00796	DHSO_HUMAN	Homo sapiens (Human)
taxifolin	P20248	CCNA2_HUMAN	Homo sapiens (Human)
taxifolin	Q15078	CD5R1_HUMAN	Homo sapiens (Human)
taxifolin	P00749	UROK_HUMAN	Homo sapiens (Human)
taxifolin	O14757	CHK1_HUMAN	Homo sapiens (Human)
taxifolin	O15496	PA2GX_HUMAN	Homo sapiens (Human)
taxifolin	P54760	EPHB4_HUMAN	Homo sapiens (Human)
taxifolin	P04278	SHBG_HUMAN	Homo sapiens (Human)
taxifolin	P03950	ANGI_HUMAN	Homo sapiens (Human)
taxifolin	O14965	AURKA_HUMAN	Homo sapiens (Human)
taxifolin	P80188	NGAL_HUMAN	Homo sapiens (Human)
taxifolin	O15382	BCAT2_HUMAN	Homo sapiens (Human)
taxifolin	P29474	NOS3_HUMAN	Homo sapiens (Human)
taxifolin	P00439	PH4H_HUMAN	Homo sapiens (Human)
taxifolin	P62508	ERR3_HUMAN	Homo sapiens (Human)
taxifolin	Q08499	PDE4D_HUMAN	Homo sapiens (Human)
taxifolin	P11142	HSP7C_HUMAN	Homo sapiens (Human)
taxifolin	P12931	SRC_HUMAN	Homo sapiens (Human)
taxifolin	P27338	AOFB_HUMAN	Homo sapiens (Human)
taxifolin	P04745	AMY1_HUMAN	Homo sapiens (Human)
taxifolin	Q07343	PDE4B_HUMAN	Homo sapiens (Human)
taxifolin	P78536	ADA17_HUMAN	Homo sapiens (Human)
taxifolin	P78368	KC1G2_HUMAN	Homo sapiens (Human)
taxifolin	P08758	ANXA5_HUMAN	Homo sapiens (Human)
taxifolin	P16152	CBR1_HUMAN	Homo sapiens (Human)
taxifolin	P14061	DHB1_HUMAN	Homo sapiens (Human)

taxifolin	Q16828	DUS6_HUMAN	Homo sapiens (Human)
taxifolin	P19793	RXRA_HUMAN	Homo sapiens (Human)
taxifolin	P06737	PYGL_HUMAN	Homo sapiens (Human)
taxifolin	P17707	DCAM_HUMAN	Homo sapiens (Human)
taxifolin	P15559	NQO1_HUMAN	Homo sapiens (Human)
taxifolin	P17342	ANPRC_HUMAN	Homo sapiens (Human)
taxifolin	P47929	LEG7_HUMAN	Homo sapiens (Human)
taxifolin	P07900	HS90A_HUMAN	Homo sapiens (Human)
taxifolin	P55055	NR1H2_HUMAN	Homo sapiens (Human)
taxifolin	P15121	ALDR_HUMAN	Homo sapiens (Human)
taxifolin	P18075	BMP7_HUMAN	Homo sapiens (Human)
taxifolin	P19971	TYPH_HUMAN	Homo sapiens (Human)
taxifolin	P45452	MMP13_HUMAN	Homo sapiens (Human)
taxifolin	Q13126	MTAP_HUMAN	Homo sapiens (Human)
taxifolin	P11766	ADHX_HUMAN	Homo sapiens (Human)
taxifolin	Q16539	MK14_HUMAN	Homo sapiens (Human)
taxifolin	O15530	PDPK1_HUMAN	Homo sapiens (Human)
taxifolin	P55210	CASP7_HUMAN	Homo sapiens (Human)
taxifolin	P07858	CATB_HUMAN	Homo sapiens (Human)
taxifolin	P00374	DYR_HUMAN	Homo sapiens (Human)
taxifolin	Q15382	RHEB_HUMAN	Homo sapiens (Human)
taxifolin	Q06520	ST2A1_HUMAN	Homo sapiens (Human)
taxifolin	P27707	DCK_HUMAN	Homo sapiens (Human)
taxifolin	P02774	VTDB_HUMAN	Homo sapiens (Human)
taxifolin	P28845	DHI1_HUMAN	Homo sapiens (Human)
taxifolin	P23141	EST1_HUMAN	Homo sapiens (Human)
taxifolin	P23526	SAHH_HUMAN	Homo sapiens (Human)
taxifolin	P00746	CFAD_HUMAN	Homo sapiens (Human)
taxifolin	P01709	LV208_HUMAN	Homo sapiens (Human)
taxifolin	P52732	KIF11_HUMAN	Homo sapiens (Human)
taxifolin	P09960	LKHA4_HUMAN	Homo sapiens (Human)
taxifolin	P08235	MCR_HUMAN	Homo sapiens (Human)
taxifolin	P00742	FA10_HUMAN	Homo sapiens (Human)
Lucidumoside D	P45983	MK08_HUMAN	Homo sapiens (Human)
Lucidumoside D	P08631	HCK_HUMAN	Homo sapiens (Human)
Lucidumoside D	P53779	MK10_HUMAN	Homo sapiens (Human)
Lucidumoside D	P09211	GSTP1_HUMAN	Homo sapiens (Human)
Lucidumoside D	P00918	CAH2_HUMAN	Homo sapiens (Human)
Lucidumoside D	P07339	CATD_HUMAN	Homo sapiens (Human)
Lucidumoside D	P08254	MMP3_HUMAN	Homo sapiens (Human)
Lucidumoside D	P24941	CDK2_HUMAN	Homo sapiens (Human)
Lucidumoside D	P55055	NR1H2_HUMAN	Homo sapiens (Human)
Lucidumoside D	Q9BW91	NUDT9_HUMAN	Homo sapiens (Human)
Lucidumoside D	P62942	FKB1A_HUMAN	Homo sapiens (Human)
Lucidumoside D	P02766	TTHY_HUMAN	Homo sapiens (Human)
Lucidumoside D	P04062	GLCM_HUMAN	Homo sapiens (Human)
Lucidumoside D	O60911	CATL2_HUMAN	Homo sapiens (Human)
Lucidumoside D	P00751	CFAB_HUMAN	Homo sapiens (Human)
Lucidumoside D	P53582	MAP11_HUMAN	Homo sapiens (Human)
Lucidumoside D	P06276	CHLE_HUMAN	Homo sapiens (Human)
Lucidumoside D	P62937	PPIA_HUMAN	Homo sapiens (Human)
Lucidumoside D	P26196	DDX6_HUMAN	Homo sapiens (Human)
Lucidumoside D	Q9NP99	TREM1_HUMAN	Homo sapiens (Human)
Lucidumoside D	O14757	CHK1_HUMAN	Homo sapiens (Human)
Lucidumoside D	P37173	TGFR2_HUMAN	Homo sapiens (Human)
Lucidumoside D	Q12884	SEPR_HUMAN	Homo sapiens (Human)
Lucidumoside D	P00915	CAH1_HUMAN	Homo sapiens (Human)
Lucidumoside D	P00491	PNPH_HUMAN	Homo sapiens (Human)

Lucidumoside D	P00533	EGFR_HUMAN	Homo sapiens (Human)
Lucidumoside D	P20248	CCNA2_HUMAN	Homo sapiens (Human)
Lucidumoside D	P11309	PIM1_HUMAN	Homo sapiens (Human)
Lucidumoside D	Q13126	MTAP_HUMAN	Homo sapiens (Human)
Lucidumoside D	P23946	CMA1_HUMAN	Homo sapiens (Human)
Lucidumoside D	O14965	AURKA_HUMAN	Homo sapiens (Human)
Lucidumoside D	P15121	ALDR_HUMAN	Homo sapiens (Human)
Lucidumoside D	P25774	CATS_HUMAN	Homo sapiens (Human)
Lucidumoside D	P49137	MAPK2_HUMAN	Homo sapiens (Human)
Lucidumoside D	P37231	PPARG_HUMAN	Homo sapiens (Human)
Lucidumoside D	P04745	AMY1_HUMAN	Homo sapiens (Human)
Lucidumoside D	Q07343	PDE4B_HUMAN	Homo sapiens (Human)
Lucidumoside D	P00734	THRB_HUMAN	Homo sapiens (Human)
Lucidumoside D	P03372	ESR1_HUMAN	Homo sapiens (Human)
Lucidumoside D	O43570	CAH12_HUMAN	Homo sapiens (Human)
Lucidumoside D	P45452	MMP13_HUMAN	Homo sapiens (Human)
Lucidumoside D	P07858	CATB_HUMAN	Homo sapiens (Human)
Lucidumoside D	P52732	KIF11_HUMAN	Homo sapiens (Human)
Lucidumoside D	P27338	AOFB_HUMAN	Homo sapiens (Human)
Lucidumoside D	P28482	MK01_HUMAN	Homo sapiens (Human)
Lucidumoside D	Q15078	CD5R1_HUMAN	Homo sapiens (Human)
Lucidumoside D	P11142	HSP7C_HUMAN	Homo sapiens (Human)
Lucidumoside D	P08842	STS_HUMAN	Homo sapiens (Human)
Lucidumoside D	Q92731	ESR2_HUMAN	Homo sapiens (Human)
Lucidumoside D	P06401	PRGR_HUMAN	Homo sapiens (Human)
Lucidumoside D	P48736	PK3CG_HUMAN	Homo sapiens (Human)
Lucidumoside D	Q8NBQ5	DHB11_HUMAN	Homo sapiens (Human)
Lucidumoside D	P25311	ZA2G_HUMAN	Homo sapiens (Human)
Lucidumoside D	Q9P2W7	B3GA1_HUMAN	Homo sapiens (Human)
Lucidumoside D	P54760	EPHB4_HUMAN	Homo sapiens (Human)
Lucidumoside D	P36897	TGFR1_HUMAN	Homo sapiens (Human)
Lucidumoside D	P19367	HXK1_HUMAN	Homo sapiens (Human)
Lucidumoside D	Q05315	LEG10_HUMAN	Homo sapiens (Human)
Lucidumoside D	P56817	BACE1_HUMAN	Homo sapiens (Human)
Lucidumoside D	P08758	ANXA5_HUMAN	Homo sapiens (Human)
Lucidumoside D	Q00796	DHSO_HUMAN	Homo sapiens (Human)
Lucidumoside D	P02652	APOA2_HUMAN	Homo sapiens (Human)
Lucidumoside D	Q16539	MK14_HUMAN	Homo sapiens (Human)
Lucidumoside D	P51570	GALK1_HUMAN	Homo sapiens (Human)
Lucidumoside D	P02768	ALBU_HUMAN	Homo sapiens (Human)
Lucidumoside D	P16083	NQO2_HUMAN	Homo sapiens (Human)
Lucidumoside D	P35968	VGFR2_HUMAN	Homo sapiens (Human)
Lucidumoside D	P11511	CP19A_HUMAN	Homo sapiens (Human)
Lucidumoside D	Q08499	PDE4D_HUMAN	Homo sapiens (Human)
Lucidumoside D	Q02127	PYRD_HUMAN	Homo sapiens (Human)
Lucidumoside D	P12931	SRC_HUMAN	Homo sapiens (Human)
Lucidumoside D	P15056	BRAF_HUMAN	Homo sapiens (Human)
Lucidumoside D	P00374	DYR_HUMAN	Homo sapiens (Human)
Lucidumoside D	P02774	VTDB_HUMAN	Homo sapiens (Human)
Lucidumoside D	P00439	PH4H_HUMAN	Homo sapiens (Human)
Lucidumoside D	P03950	ANGI_HUMAN	Homo sapiens (Human)
Lucidumoside D	O15496	PA2GX_HUMAN	Homo sapiens (Human)
Lucidumoside D	Q9NVS9	PNPO_HUMAN	Homo sapiens (Human)
Lucidumoside D	P78368	KC1G2_HUMAN	Homo sapiens (Human)
Lucidumoside D	Q13231	CHIT1_HUMAN	Homo sapiens (Human)
Lucidumoside D	P29474	NOS3_HUMAN	Homo sapiens (Human)
Lucidumoside D	P48449	ERG7_HUMAN	Homo sapiens (Human)
Lucidumoside D	P00736	C1R_HUMAN	Homo sapiens (Human)

Lucidumoside D	P00746	CFAD_HUMAN	Homo sapiens (Human)
Lucidumoside D	P06737	PYGL_HUMAN	Homo sapiens (Human)
Lucidumoside D	P18031	PTN1_HUMAN	Homo sapiens (Human)
Lucidumoside D	P28845	DHI1_HUMAN	Homo sapiens (Human)
Lucidumoside D	P35398	RORA_HUMAN	Homo sapiens (Human)
Lucidumoside D	P02743	SAMP_HUMAN	Homo sapiens (Human)
Lucidumoside D	P53350	PLK1_HUMAN	Homo sapiens (Human)
Lucidumoside D	P01709	LV208_HUMAN	Homo sapiens (Human)
Lucidumoside D	P04278	SHBG_HUMAN	Homo sapiens (Human)
Lucidumoside D	P14174	MIF_HUMAN	Homo sapiens (Human)
Lucidumoside D	P17707	DCAM_HUMAN	Homo sapiens (Human)
Lucidumoside D	Q99933	BAG1_HUMAN	Homo sapiens (Human)
Lucidumoside D	P47929	LEG7_HUMAN	Homo sapiens (Human)
Lucidumoside D	P07900	HS90A_HUMAN	Homo sapiens (Human)
Lucidumoside D	P49354	FNTA_HUMAN	Homo sapiens (Human)
Lucidumoside D	P19793	RXRA_HUMAN	Homo sapiens (Human)
Lucidumoside D	P18075	BMP7_HUMAN	Homo sapiens (Human)
Lucidumoside D	P04818	TYSY_HUMAN	Homo sapiens (Human)
Lucidumoside D	P08069	IGF1R_HUMAN	Homo sapiens (Human)
Lucidumoside D	O15382	BCAT2_HUMAN	Homo sapiens (Human)
Lucidumoside D	P31749	AKT1_HUMAN	Homo sapiens (Human)
Lucidumoside D	P43235	CATK_HUMAN	Homo sapiens (Human)
Lucidumoside D	P39900	MMP12_HUMAN	Homo sapiens (Human)
Lucidumoside D	P00742	FA10_HUMAN	Homo sapiens (Human)
Lucidumoside D	P00390	GSHR_HUMAN	Homo sapiens (Human)
Lucidumoside D	P42330	AK1C3_HUMAN	Homo sapiens (Human)
Lucidumoside D	P23141	EST1_HUMAN	Homo sapiens (Human)
Lucidumoside D	Q96T66	NMNA3_HUMAN	Homo sapiens (Human)
Lucidumoside D	P05451	REG1A_HUMAN	Homo sapiens (Human)
Lucidumoside D	P19971	TYPH_HUMAN	Homo sapiens (Human)
Lucidumoside D	P11362	FGFR1_HUMAN	Homo sapiens (Human)
Lucidumoside D	Q13370	PDE3B_HUMAN	Homo sapiens (Human)
Lucidumoside D	P10275	ANDR_HUMAN	Homo sapiens (Human)
Lucidumoside D	P29218	IMPA1_HUMAN	Homo sapiens (Human)
Lucidumoside D	P09874	PARP1_HUMAN	Homo sapiens (Human)
Lucidumoside D	P00325	ADH1B_HUMAN	Homo sapiens (Human)
Lucidumoside D	O76074	PDE5A_HUMAN	Homo sapiens (Human)
Lucidumoside D	P04746	AMYP_HUMAN	Homo sapiens (Human)
Lucidumoside D	P15090	FABP4_HUMAN	Homo sapiens (Human)
Lucidumoside D	Q96AZ6	ISG20_HUMAN	Homo sapiens (Human)
Lucidumoside D	P55210	CASP7_HUMAN	Homo sapiens (Human)
Lucidumoside D	P23919	KTHY_HUMAN	Homo sapiens (Human)
Lucidumoside D	O15530	PDPK1_HUMAN	Homo sapiens (Human)
Lucidumoside D	P43155	CACP_HUMAN	Homo sapiens (Human)
Lucidumoside D	P06239	LCK_HUMAN	Homo sapiens (Human)
Lucidumoside D	P49789	FHIT_HUMAN	Homo sapiens (Human)
Lucidumoside D	P50579	MAP2_HUMAN	Homo sapiens (Human)
Lucidumoside D	P11712	CP2C9_HUMAN	Homo sapiens (Human)
Lucidumoside D	P84077	ARF1_HUMAN	Homo sapiens (Human)
Lucidumoside D	Q03181	PPARD_HUMAN	Homo sapiens (Human)
Lucidumoside D	P78536	ADA17_HUMAN	Homo sapiens (Human)
Lucidumoside D	P00797	RENI_HUMAN	Homo sapiens (Human)
Lucidumoside D	P08581	MET_HUMAN	Homo sapiens (Human)
Lucidumoside D	P16152	CBR1_HUMAN	Homo sapiens (Human)
Lucidumoside D	Q15382	RHEB_HUMAN	Homo sapiens (Human)
Lucidumoside D	Q06520	ST2A1_HUMAN	Homo sapiens (Human)
Lucidumoside D	P23526	SAHH_HUMAN	Homo sapiens (Human)
Lucidumoside D	P35558	PCKGC_HUMAN	Homo sapiens (Human)

Lucidumoside D	P15559	NQO1_HUMAN	Homo sapiens (Human)
Lucidumoside D	P61586	RHOA_HUMAN	Homo sapiens (Human)
Lucidumoside D	P11586	C1TC_HUMAN	Homo sapiens (Human)
Lucidumoside D	P16442	BGAT_HUMAN	Homo sapiens (Human)
Lucidumoside D	O43252	PAPS1_HUMAN	Homo sapiens (Human)
Lucidumoside D	P27487	DPP4_HUMAN	Homo sapiens (Human)
Lucidumoside D	P22894	MMP8_HUMAN	Homo sapiens (Human)
Lucidumoside D	P52333	JAK3_HUMAN	Homo sapiens (Human)
Lucidumoside D	Q08881	ITK_HUMAN	Homo sapiens (Human)
Lucidumoside D	P08235	MCR_HUMAN	Homo sapiens (Human)
Lucidumoside D	Q07912	ACK1_HUMAN	Homo sapiens (Human)
Lucidumoside D	P34896	GLYC_HUMAN	Homo sapiens (Human)
Lucidumoside D	Q9BY41	HDAC8_HUMAN	Homo sapiens (Human)
Lucidumoside D	P15153	RAC2_HUMAN	Homo sapiens (Human)
Lucidumoside D	P05019	IGF1_HUMAN	Homo sapiens (Human)
Lucidumoside D	P01009	A1AT_HUMAN	Homo sapiens (Human)
Lucidumoside D	P04035	HMDH_HUMAN	Homo sapiens (Human)
Lucidumoside D	P49841	GSK3B_HUMAN	Homo sapiens (Human)
Lucidumoside D	Q10588	BST1_HUMAN	Homo sapiens (Human)
Lucidumoside D	P05091	ALDH2_HUMAN	Homo sapiens (Human)
eriodictyol	P43166	CAH7_HUMAN	Homo sapiens (Human)
eriodictyol	O43570	CAH12_HUMAN	Homo sapiens (Human)
eriodictyol	P22748	CAH4_HUMAN	Homo sapiens (Human)
eriodictyol	Q16678	CP1B1_HUMAN	Homo sapiens (Human)
eriodictyol	P11511	CP19A_HUMAN	Homo sapiens (Human)
eriodictyol	P10275	ANDR_HUMAN	Homo sapiens (Human)
eriodictyol	P00918	CAH2_HUMAN	Homo sapiens (Human)
eriodictyol	P00734	THRB_HUMAN	Homo sapiens (Human)
eriodictyol	P08631	HCK_HUMAN	Homo sapiens (Human)
eriodictyol	P00915	CAH1_HUMAN	Homo sapiens (Human)
eriodictyol	P06276	CHLE_HUMAN	Homo sapiens (Human)
eriodictyol	P03372	ESR1_HUMAN	Homo sapiens (Human)
eriodictyol	P24941	CDK2_HUMAN	Homo sapiens (Human)
eriodictyol	P06401	PRGR_HUMAN	Homo sapiens (Human)
eriodictyol	P56817	BACE1_HUMAN	Homo sapiens (Human)
eriodictyol	P00390	GSHR_HUMAN	Homo sapiens (Human)
eriodictyol	P48736	PK3CG_HUMAN	Homo sapiens (Human)
eriodictyol	P11309	PIM1_HUMAN	Homo sapiens (Human)
eriodictyol	Q12884	SEPR_HUMAN	Homo sapiens (Human)
eriodictyol	P00491	PNPH_HUMAN	Homo sapiens (Human)
eriodictyol	P00533	EGFR_HUMAN	Homo sapiens (Human)
eriodictyol	P08842	STS_HUMAN	Homo sapiens (Human)
eriodictyol	P02652	APOA2_HUMAN	Homo sapiens (Human)
eriodictyol	P20248	CCNA2_HUMAN	Homo sapiens (Human)
eriodictyol	Q00534	CDK6_HUMAN	Homo sapiens (Human)
eriodictyol	P00751	CFAB_HUMAN	Homo sapiens (Human)
eriodictyol	P02768	ALBU_HUMAN	Homo sapiens (Human)
eriodictyol	P53779	MK10_HUMAN	Homo sapiens (Human)
eriodictyol	P09211	GSTP1_HUMAN	Homo sapiens (Human)
eriodictyol	P07339	CATD_HUMAN	Homo sapiens (Human)
eriodictyol	Q16740	CLPP_HUMAN	Homo sapiens (Human)
eriodictyol	P45983	MK08_HUMAN	Homo sapiens (Human)
eriodictyol	P04062	GLCM_HUMAN	Homo sapiens (Human)
eriodictyol	P35968	VGFR2_HUMAN	Homo sapiens (Human)
eriodictyol	P62508	ERR3_HUMAN	Homo sapiens (Human)
eriodictyol	Q00796	DHSO_HUMAN	Homo sapiens (Human)
eriodictyol	P02766	TTHY_HUMAN	Homo sapiens (Human)
eriodictyol	P54760	EPHB4_HUMAN	Homo sapiens (Human)

eriodictyol	O14757	CHK1_HUMAN	Homo sapiens (Human)
eriodictyol	P78536	ADA17_HUMAN	Homo sapiens (Human)
eriodictyol	P03950	ANGI_HUMAN	Homo sapiens (Human)
eriodictyol	O14965	AURKA_HUMAN	Homo sapiens (Human)
eriodictyol	O15382	BCAT2_HUMAN	Homo sapiens (Human)
eriodictyol	P29474	NOS3_HUMAN	Homo sapiens (Human)
eriodictyol	P00439	PH4H_HUMAN	Homo sapiens (Human)
eriodictyol	P14061	DHB1_HUMAN	Homo sapiens (Human)
eriodictyol	P04745	AMY1_HUMAN	Homo sapiens (Human)
eriodictyol	P27338	AOFB_HUMAN	Homo sapiens (Human)
eriodictyol	P11142	HSP7C_HUMAN	Homo sapiens (Human)
eriodictyol	P16152	CBR1_HUMAN	Homo sapiens (Human)
eriodictyol	P04278	SHBG_HUMAN	Homo sapiens (Human)
eriodictyol	Q13126	MTAP_HUMAN	Homo sapiens (Human)
eriodictyol	P78368	KC1G2_HUMAN	Homo sapiens (Human)
eriodictyol	Q08499	PDE4D_HUMAN	Homo sapiens (Human)
eriodictyol	P08758	ANXA5_HUMAN	Homo sapiens (Human)
eriodictyol	P06737	PYGL_HUMAN	Homo sapiens (Human)
eriodictyol	P14174	MIF_HUMAN	Homo sapiens (Human)
eriodictyol	P19793	RXRA_HUMAN	Homo sapiens (Human)
eriodictyol	Q16828	DUS6_HUMAN	Homo sapiens (Human)
eriodictyol	P07900	HS90A_HUMAN	Homo sapiens (Human)
eriodictyol	P15559	NQO1_HUMAN	Homo sapiens (Human)
eriodictyol	Q16539	MK14_HUMAN	Homo sapiens (Human)
eriodictyol	P45452	MMP13_HUMAN	Homo sapiens (Human)
eriodictyol	P15121	ALDR_HUMAN	Homo sapiens (Human)
eriodictyol	P28845	DHI1_HUMAN	Homo sapiens (Human)
eriodictyol	P18075	BMP7_HUMAN	Homo sapiens (Human)
eriodictyol	O15496	PA2GX_HUMAN	Homo sapiens (Human)
eriodictyol	P11766	ADHX_HUMAN	Homo sapiens (Human)
eriodictyol	P53350	PLK1_HUMAN	Homo sapiens (Human)
eriodictyol	P12931	SRC_HUMAN	Homo sapiens (Human)
eriodictyol	P17707	DCAM_HUMAN	Homo sapiens (Human)
eriodictyol	P11362	FGFR1_HUMAN	Homo sapiens (Human)
eriodictyol	O15530	PDPK1_HUMAN	Homo sapiens (Human)
eriodictyol	P23141	EST1_HUMAN	Homo sapiens (Human)
eriodictyol	P31749	AKT1_HUMAN	Homo sapiens (Human)
eriodictyol	P02774	VTDB_HUMAN	Homo sapiens (Human)
eriodictyol	P00374	DYR_HUMAN	Homo sapiens (Human)
eriodictyol	Q06520	ST2A1_HUMAN	Homo sapiens (Human)
eriodictyol	P00742	FA10_HUMAN	Homo sapiens (Human)
eriodictyol	P00746	CFAD_HUMAN	Homo sapiens (Human)
eriodictyol	P04818	TYSY_HUMAN	Homo sapiens (Human)
eriodictyol	Q9BY41	HDAC8_HUMAN	Homo sapiens (Human)
eriodictyol	P52732	KIF11_HUMAN	Homo sapiens (Human)
eriodictyol	P54577	SYYC_HUMAN	Homo sapiens (Human)
eriodictyol	Q03181	PPARD_HUMAN	Homo sapiens (Human)
eriodictyol	Q07343	PDE4B_HUMAN	Homo sapiens (Human)
Lucidusculine	P52732	KIF11_HUMAN	Homo sapiens (Human)
Lucidusculine	P12643	BMP2_HUMAN	Homo sapiens (Human)
Lucidusculine	P02652	APOA2_HUMAN	Homo sapiens (Human)
Lucidusculine	P02768	ALBU_HUMAN	Homo sapiens (Human)
Lucidusculine	P52895	AK1C2_HUMAN	Homo sapiens (Human)
Lucidusculine	P08842	STS_HUMAN	Homo sapiens (Human)
Lucidusculine	P00918	CAH2_HUMAN	Homo sapiens (Human)
Lucidusculine	P11309	PIM1_HUMAN	Homo sapiens (Human)
Lucidusculine	P20248	CCNA2_HUMAN	Homo sapiens (Human)
Lucidusculine	P62937	PPIA_HUMAN	Homo sapiens (Human)

Lucidusculine	P03372	ESR1_HUMAN	Homo sapiens (Human)
Lucidusculine	P53779	MK10_HUMAN	Homo sapiens (Human)
Lucidusculine	P00751	CFAB_HUMAN	Homo sapiens (Human)
Lucidusculine	P02766	TTHY_HUMAN	Homo sapiens (Human)
Lucidusculine	P02774	VTDB_HUMAN	Homo sapiens (Human)
Lucidusculine	P45983	MK08_HUMAN	Homo sapiens (Human)
Lucidusculine	P35398	RORA_HUMAN	Homo sapiens (Human)
Lucidusculine	O76054	S14L2_HUMAN	Homo sapiens (Human)
Lucidusculine	P56817	BACE1_HUMAN	Homo sapiens (Human)
Lucidusculine	P28482	MK01_HUMAN	Homo sapiens (Human)
Lucidusculine	P11086	PNMT_HUMAN	Homo sapiens (Human)
Lucidusculine	P20701	ITAL_HUMAN	Homo sapiens (Human)
Lucidusculine	P78536	ADA17_HUMAN	Homo sapiens (Human)
Lucidusculine	Q08499	PDE4D_HUMAN	Homo sapiens (Human)
Lucidusculine	Q16539	MK14_HUMAN	Homo sapiens (Human)
Lucidusculine	P06401	PRGR_HUMAN	Homo sapiens (Human)
Lucidusculine	P14061	DHB1_HUMAN	Homo sapiens (Human)
Lucidusculine	P10275	ANDR_HUMAN	Homo sapiens (Human)
Lucidusculine	P54760	EPHB4_HUMAN	Homo sapiens (Human)
Lucidusculine	P35968	VGFR2_HUMAN	Homo sapiens (Human)
Lucidusculine	P0DP23	CALM1_HUMAN	Homo sapiens (Human)
Lucidusculine	P0DP24	CALM2_HUMAN	Homo sapiens (Human)
Lucidusculine	P0DP25	CALM3_HUMAN	Homo sapiens (Human)
Lucidusculine	P49841	GSK3B_HUMAN	Homo sapiens (Human)
Lucidusculine	Q06124	PTN11_HUMAN	Homo sapiens (Human)
Lucidusculine	P24941	CDK2_HUMAN	Homo sapiens (Human)
Lucidusculine	P00734	THRB_HUMAN	Homo sapiens (Human)
Lucidusculine	Q07343	PDE4B_HUMAN	Homo sapiens (Human)
Lucidusculine	P34913	HYES_HUMAN	Homo sapiens (Human)
Lucidusculine	P28845	DHI1_HUMAN	Homo sapiens (Human)
Lucidusculine	Q06520	ST2A1_HUMAN	Homo sapiens (Human)
Lucidusculine	P53041	PPP5_HUMAN	Homo sapiens (Human)
Lucidusculine	P42768	WASP_HUMAN	Homo sapiens (Human)
Lucidusculine	Q00987	MDM2_HUMAN	Homo sapiens (Human)
Lucidusculine	P06276	CHLE_HUMAN	Homo sapiens (Human)
Lucidusculine	Q16828	DUS6_HUMAN	Homo sapiens (Human)
Lucidusculine	P00746	CFAD_HUMAN	Homo sapiens (Human)
Lucidusculine	Q9BY41	HDAC8_HUMAN	Homo sapiens (Human)
Lucidusculine	P08758	ANXA5_HUMAN	Homo sapiens (Human)
Lucidusculine	P08254	MMP3_HUMAN	Homo sapiens (Human)
Lucidusculine	P55210	CASP7_HUMAN	Homo sapiens (Human)
Lucidusculine	P00390	GSHR_HUMAN	Homo sapiens (Human)
Lucidusculine	P00325	ADH1B_HUMAN	Homo sapiens (Human)
Lucidusculine	P29474	NOS3_HUMAN	Homo sapiens (Human)
Lucidusculine	P11766	ADHX_HUMAN	Homo sapiens (Human)
Lucidusculine	P62508	ERR3_HUMAN	Homo sapiens (Human)
Lucidusculine	P00491	PNPH_HUMAN	Homo sapiens (Human)
Lucidusculine	P04278	SHBG_HUMAN	Homo sapiens (Human)
Lucidusculine	P04179	SODM_HUMAN	Homo sapiens (Human)
Lucidusculine	P15121	ALDR_HUMAN	Homo sapiens (Human)
Lucidusculine	P27338	AOFB_HUMAN	Homo sapiens (Human)
Lucidusculine	P15559	NQO1_HUMAN	Homo sapiens (Human)
Lucidusculine	P12931	SRC_HUMAN	Homo sapiens (Human)
Lucidusculine	P45452	MMP13_HUMAN	Homo sapiens (Human)
Lucidusculine	Q04828	AK1C1_HUMAN	Homo sapiens (Human)
Lucidusculine	P00533	EGFR_HUMAN	Homo sapiens (Human)
Lucidusculine	P09211	GSTP1_HUMAN	Homo sapiens (Human)
Lucidusculine	P54577	SYYC_HUMAN	Homo sapiens (Human)

Lucidusculine	O14757	CHK1_HUMAN	Homo sapiens (Human)
Lucidusculine	O15530	PDPK1_HUMAN	Homo sapiens (Human)
Lucidusculine	P30043	BLVRB_HUMAN	Homo sapiens (Human)
Lucidusculine	P21802	FGFR2_HUMAN	Homo sapiens (Human)
Lucidusculine	P08581	MET_HUMAN	Homo sapiens (Human)
Lucidusculine	P50579	MAP2_HUMAN	Homo sapiens (Human)
Lucidusculine	P18031	PTN1_HUMAN	Homo sapiens (Human)
Lucidusculine	Q16773	KAT1_HUMAN	Homo sapiens (Human)
Lucidusculine	P00747	PLMN_HUMAN	Homo sapiens (Human)
Lucidusculine	P49638	TTPA_HUMAN	Homo sapiens (Human)
Lucidusculine	P00326	ADH1G_HUMAN	Homo sapiens (Human)
Lucidusculine	O43617	TPPC3_HUMAN	Homo sapiens (Human)
Lucidusculine	P03950	ANGI_HUMAN	Homo sapiens (Human)
Lucidusculine	P00742	FA10_HUMAN	Homo sapiens (Human)
Lucidusculine	P49366	DHYS_HUMAN	Homo sapiens (Human)
Lucidusculine	P08263	GSTA1_HUMAN	Homo sapiens (Human)
Lucidusculine	P00374	DYR_HUMAN	Homo sapiens (Human)
Lucidusculine	P55263	ADK_HUMAN	Homo sapiens (Human)
Lucidusculine	P08235	MCR_HUMAN	Homo sapiens (Human)
Lucidusculine	P11712	CP2C9_HUMAN	Homo sapiens (Human)
Lucidusculine	P07900	HS90A_HUMAN	Homo sapiens (Human)
Lucidusculine	P04818	TYSY_HUMAN	Homo sapiens (Human)
Lucidusculine	P27487	DPP4_HUMAN	Homo sapiens (Human)
Lucidusculine	P01009	A1AT_HUMAN	Homo sapiens (Human)
Lucidusculine	P08709	FA7_HUMAN	Homo sapiens (Human)
Lucidusculine	P10828	THB_HUMAN	Homo sapiens (Human)
Lucidusculine	Q02127	PYRD_HUMAN	Homo sapiens (Human)
Lucidusculine	Q96RI1	NR1H4_HUMAN	Homo sapiens (Human)
Lucidusculine	P36897	TGFR1_HUMAN	Homo sapiens (Human)
Lucidusculine	P43405	KSYK_HUMAN	Homo sapiens (Human)
Lucidusculine	O15496	PA2GX_HUMAN	Homo sapiens (Human)
Lucidusculine	Q14541	HNF4G_HUMAN	Homo sapiens (Human)
Lucidusculine	P52333	JAK3_HUMAN	Homo sapiens (Human)
Lucidusculine	P55055	NR1H2_HUMAN	Homo sapiens (Human)
Lucidusculine	Q13133	NR1H3_HUMAN	Homo sapiens (Human)
Lucidusculine	Q08881	ITK_HUMAN	Homo sapiens (Human)
Lucidusculine	P37231	PPARG_HUMAN	Homo sapiens (Human)
Lucidusculine	P06239	LCK_HUMAN	Homo sapiens (Human)
Lucidusculine	P00439	PH4H_HUMAN	Homo sapiens (Human)
Lucidusculine	P08069	IGF1R_HUMAN	Homo sapiens (Human)
Lucidusculine	O75469	NR1I2_HUMAN	Homo sapiens (Human)
Lucidusculine	P49354	FNTA_HUMAN	Homo sapiens (Human)
Lucidusculine	P08246	ELNE_HUMAN	Homo sapiens (Human)
Lucidusculine	P49763	PLGF_HUMAN	Homo sapiens (Human)
Lucidusculine	P42330	AK1C3_HUMAN	Homo sapiens (Human)
Olitoriside	P09211	GSTP1_HUMAN	Homo sapiens (Human)
Olitoriside	P28482	MK01_HUMAN	Homo sapiens (Human)
Olitoriside	Q9NP99	TREM1_HUMAN	Homo sapiens (Human)
Olitoriside	P08842	STS_HUMAN	Homo sapiens (Human)
Olitoriside	P25774	CATS_HUMAN	Homo sapiens (Human)
Olitoriside	Q9BW91	NUDT9_HUMAN	Homo sapiens (Human)
Olitoriside	P00918	CAH2_HUMAN	Homo sapiens (Human)
Olitoriside	P02652	APOA2_HUMAN	Homo sapiens (Human)
Olitoriside	P07339	CATD_HUMAN	Homo sapiens (Human)
Olitoriside	P42330	AK1C3_HUMAN	Homo sapiens (Human)
Olitoriside	Q12884	SEPR_HUMAN	Homo sapiens (Human)
Olitoriside	P53582	MAP11_HUMAN	Homo sapiens (Human)
Olitoriside	P37173	TGFR2_HUMAN	Homo sapiens (Human)

Olitoriside	P04745	AMY1_HUMAN	Homo sapiens (Human)
Olitoriside	P08254	MMP3_HUMAN	Homo sapiens (Human)
Olitoriside	P42574	CASP3_HUMAN	Homo sapiens (Human)
Olitoriside	O14965	AURKA_HUMAN	Homo sapiens (Human)
Olitoriside	P04062	GLCM_HUMAN	Homo sapiens (Human)
Olitoriside	O14757	CHK1_HUMAN	Homo sapiens (Human)
Olitoriside	P62937	PPIA_HUMAN	Homo sapiens (Human)
Olitoriside	P00491	PNPH_HUMAN	Homo sapiens (Human)
Olitoriside	P53779	MK10_HUMAN	Homo sapiens (Human)
Olitoriside	P13598	ICAM2_HUMAN	Homo sapiens (Human)
Olitoriside	P02768	ALBU_HUMAN	Homo sapiens (Human)
Olitoriside	P00533	EGFR_HUMAN	Homo sapiens (Human)
Olitoriside	Q07343	PDE4B_HUMAN	Homo sapiens (Human)
Olitoriside	P00915	CAH1_HUMAN	Homo sapiens (Human)
Olitoriside	P27338	AOFB_HUMAN	Homo sapiens (Human)
Olitoriside	P06276	CHLE_HUMAN	Homo sapiens (Human)
Olitoriside	Q92731	ESR2_HUMAN	Homo sapiens (Human)
Olitoriside	P02766	TTHY_HUMAN	Homo sapiens (Human)
Olitoriside	Q16539	MK14_HUMAN	Homo sapiens (Human)
Olitoriside	P11142	HSP7C_HUMAN	Homo sapiens (Human)
Olitoriside	Q13126	MTAP_HUMAN	Homo sapiens (Human)
Olitoriside	Q8NBQ5	DHB11_HUMAN	Homo sapiens (Human)
Olitoriside	Q05315	LEG10_HUMAN	Homo sapiens (Human)
Olitoriside	P24941	CDK2_HUMAN	Homo sapiens (Human)
Olitoriside	P62942	FKB1A_HUMAN	Homo sapiens (Human)
Olitoriside	P23141	EST1_HUMAN	Homo sapiens (Human)
Olitoriside	P12643	BMP2_HUMAN	Homo sapiens (Human)
Olitoriside	O43570	CAH12_HUMAN	Homo sapiens (Human)
Olitoriside	P37231	PPARG_HUMAN	Homo sapiens (Human)
Olitoriside	P49137	MAPK2_HUMAN	Homo sapiens (Human)
Olitoriside	O60911	CATL2_HUMAN	Homo sapiens (Human)
Olitoriside	Q9BZR6	RTN4R_HUMAN	Homo sapiens (Human)
Olitoriside	P55055	NR1H2_HUMAN	Homo sapiens (Human)
Olitoriside	P11309	PIM1_HUMAN	Homo sapiens (Human)
Olitoriside	P00751	CFAB_HUMAN	Homo sapiens (Human)
Olitoriside	P35968	VGFR2_HUMAN	Homo sapiens (Human)
Olitoriside	P10275	ANDR_HUMAN	Homo sapiens (Human)
Olitoriside	P45983	MK08_HUMAN	Homo sapiens (Human)
Olitoriside	P07858	CATB_HUMAN	Homo sapiens (Human)
Olitoriside	P00749	UROK_HUMAN	Homo sapiens (Human)
Olitoriside	P54760	EPHB4_HUMAN	Homo sapiens (Human)
Olitoriside	P26196	DDX6_HUMAN	Homo sapiens (Human)
Olitoriside	P06401	PRGR_HUMAN	Homo sapiens (Human)
Olitoriside	P56817	BACE1_HUMAN	Homo sapiens (Human)
Olitoriside	P20248	CCNA2_HUMAN	Homo sapiens (Human)
Olitoriside	P19367	HXK1_HUMAN	Homo sapiens (Human)
Olitoriside	P52895	AK1C2_HUMAN	Homo sapiens (Human)
Olitoriside	P15121	ALDR_HUMAN	Homo sapiens (Human)
Olitoriside	P14061	DHB1_HUMAN	Homo sapiens (Human)
Olitoriside	P24071	FCAR_HUMAN	Homo sapiens (Human)
Olitoriside	P11511	CP19A_HUMAN	Homo sapiens (Human)
Olitoriside	P52732	KIF11_HUMAN	Homo sapiens (Human)
Olitoriside	P03950	ANGI_HUMAN	Homo sapiens (Human)
Olitoriside	Q13231	CHIT1_HUMAN	Homo sapiens (Human)
Olitoriside	Q13370	PDE3B_HUMAN	Homo sapiens (Human)
Olitoriside	P18075	BMP7_HUMAN	Homo sapiens (Human)
Olitoriside	P47929	LEG7_HUMAN	Homo sapiens (Human)
Olitoriside	P00746	CFAD_HUMAN	Homo sapiens (Human)

Olitoriside	P63316	TNNC1_HUMAN	Homo sapiens (Human)
Olitoriside	Q06520	ST2A1_HUMAN	Homo sapiens (Human)
Olitoriside	P12931	SRC_HUMAN	Homo sapiens (Human)
Olitoriside	P07900	HS90A_HUMAN	Homo sapiens (Human)
Olitoriside	P04278	SHBG_HUMAN	Homo sapiens (Human)
Olitoriside	P04818	TYSY_HUMAN	Homo sapiens (Human)
Olitoriside	P03372	ESR1_HUMAN	Homo sapiens (Human)
Olitoriside	P02774	VTDB_HUMAN	Homo sapiens (Human)
Olitoriside	P48449	ERG7_HUMAN	Homo sapiens (Human)
Olitoriside	P00736	C1R_HUMAN	Homo sapiens (Human)
Olitoriside	P29474	NOS3_HUMAN	Homo sapiens (Human)
Olitoriside	P14174	MIF_HUMAN	Homo sapiens (Human)
Olitoriside	P27487	DPP4_HUMAN	Homo sapiens (Human)
Olitoriside	Q02127	PYRD_HUMAN	Homo sapiens (Human)
Olitoriside	Q00796	DHSO_HUMAN	Homo sapiens (Human)
Olitoriside	P08235	MCR_HUMAN	Homo sapiens (Human)
Olitoriside	P08758	ANXA5_HUMAN	Homo sapiens (Human)
Olitoriside	P08631	HCK_HUMAN	Homo sapiens (Human)
Olitoriside	P01709	LV208_HUMAN	Homo sapiens (Human)
Olitoriside	Q08499	PDE4D_HUMAN	Homo sapiens (Human)
Olitoriside	P00374	DYR_HUMAN	Homo sapiens (Human)
Olitoriside	P02743	SAMP_HUMAN	Homo sapiens (Human)
Olitoriside	P22894	MMP8_HUMAN	Homo sapiens (Human)
Olitoriside	P29218	IMPA1_HUMAN	Homo sapiens (Human)
Olitoriside	P35398	RORA_HUMAN	Homo sapiens (Human)
Olitoriside	P36897	TGFR1_HUMAN	Homo sapiens (Human)
Olitoriside	P17707	DCAM_HUMAN	Homo sapiens (Human)
Olitoriside	P45452	MMP13_HUMAN	Homo sapiens (Human)
Olitoriside	P06737	PYGL_HUMAN	Homo sapiens (Human)
Olitoriside	P00390	GSHR_HUMAN	Homo sapiens (Human)
Olitoriside	P25311	ZA2G_HUMAN	Homo sapiens (Human)
Olitoriside	P19971	TYPH_HUMAN	Homo sapiens (Human)
Olitoriside	P43235	CATK_HUMAN	Homo sapiens (Human)
Olitoriside	P00742	FA10_HUMAN	Homo sapiens (Human)
Olitoriside	P43155	CACP_HUMAN	Homo sapiens (Human)
Olitoriside	O15496	PA2GX_HUMAN	Homo sapiens (Human)
Olitoriside	P53355	DAPK1_HUMAN	Homo sapiens (Human)
Olitoriside	P23526	SAHH_HUMAN	Homo sapiens (Human)
Olitoriside	P00734	THRB_HUMAN	Homo sapiens (Human)
Olitoriside	P53041	PPP5_HUMAN	Homo sapiens (Human)
Olitoriside	O15530	PDPK1_HUMAN	Homo sapiens (Human)
Olitoriside	Q99933	BAG1_HUMAN	Homo sapiens (Human)
Olitoriside	P05019	IGF1_HUMAN	Homo sapiens (Human)
Olitoriside	P28845	DHI1_HUMAN	Homo sapiens (Human)
Olitoriside	P00439	PH4H_HUMAN	Homo sapiens (Human)
Olitoriside	Q07912	ACK1_HUMAN	Homo sapiens (Human)
Olitoriside	P00325	ADH1B_HUMAN	Homo sapiens (Human)
Olitoriside	P08069	IGF1R_HUMAN	Homo sapiens (Human)
Olitoriside	P31749	AKT1_HUMAN	Homo sapiens (Human)
Olitoriside	P15559	NQO1_HUMAN	Homo sapiens (Human)
Olitoriside	P00797	RENI_HUMAN	Homo sapiens (Human)
Olitoriside	Q9P2W7	B3GA1_HUMAN	Homo sapiens (Human)
Olitoriside	P49841	GSK3B_HUMAN	Homo sapiens (Human)
Olitoriside	Q9NVS9	PNPO_HUMAN	Homo sapiens (Human)
Olitoriside	Q96T66	NMNA3_HUMAN	Homo sapiens (Human)
Olitoriside	P19793	RXRA_HUMAN	Homo sapiens (Human)
Olitoriside	P39900	MMP12_HUMAN	Homo sapiens (Human)
Olitoriside	O76074	PDE5A_HUMAN	Homo sapiens (Human)

Olitoriside	P62508	ERR3_HUMAN	Homo sapiens (Human)
Olitoriside	P00326	ADH1G_HUMAN	Homo sapiens (Human)
Olitoriside	O15382	BCAT2_HUMAN	Homo sapiens (Human)
Olitoriside	P05091	ALDH2_HUMAN	Homo sapiens (Human)
Olitoriside	P15056	BRAF_HUMAN	Homo sapiens (Human)
Olitoriside	P07686	HEXB_HUMAN	Homo sapiens (Human)
Olitoriside	Q9BY41	HDAC8_HUMAN	Homo sapiens (Human)
Olitoriside	Q16828	DUS6_HUMAN	Homo sapiens (Human)
Olitoriside	P11086	PNMT_HUMAN	Homo sapiens (Human)
Olitoriside	P49354	FNTA_HUMAN	Homo sapiens (Human)
Olitoriside	P15153	RAC2_HUMAN	Homo sapiens (Human)
Olitoriside	P49789	FHIT_HUMAN	Homo sapiens (Human)
Olitoriside	Q10588	BST1_HUMAN	Homo sapiens (Human)
Olitoriside	Q13133	NR1H3_HUMAN	Homo sapiens (Human)
Olitoriside	P51570	GALK1_HUMAN	Homo sapiens (Human)
Olitoriside	P11362	FGFR1_HUMAN	Homo sapiens (Human)
Olitoriside	Q00534	CDK6_HUMAN	Homo sapiens (Human)
Olitoriside	P08246	ELNE_HUMAN	Homo sapiens (Human)
Olitoriside	P49638	TTPA_HUMAN	Homo sapiens (Human)
syringaresinol diglucoside_qt	P11309	PIM1_HUMAN	Homo sapiens (Human)
syringaresinol diglucoside_qt	P10275	ANDR_HUMAN	Homo sapiens (Human)
syringaresinol diglucoside_qt	P00918	CAH2_HUMAN	Homo sapiens (Human)
syringaresinol diglucoside_qt	P42574	CASP3_HUMAN	Homo sapiens (Human)
syringaresinol diglucoside_qt	P56817	BACE1_HUMAN	Homo sapiens (Human)
syringaresinol diglucoside_qt	Q16740	CLPP_HUMAN	Homo sapiens (Human)
syringaresinol diglucoside_qt	P00491	PNPH_HUMAN	Homo sapiens (Human)
syringaresinol diglucoside_qt	P02766	TTHY_HUMAN	Homo sapiens (Human)
syringaresinol diglucoside_qt	P09211	GSTP1_HUMAN	Homo sapiens (Human)
syringaresinol diglucoside_qt	P78536	ADA17_HUMAN	Homo sapiens (Human)
syringaresinol diglucoside_qt	P06276	CHLE_HUMAN	Homo sapiens (Human)
syringaresinol diglucoside_qt	P00746	CFAD_HUMAN	Homo sapiens (Human)
syringaresinol diglucoside_qt	P53779	MK10_HUMAN	Homo sapiens (Human)
syringaresinol diglucoside_qt	P35968	VGFR2_HUMAN	Homo sapiens (Human)
syringaresinol diglucoside_qt	P04278	SHBG_HUMAN	Homo sapiens (Human)
syringaresinol diglucoside_qt	O60911	CATL2_HUMAN	Homo sapiens (Human)
syringaresinol diglucoside_qt	Q8NBQ5	DHB11_HUMAN	Homo sapiens (Human)
syringaresinol diglucoside_qt	P25774	CATS_HUMAN	Homo sapiens (Human)
syringaresinol diglucoside_qt	P06401	PRGR_HUMAN	Homo sapiens (Human)
syringaresinol diglucoside_qt	P62937	PPIA_HUMAN	Homo sapiens (Human)
syringaresinol diglucoside_qt	P07900	HS90A_HUMAN	Homo sapiens (Human)
syringaresinol diglucoside_qt	P04062	GLCM_HUMAN	Homo sapiens (Human)
syringaresinol diglucoside_qt	P42330	AK1C3_HUMAN	Homo sapiens (Human)
syringaresinol diglucoside_qt	P14174	MIF_HUMAN	Homo sapiens (Human)
syringaresinol diglucoside_qt	O14757	CHK1_HUMAN	Homo sapiens (Human)
syringaresinol diglucoside_qt	P62508	ERR3_HUMAN	Homo sapiens (Human)
syringaresinol diglucoside_qt	P14061	DHB1_HUMAN	Homo sapiens (Human)
syringaresinol diglucoside_qt	P08069	IGF1R_HUMAN	Homo sapiens (Human)
syringaresinol diglucoside_qt	Q06520	ST2A1_HUMAN	Homo sapiens (Human)
syringaresinol diglucoside_qt	P45452	MMP13_HUMAN	Homo sapiens (Human)
syringaresinol diglucoside_qt	P11362	FGFR1_HUMAN	Homo sapiens (Human)
syringaresinol diglucoside_qt	Q08499	PDE4D_HUMAN	Homo sapiens (Human)
syringaresinol diglucoside_qt	P07339	CATD_HUMAN	Homo sapiens (Human)
syringaresinol diglucoside_qt	O15496	PA2GX_HUMAN	Homo sapiens (Human)
syringaresinol diglucoside_qt	Q02127	PYRD_HUMAN	Homo sapiens (Human)
syringaresinol diglucoside_qt	P35398	RORA_HUMAN	Homo sapiens (Human)
syringaresinol diglucoside_qt	P45983	MK08_HUMAN	Homo sapiens (Human)
syringaresinol diglucoside_qt	P50579	MAP2_HUMAN	Homo sapiens (Human)
syringaresinol diglucoside_qt	P19793	RXRA_HUMAN	Homo sapiens (Human)

syringaresinol diglucoside_qt	Q16539	MK14_HUMAN	Homo sapiens (Human)
syringaresinol diglucoside_qt	P35221	CTNA1_HUMAN	Homo sapiens (Human)
syringaresinol diglucoside_qt	Q9NP99	TREM1_HUMAN	Homo sapiens (Human)
syringaresinol diglucoside_qt	P08631	HCK_HUMAN	Homo sapiens (Human)
syringaresinol diglucoside_qt	P02774	VTDB_HUMAN	Homo sapiens (Human)
syringaresinol diglucoside_qt	P54760	EPHB4_HUMAN	Homo sapiens (Human)
syringaresinol diglucoside_qt	P20248	CCNA2_HUMAN	Homo sapiens (Human)
syringaresinol diglucoside_qt	P63316	TNNC1_HUMAN	Homo sapiens (Human)
syringaresinol diglucoside_qt	Q07343	PDE4B_HUMAN	Homo sapiens (Human)
syringaresinol diglucoside_qt	P48736	PK3CG_HUMAN	Homo sapiens (Human)
syringaresinol diglucoside_qt	P27338	AOFB_HUMAN	Homo sapiens (Human)
syringaresinol diglucoside_qt	O14965	AURKA_HUMAN	Homo sapiens (Human)
syringaresinol diglucoside_qt	P00533	EGFR_HUMAN	Homo sapiens (Human)
syringaresinol diglucoside_qt	P00374	DYR_HUMAN	Homo sapiens (Human)
syringaresinol diglucoside_qt	P23141	EST1_HUMAN	Homo sapiens (Human)
syringaresinol diglucoside_qt	P15056	BRAF_HUMAN	Homo sapiens (Human)
syringaresinol diglucoside_qt	P00390	GSHR_HUMAN	Homo sapiens (Human)
syringaresinol diglucoside_qt	Q12884	SEPR_HUMAN	Homo sapiens (Human)
syringaresinol diglucoside_qt	P16109	LYAM3_HUMAN	Homo sapiens (Human)
syringaresinol diglucoside_qt	P11511	CP19A_HUMAN	Homo sapiens (Human)
syringaresinol diglucoside_qt	P04745	AMY1_HUMAN	Homo sapiens (Human)
syringaresinol diglucoside_qt	Q13126	MTAP_HUMAN	Homo sapiens (Human)
syringaresinol diglucoside_qt	P28482	MK01_HUMAN	Homo sapiens (Human)
syringaresinol diglucoside_qt	P03950	ANGI_HUMAN	Homo sapiens (Human)
syringaresinol diglucoside_qt	P53041	PPP5_HUMAN	Homo sapiens (Human)
syringaresinol diglucoside_qt	P03372	ESR1_HUMAN	Homo sapiens (Human)
syringaresinol diglucoside_qt	P49763	PLGF_HUMAN	Homo sapiens (Human)
syringaresinol diglucoside_qt	Q00796	DHSO_HUMAN	Homo sapiens (Human)
syringaresinol diglucoside_qt	P0DP23	CALM1_HUMAN	Homo sapiens (Human)
syringaresinol diglucoside_qt	P0DP24	CALM2_HUMAN	Homo sapiens (Human)
syringaresinol diglucoside_qt	P0DP25	CALM3_HUMAN	Homo sapiens (Human)
syringaresinol diglucoside_qt	P54577	SYYC_HUMAN	Homo sapiens (Human)
syringaresinol diglucoside_qt	Q00987	MDM2_HUMAN	Homo sapiens (Human)
syringaresinol diglucoside_qt	P39900	MMP12_HUMAN	Homo sapiens (Human)
syringaresinol diglucoside_qt	P04818	TYSY_HUMAN	Homo sapiens (Human)
syringaresinol diglucoside_qt	P02768	ALBU_HUMAN	Homo sapiens (Human)
syringaresinol diglucoside_qt	P22894	MMP8_HUMAN	Homo sapiens (Human)
syringaresinol diglucoside_qt	P55055	NR1H2_HUMAN	Homo sapiens (Human)
syringaresinol diglucoside_qt	P53582	MAP11_HUMAN	Homo sapiens (Human)
syringaresinol diglucoside_qt	P00742	FA10_HUMAN	Homo sapiens (Human)
syringaresinol diglucoside_qt	P24941	CDK2_HUMAN	Homo sapiens (Human)
syringaresinol diglucoside_qt	P11712	CP2C9_HUMAN	Homo sapiens (Human)
syringaresinol diglucoside_qt	P08709	FA7_HUMAN	Homo sapiens (Human)
syringaresinol diglucoside_qt	Q13370	PDE3B_HUMAN	Homo sapiens (Human)
syringaresinol diglucoside_qt	P21802	FGFR2_HUMAN	Homo sapiens (Human)
syringaresinol diglucoside_qt	P00734	THRB_HUMAN	Homo sapiens (Human)
syringaresinol diglucoside_qt	P52732	KIF11_HUMAN	Homo sapiens (Human)
syringaresinol diglucoside_qt	Q13133	NR1H3_HUMAN	Homo sapiens (Human)
syringaresinol diglucoside_qt	P29474	NOS3_HUMAN	Homo sapiens (Human)
syringaresinol diglucoside_qt	P00439	PH4H_HUMAN	Homo sapiens (Human)
syringaresinol diglucoside_qt	P16152	CBR1_HUMAN	Homo sapiens (Human)
syringaresinol diglucoside_qt	O60760	HPGDS_HUMAN	Homo sapiens (Human)
syringaresinol diglucoside_qt	P27487	DPP4_HUMAN	Homo sapiens (Human)
syringaresinol diglucoside_qt	P11142	HSP7C_HUMAN	Homo sapiens (Human)
syringaresinol diglucoside_qt	P49841	GSK3B_HUMAN	Homo sapiens (Human)
syringaresinol diglucoside_qt	O15530	PDPK1_HUMAN	Homo sapiens (Human)
syringaresinol diglucoside_qt	O76074	PDE5A_HUMAN	Homo sapiens (Human)
syringaresinol diglucoside_qt	P49354	FNTA_HUMAN	Homo sapiens (Human)

syringaresinol diglucoside_qt	P00797	RENI_HUMAN	Homo sapiens (Human)
syringaresinol diglucoside_qt	Q08881	ITK_HUMAN	Homo sapiens (Human)
syringaresinol diglucoside_qt	P19971	TYPH_HUMAN	Homo sapiens (Human)
syringaresinol diglucoside_qt	P43235	CATK_HUMAN	Homo sapiens (Human)
syringaresinol diglucoside_qt	P08235	MCR_HUMAN	Homo sapiens (Human)
syringaresinol diglucoside_qt	Q00534	CDK6_HUMAN	Homo sapiens (Human)
syringaresinol diglucoside_qt	P04179	SODM_HUMAN	Homo sapiens (Human)
syringaresinol diglucoside_qt	P17707	DCAM_HUMAN	Homo sapiens (Human)
syringaresinol diglucoside_qt	P06737	PYGL_HUMAN	Homo sapiens (Human)
syringaresinol diglucoside_qt	Q01469	FABP5_HUMAN	Homo sapiens (Human)
syringaresinol diglucoside_qt	P25311	ZA2G_HUMAN	Homo sapiens (Human)
syringaresinol diglucoside_qt	P00325	ADH1B_HUMAN	Homo sapiens (Human)
syringaresinol diglucoside_qt	P43405	KSYK_HUMAN	Homo sapiens (Human)
syringaresinol diglucoside_qt	P28845	DHI1_HUMAN	Homo sapiens (Human)
syringaresinol diglucoside_qt	P07858	CATB_HUMAN	Homo sapiens (Human)
syringaresinol diglucoside_qt	P47929	LEG7_HUMAN	Homo sapiens (Human)
syringaresinol diglucoside_qt	P16083	NQO2_HUMAN	Homo sapiens (Human)
syringaresinol diglucoside_qt	P12931	SRC_HUMAN	Homo sapiens (Human)
syringaresinol diglucoside_qt	P09874	PARP1_HUMAN	Homo sapiens (Human)
syringaresinol diglucoside_qt	P42768	WASP_HUMAN	Homo sapiens (Human)
syringaresinol diglucoside_qt	P00751	CFAB_HUMAN	Homo sapiens (Human)
syringaresinol diglucoside_qt	P15121	ALDR_HUMAN	Homo sapiens (Human)
syringaresinol diglucoside_qt	P43155	CACP_HUMAN	Homo sapiens (Human)
syringaresinol diglucoside_qt	Q04828	AK1C1_HUMAN	Homo sapiens (Human)
syringaresinol diglucoside_qt	P08254	MMP3_HUMAN	Homo sapiens (Human)
syringaresinol diglucoside_qt	P15559	NQO1_HUMAN	Homo sapiens (Human)
syringaresinol diglucoside_qt	Q13231	CHIT1_HUMAN	Homo sapiens (Human)
syringaresinol diglucoside_qt	P78368	KC1G2_HUMAN	Homo sapiens (Human)
syringaresinol diglucoside_qt	P11766	ADHX_HUMAN	Homo sapiens (Human)
syringaresinol diglucoside_qt	Q9BY41	HDAC8_HUMAN	Homo sapiens (Human)
syringaresinol diglucoside_qt	P18075	BMP7_HUMAN	Homo sapiens (Human)
syringaresinol diglucoside_qt	P11586	C1TC_HUMAN	Homo sapiens (Human)
syringaresinol diglucoside_qt	P68400	CSK21_HUMAN	Homo sapiens (Human)
syringaresinol diglucoside_qt	O15382	BCAT2_HUMAN	Homo sapiens (Human)
syringaresinol diglucoside_qt	P10827	THA_HUMAN	Homo sapiens (Human)
syringaresinol diglucoside_qt	P20701	ITAL_HUMAN	Homo sapiens (Human)
syringaresinol diglucoside_qt	P08246	ELNE_HUMAN	Homo sapiens (Human)
syringaresinol diglucoside_qt	P06239	LCK_HUMAN	Homo sapiens (Human)
syringaresinol diglucoside_qt	P18031	PTN1_HUMAN	Homo sapiens (Human)
syringaresinol diglucoside_qt	P48449	ERG7_HUMAN	Homo sapiens (Human)
syringaresinol diglucoside_qt	P55263	ADK_HUMAN	Homo sapiens (Human)
syringaresinol diglucoside_qt	P10828	THB_HUMAN	Homo sapiens (Human)
syringaresinol diglucoside_qt	P08581	MET_HUMAN	Homo sapiens (Human)
syringaresinol diglucoside_qt	P02753	RET4_HUMAN	Homo sapiens (Human)
syringaresinol diglucoside_qt	P06126	CD1A_HUMAN	Homo sapiens (Human)
syringaresinol diglucoside_qt	P53350	PLK1_HUMAN	Homo sapiens (Human)
syringaresinol diglucoside_qt	P15090	FABP4_HUMAN	Homo sapiens (Human)
syringaresinol diglucoside_qt	P08263	GSTA1_HUMAN	Homo sapiens (Human)
syringaresinol diglucoside_qt	P30043	BLVRB_HUMAN	Homo sapiens (Human)
syringaresinol diglucoside_qt	P37231	PPARG_HUMAN	Homo sapiens (Human)
syringaresinol diglucoside_qt	P41240	CSK_HUMAN	Homo sapiens (Human)
syringaresinol diglucoside_qt	Q9NVS9	PNPO_HUMAN	Homo sapiens (Human)
syringaresinol diglucoside_qt	P52333	JAK3_HUMAN	Homo sapiens (Human)
syringaresinol diglucoside_qt	Q07869	PPARA_HUMAN	Homo sapiens (Human)
syringaresinol diglucoside_qt	P52895	AK1C2_HUMAN	Homo sapiens (Human)
syringaresinol diglucoside_qt	P05019	IGF1_HUMAN	Homo sapiens (Human)
syringaresinol diglucoside_qt	Q9P286	PAK5_HUMAN	Homo sapiens (Human)
syringaresinol diglucoside_qt	P53355	DAPK1_HUMAN	Homo sapiens (Human)

syringaresinol diglucoside_qt	P02743	SAMP_HUMAN	Homo sapiens (Human)
syringaresinol diglucoside_qt	P34896	GLYC_HUMAN	Homo sapiens (Human)
syringaresinol diglucoside_qt	P27707	DCK_HUMAN	Homo sapiens (Human)
syringaresinol diglucoside_qt	Q96T66	NMNA3_HUMAN	Homo sapiens (Human)
syringaresinol diglucoside_qt	O75469	NR1I2_HUMAN	Homo sapiens (Human)
syringaresinol diglucoside_qt	P01709	LV208_HUMAN	Homo sapiens (Human)
Olitoriside_qt	P52895	AK1C2_HUMAN	Homo sapiens (Human)
Olitoriside_qt	P00918	CAH2_HUMAN	Homo sapiens (Human)
Olitoriside_qt	P49137	MAPK2_HUMAN	Homo sapiens (Human)
Olitoriside_qt	Q16539	MK14_HUMAN	Homo sapiens (Human)
Olitoriside_qt	P00491	PNPH_HUMAN	Homo sapiens (Human)
Olitoriside_qt	P12643	BMP2_HUMAN	Homo sapiens (Human)
Olitoriside_qt	P11309	PIM1_HUMAN	Homo sapiens (Human)
Olitoriside_qt	Q8NBQ5	DHB11_HUMAN	Homo sapiens (Human)
Olitoriside_qt	Q9NP99	TREM1_HUMAN	Homo sapiens (Human)
Olitoriside_qt	P10275	ANDR_HUMAN	Homo sapiens (Human)
Olitoriside_qt	P04062	GLCM_HUMAN	Homo sapiens (Human)
Olitoriside_qt	P56817	BACE1_HUMAN	Homo sapiens (Human)
Olitoriside_qt	P07339	CATD_HUMAN	Homo sapiens (Human)
Olitoriside_qt	P11511	CP19A_HUMAN	Homo sapiens (Human)
Olitoriside_qt	P55055	NR1H2_HUMAN	Homo sapiens (Human)
Olitoriside_qt	Q16740	CLPP_HUMAN	Homo sapiens (Human)
Olitoriside_qt	Q06520	ST2A1_HUMAN	Homo sapiens (Human)
Olitoriside_qt	P04179	SODM_HUMAN	Homo sapiens (Human)
Olitoriside_qt	Q00796	DHSO_HUMAN	Homo sapiens (Human)
Olitoriside_qt	P55210	CASP7_HUMAN	Homo sapiens (Human)
Olitoriside_qt	Q07343	PDE4B_HUMAN	Homo sapiens (Human)
Olitoriside_qt	P00533	EGFR_HUMAN	Homo sapiens (Human)
Olitoriside_qt	O14757	CHK1_HUMAN	Homo sapiens (Human)
Olitoriside_qt	P00746	CFAD_HUMAN	Homo sapiens (Human)
Olitoriside_qt	P12931	SRC_HUMAN	Homo sapiens (Human)
Olitoriside_qt	P03372	ESR1_HUMAN	Homo sapiens (Human)
Olitoriside_qt	P14061	DHB1_HUMAN	Homo sapiens (Human)
Olitoriside_qt	P62937	PPIA_HUMAN	Homo sapiens (Human)
Olitoriside_qt	P04278	SHBG_HUMAN	Homo sapiens (Human)
Olitoriside_qt	P15121	ALDR_HUMAN	Homo sapiens (Human)
Olitoriside_qt	O15496	PA2GX_HUMAN	Homo sapiens (Human)
Olitoriside_qt	P04818	TYSY_HUMAN	Homo sapiens (Human)
Olitoriside_qt	P08758	ANXA5_HUMAN	Homo sapiens (Human)
Olitoriside_qt	P02766	TTHY_HUMAN	Homo sapiens (Human)
Olitoriside_qt	P02774	VTDB_HUMAN	Homo sapiens (Human)
Olitoriside_qt	P00751	CFAB_HUMAN	Homo sapiens (Human)
Olitoriside_qt	P06401	PRGR_HUMAN	Homo sapiens (Human)
Olitoriside_qt	P08235	MCR_HUMAN	Homo sapiens (Human)
Olitoriside_qt	P36897	TGFR1_HUMAN	Homo sapiens (Human)
Olitoriside_qt	P29474	NOS3_HUMAN	Homo sapiens (Human)
Olitoriside_qt	P00374	DYR_HUMAN	Homo sapiens (Human)
Olitoriside_qt	P37231	PPARG_HUMAN	Homo sapiens (Human)
Olitoriside_qt	Q13126	MTAP_HUMAN	Homo sapiens (Human)
Olitoriside_qt	O15530	PDPK1_HUMAN	Homo sapiens (Human)
Olitoriside_qt	P53041	PPP5_HUMAN	Homo sapiens (Human)
Olitoriside_qt	Q16828	DUS6_HUMAN	Homo sapiens (Human)
Olitoriside_qt	P19793	RXRA_HUMAN	Homo sapiens (Human)
Olitoriside_qt	Q02127	PYRD_HUMAN	Homo sapiens (Human)
Lucidumoside D_qt	P53779	MK10_HUMAN	Homo sapiens (Human)
Lucidumoside D_qt	P11309	PIM1_HUMAN	Homo sapiens (Human)
Lucidumoside D_qt	P02766	TTHY_HUMAN	Homo sapiens (Human)
Lucidumoside D_qt	P62937	PPIA_HUMAN	Homo sapiens (Human)

Lucidumoside D_qt	P26196	DDX6_HUMAN	Homo sapiens (Human)
Lucidumoside D_qt	P20248	CCNA2_HUMAN	Homo sapiens (Human)
Lucidumoside D_qt	O14757	CHK1_HUMAN	Homo sapiens (Human)
Lucidumoside D_qt	P23946	CMA1_HUMAN	Homo sapiens (Human)
Lucidumoside D_qt	P54760	EPHB4_HUMAN	Homo sapiens (Human)
Lucidumoside D_qt	P45983	MK08_HUMAN	Homo sapiens (Human)
Lucidumoside D_qt	P55055	NR1H2_HUMAN	Homo sapiens (Human)
Lucidumoside D_qt	P00918	CAH2_HUMAN	Homo sapiens (Human)
Lucidumoside D_qt	P08254	MMP3_HUMAN	Homo sapiens (Human)
Lucidumoside D_qt	P09211	GSTP1_HUMAN	Homo sapiens (Human)
Lucidumoside D_qt	Q92731	ESR2_HUMAN	Homo sapiens (Human)
Lucidumoside D_qt	P07339	CATD_HUMAN	Homo sapiens (Human)
Lucidumoside D_qt	P06276	CHLE_HUMAN	Homo sapiens (Human)
Lucidumoside D_qt	P62942	FKB1A_HUMAN	Homo sapiens (Human)
Lucidumoside D_qt	P10275	ANDR_HUMAN	Homo sapiens (Human)
Lucidumoside D_qt	P04062	GLCM_HUMAN	Homo sapiens (Human)
Lucidumoside D_qt	P00491	PNPH_HUMAN	Homo sapiens (Human)
Lucidumoside D_qt	P00751	CFAB_HUMAN	Homo sapiens (Human)
Lucidumoside D_qt	P28482	MK01_HUMAN	Homo sapiens (Human)
Lucidumoside D_qt	Q9NP99	TREM1_HUMAN	Homo sapiens (Human)
Lucidumoside D_qt	P03372	ESR1_HUMAN	Homo sapiens (Human)
Lucidumoside D_qt	P53582	MAP11_HUMAN	Homo sapiens (Human)
Lucidumoside D_qt	P15121	ALDR_HUMAN	Homo sapiens (Human)
Lucidumoside D_qt	P52732	KIF11_HUMAN	Homo sapiens (Human)
Lucidumoside D_qt	P11511	CP19A_HUMAN	Homo sapiens (Human)
Lucidumoside D_qt	P24941	CDK2_HUMAN	Homo sapiens (Human)
Lucidumoside D_qt	P37231	PPARG_HUMAN	Homo sapiens (Human)
Lucidumoside D_qt	P08842	STS_HUMAN	Homo sapiens (Human)
Lucidumoside D_qt	P27338	AOFB_HUMAN	Homo sapiens (Human)
Lucidumoside D_qt	P00533	EGFR_HUMAN	Homo sapiens (Human)
Lucidumoside D_qt	P02768	ALBU_HUMAN	Homo sapiens (Human)
Lucidumoside D_qt	P11142	HSP7C_HUMAN	Homo sapiens (Human)
Lucidumoside D_qt	P06401	PRGR_HUMAN	Homo sapiens (Human)
Lucidumoside D_qt	P07858	CATB_HUMAN	Homo sapiens (Human)
Lucidumoside D_qt	P08758	ANXA5_HUMAN	Homo sapiens (Human)
Lucidumoside D_qt	P03950	ANGI_HUMAN	Homo sapiens (Human)
Lucidumoside D_qt	P00734	THRB_HUMAN	Homo sapiens (Human)
Lucidumoside D_qt	P32320	CDD_HUMAN	Homo sapiens (Human)
Lucidumoside D_qt	O60911	CATL2_HUMAN	Homo sapiens (Human)
Lucidumoside D_qt	P49841	GSK3B_HUMAN	Homo sapiens (Human)
Lucidumoside D_qt	P00746	CFAD_HUMAN	Homo sapiens (Human)
Lucidumoside D_qt	Q08499	PDE4D_HUMAN	Homo sapiens (Human)
Lucidumoside D_qt	P02774	VTDB_HUMAN	Homo sapiens (Human)
Lucidumoside D_qt	P45452	MMP13_HUMAN	Homo sapiens (Human)
Lucidumoside D_qt	P02743	SAMP_HUMAN	Homo sapiens (Human)
Lucidumoside D_qt	P12931	SRC_HUMAN	Homo sapiens (Human)
Lucidumoside D_qt	P23141	EST1_HUMAN	Homo sapiens (Human)
Lucidumoside D_qt	P16152	CBR1_HUMAN	Homo sapiens (Human)
Lucidumoside D_qt	O76074	PDE5A_HUMAN	Homo sapiens (Human)
Lucidumoside D_qt	Q03181	PPARD_HUMAN	Homo sapiens (Human)
Lucidumoside D_qt	P36897	TGFR1_HUMAN	Homo sapiens (Human)
Lucidumoside D_qt	O14965	AURKA_HUMAN	Homo sapiens (Human)
Lucidumoside D_qt	P14174	MIF_HUMAN	Homo sapiens (Human)
Lucidumoside D_qt	P29474	NOS3_HUMAN	Homo sapiens (Human)
Lucidumoside D_qt	P07900	HS90A_HUMAN	Homo sapiens (Human)
Lucidumoside D_qt	Q07343	PDE4B_HUMAN	Homo sapiens (Human)
Lucidumoside D_qt	P35398	RORA_HUMAN	Homo sapiens (Human)
Lucidumoside D_qt	P01709	LV208_HUMAN	Homo sapiens (Human)

Lucidumoside D_qt	Q13231	CHIT1_HUMAN	Homo sapiens (Human)
Lucidumoside D_qt	P04278	SHBG_HUMAN	Homo sapiens (Human)
Lucidumoside D_qt	P42330	AK1C3_HUMAN	Homo sapiens (Human)
Lucidumoside D_qt	P47929	LEG7_HUMAN	Homo sapiens (Human)
Lucidumoside D_qt	P56817	BACE1_HUMAN	Homo sapiens (Human)
Lucidumoside D_qt	P08235	MCR_HUMAN	Homo sapiens (Human)
Lucidumoside D_qt	Q13370	PDE3B_HUMAN	Homo sapiens (Human)
Lucidumoside D_qt	P50579	MAP2_HUMAN	Homo sapiens (Human)
Lucidumoside D_qt	Q16539	MK14_HUMAN	Homo sapiens (Human)
Lucidumoside D_qt	O15382	BCAT2_HUMAN	Homo sapiens (Human)
Lucidumoside D_qt	P35968	VGFR2_HUMAN	Homo sapiens (Human)
Lucidumoside D_qt	P00390	GSHR_HUMAN	Homo sapiens (Human)
Lucidumoside D_qt	P84077	ARF1_HUMAN	Homo sapiens (Human)
Lucidumoside D_qt	P17707	DCAM_HUMAN	Homo sapiens (Human)
Lucidumoside D_qt	P53350	PLK1_HUMAN	Homo sapiens (Human)
Lucidumoside D_qt	P04818	TYSY_HUMAN	Homo sapiens (Human)
Lucidumoside D_qt	O15530	PDPK1_HUMAN	Homo sapiens (Human)
Lucidumoside D_qt	Q02127	PYRD_HUMAN	Homo sapiens (Human)
Lucidumoside D_qt	P49354	FNTA_HUMAN	Homo sapiens (Human)
Lucidumoside D_qt	P11586	C1TC_HUMAN	Homo sapiens (Human)
Lucidumoside D_qt	P06737	PYGL_HUMAN	Homo sapiens (Human)
Lucidumoside D_qt	P00374	DYR_HUMAN	Homo sapiens (Human)
Lucidumoside D_qt	P09960	LKHA4_HUMAN	Homo sapiens (Human)
Lucidumoside D_qt	P19793	RXRA_HUMAN	Homo sapiens (Human)
Lucidumoside D_qt	P15090	FABP4_HUMAN	Homo sapiens (Human)
Lucidumoside D_qt	P14061	DHB1_HUMAN	Homo sapiens (Human)
Lucidumoside D_qt	P00742	FA10_HUMAN	Homo sapiens (Human)
Lucidumoside D_qt	P22894	MMP8_HUMAN	Homo sapiens (Human)
Lucidumoside D_qt	P28845	DHI1_HUMAN	Homo sapiens (Human)
Lucidumoside D_qt	P78536	ADA17_HUMAN	Homo sapiens (Human)
Lucidumoside D_qt	P53355	DAPK1_HUMAN	Homo sapiens (Human)
Lucidumoside D_qt	P08069	IGF1R_HUMAN	Homo sapiens (Human)
Lucidumoside D_qt	P00439	PH4H_HUMAN	Homo sapiens (Human)
Lucidumoside D_qt	P31749	AKT1_HUMAN	Homo sapiens (Human)
Lucidumoside D_qt	Q06520	ST2A1_HUMAN	Homo sapiens (Human)
Lucidumoside D_qt	P27487	DPP4_HUMAN	Homo sapiens (Human)
Lucidumoside D_qt	P15056	BRAF_HUMAN	Homo sapiens (Human)
Lucidumoside D_qt	P43235	CATK_HUMAN	Homo sapiens (Human)
Lucidumoside D_qt	P09874	PARP1_HUMAN	Homo sapiens (Human)
Lucidumoside D_qt	Q9NVS9	PNPO_HUMAN	Homo sapiens (Human)
Lucidumoside D_qt	P39900	MMP12_HUMAN	Homo sapiens (Human)
Lucidumoside D_qt	Q00796	DHSO_HUMAN	Homo sapiens (Human)
Lucidumoside D_qt	P78368	KC1G2_HUMAN	Homo sapiens (Human)
Lucidumoside D_qt	P18031	PTN1_HUMAN	Homo sapiens (Human)
Lucidumoside D_qt	P11712	CP2C9_HUMAN	Homo sapiens (Human)
Lucidumoside D_qt	Q9BY41	HDAC8_HUMAN	Homo sapiens (Human)
Lucidumoside D_qt	P06239	LCK_HUMAN	Homo sapiens (Human)
Lucidumoside D_qt	P62508	ERR3_HUMAN	Homo sapiens (Human)
Lucidumoside D_qt	P34896	GLYC_HUMAN	Homo sapiens (Human)
Lucidumoside D_qt	Q08881	ITK_HUMAN	Homo sapiens (Human)
Lucidumoside D_qt	P25311	ZA2G_HUMAN	Homo sapiens (Human)
Lucidumoside D_qt	P35558	PCKGC_HUMAN	Homo sapiens (Human)
Lucidumoside D_qt	P52333	JAK3_HUMAN	Homo sapiens (Human)
Specnuezhenide	P07339	CATD_HUMAN	Homo sapiens (Human)
Specnuezhenide	P25774	CATS_HUMAN	Homo sapiens (Human)
Specnuezhenide	P23946	CMA1_HUMAN	Homo sapiens (Human)
Specnuezhenide	P00918	CAH2_HUMAN	Homo sapiens (Human)
Specnuezhenide	P00491	PNPH_HUMAN	Homo sapiens (Human)

Specnuezhenide	P53582	MAP11_HUMAN	Homo sapiens (Human)
Specnuezhenide	P54760	EPHB4_HUMAN	Homo sapiens (Human)
Specnuezhenide	P04745	AMY1_HUMAN	Homo sapiens (Human)
Specnuezhenide	Q92731	ESR2_HUMAN	Homo sapiens (Human)
Specnuezhenide	P26196	DDX6_HUMAN	Homo sapiens (Human)
Specnuezhenide	P55055	NR1H2_HUMAN	Homo sapiens (Human)
Specnuezhenide	P08631	HCK_HUMAN	Homo sapiens (Human)
Specnuezhenide	P09211	GSTP1_HUMAN	Homo sapiens (Human)
Specnuezhenide	P04062	GLCM_HUMAN	Homo sapiens (Human)
Specnuezhenide	P62937	PPIA_HUMAN	Homo sapiens (Human)
Specnuezhenide	P11309	PIM1_HUMAN	Homo sapiens (Human)
Specnuezhenide	P56817	BACE1_HUMAN	Homo sapiens (Human)
Specnuezhenide	P00749	UROK_HUMAN	Homo sapiens (Human)
Specnuezhenide	Q9BZR6	RTN4R_HUMAN	Homo sapiens (Human)
Specnuezhenide	P62942	FKB1A_HUMAN	Homo sapiens (Human)
Specnuezhenide	Q9BW91	NUDT9_HUMAN	Homo sapiens (Human)
Specnuezhenide	Q07343	PDE4B_HUMAN	Homo sapiens (Human)
Specnuezhenide	P17342	ANPRC_HUMAN	Homo sapiens (Human)
Specnuezhenide	O14757	CHK1_HUMAN	Homo sapiens (Human)
Specnuezhenide	P37173	TGFR2_HUMAN	Homo sapiens (Human)
Specnuezhenide	P06276	CHLE_HUMAN	Homo sapiens (Human)
Specnuezhenide	P00915	CAH1_HUMAN	Homo sapiens (Human)
Specnuezhenide	O14965	AURKA_HUMAN	Homo sapiens (Human)
Specnuezhenide	P20248	CCNA2_HUMAN	Homo sapiens (Human)
Specnuezhenide	Q12884	SEPR_HUMAN	Homo sapiens (Human)
Specnuezhenide	P07900	HS90A_HUMAN	Homo sapiens (Human)
Specnuezhenide	P45983	MK08_HUMAN	Homo sapiens (Human)
Specnuezhenide	Q9NP99	TREM1_HUMAN	Homo sapiens (Human)
Specnuezhenide	P00533	EGFR_HUMAN	Homo sapiens (Human)
Specnuezhenide	P29218	IMPA1_HUMAN	Homo sapiens (Human)
Specnuezhenide	Q16769	QPCT_HUMAN	Homo sapiens (Human)
Specnuezhenide	Q05315	LEG10_HUMAN	Homo sapiens (Human)
Specnuezhenide	O43570	CAH12_HUMAN	Homo sapiens (Human)
Specnuezhenide	P24941	CDK2_HUMAN	Homo sapiens (Human)
Specnuezhenide	P06401	PRGR_HUMAN	Homo sapiens (Human)
Specnuezhenide	P15121	ALDR_HUMAN	Homo sapiens (Human)
Specnuezhenide	P03950	ANGI_HUMAN	Homo sapiens (Human)
Specnuezhenide	Q13126	MTAP_HUMAN	Homo sapiens (Human)
Specnuezhenide	P17707	DCAM_HUMAN	Homo sapiens (Human)
Specnuezhenide	Q13231	CHIT1_HUMAN	Homo sapiens (Human)
Specnuezhenide	P47929	LEG7_HUMAN	Homo sapiens (Human)
Specnuezhenide	P00736	C1R_HUMAN	Homo sapiens (Human)
Specnuezhenide	P42574	CASP3_HUMAN	Homo sapiens (Human)
Specnuezhenide	P02766	TTHY_HUMAN	Homo sapiens (Human)
Specnuezhenide	P18075	BMP7_HUMAN	Homo sapiens (Human)
Specnuezhenide	P11142	HSP7C_HUMAN	Homo sapiens (Human)
Specnuezhenide	P54577	SYYC_HUMAN	Homo sapiens (Human)
Specnuezhenide	P48449	ERG7_HUMAN	Homo sapiens (Human)
Specnuezhenide	P19367	HXK1_HUMAN	Homo sapiens (Human)
Specnuezhenide	P62508	ERR3_HUMAN	Homo sapiens (Human)
Specnuezhenide	Q00796	DHSO_HUMAN	Homo sapiens (Human)
Specnuezhenide	P37231	PPARG_HUMAN	Homo sapiens (Human)
Specnuezhenide	P53355	DAPK1_HUMAN	Homo sapiens (Human)
Specnuezhenide	P06737	PYGL_HUMAN	Homo sapiens (Human)
Specnuezhenide	O60911	CATL2_HUMAN	Homo sapiens (Human)
Specnuezhenide	P00746	CFAD_HUMAN	Homo sapiens (Human)
Specnuezhenide	Q08499	PDE4D_HUMAN	Homo sapiens (Human)
Specnuezhenide	Q9NVS9	PNPO_HUMAN	Homo sapiens (Human)

Specnuezhenide	P25311	ZA2G_HUMAN	Homo sapiens (Human)
Specnuezhenide	P52732	KIF11_HUMAN	Homo sapiens (Human)
Specnuezhenide	O76074	PDE5A_HUMAN	Homo sapiens (Human)
Specnuezhenide	P12931	SRC_HUMAN	Homo sapiens (Human)
Specnuezhenide	P00734	THRB_HUMAN	Homo sapiens (Human)
Specnuezhenide	P07858	CATB_HUMAN	Homo sapiens (Human)
Specnuezhenide	P53350	PLK1_HUMAN	Homo sapiens (Human)
Specnuezhenide	P36897	TGFR1_HUMAN	Homo sapiens (Human)
Specnuezhenide	P00325	ADH1B_HUMAN	Homo sapiens (Human)
Specnuezhenide	Q07912	ACK1_HUMAN	Homo sapiens (Human)
Specnuezhenide	P00742	FA10_HUMAN	Homo sapiens (Human)
Specnuezhenide	Q16539	MK14_HUMAN	Homo sapiens (Human)
Specnuezhenide	P00390	GSHR_HUMAN	Homo sapiens (Human)
Specnuezhenide	P03372	ESR1_HUMAN	Homo sapiens (Human)
Specnuezhenide	P19971	TYPH_HUMAN	Homo sapiens (Human)
Specnuezhenide	P78536	ADA17_HUMAN	Homo sapiens (Human)
Specnuezhenide	P01709	LV208_HUMAN	Homo sapiens (Human)
Specnuezhenide	P14174	MIF_HUMAN	Homo sapiens (Human)
Specnuezhenide	P55210	CASP7_HUMAN	Homo sapiens (Human)
Specnuezhenide	Q06520	ST2A1_HUMAN	Homo sapiens (Human)
Specnuezhenide	Q13370	PDE3B_HUMAN	Homo sapiens (Human)
Specnuezhenide	P04818	TYSY_HUMAN	Homo sapiens (Human)
Specnuezhenide	P02743	SAMP_HUMAN	Homo sapiens (Human)
Specnuezhenide	P39900	MMP12_HUMAN	Homo sapiens (Human)
Specnuezhenide	P00751	CFAB_HUMAN	Homo sapiens (Human)
Specnuezhenide	P00374	DYR_HUMAN	Homo sapiens (Human)
Specnuezhenide	P04278	SHBG_HUMAN	Homo sapiens (Human)
Specnuezhenide	P78368	KC1G2_HUMAN	Homo sapiens (Human)
Specnuezhenide	P29474	NOS3_HUMAN	Homo sapiens (Human)
Specnuezhenide	O15382	BCAT2_HUMAN	Homo sapiens (Human)
Specnuezhenide	P08254	MMP3_HUMAN	Homo sapiens (Human)
Specnuezhenide	P00439	PH4H_HUMAN	Homo sapiens (Human)
Specnuezhenide	P49789	FHIT_HUMAN	Homo sapiens (Human)
Specnuezhenide	P49841	GSK3B_HUMAN	Homo sapiens (Human)
Specnuezhenide	P45452	MMP13_HUMAN	Homo sapiens (Human)
Specnuezhenide	P07686	HEXB_HUMAN	Homo sapiens (Human)
Specnuezhenide	P10275	ANDR_HUMAN	Homo sapiens (Human)
Specnuezhenide	P43405	KSYK_HUMAN	Homo sapiens (Human)
Specnuezhenide	P53779	MK10_HUMAN	Homo sapiens (Human)
Specnuezhenide	P08758	ANXA5_HUMAN	Homo sapiens (Human)
Specnuezhenide	P43235	CATK_HUMAN	Homo sapiens (Human)
Specnuezhenide	P16083	NQO2_HUMAN	Homo sapiens (Human)
Specnuezhenide	Q02127	PYRD_HUMAN	Homo sapiens (Human)
Specnuezhenide	P84077	ARF1_HUMAN	Homo sapiens (Human)
Specnuezhenide	O15530	PDPK1_HUMAN	Homo sapiens (Human)
Specnuezhenide	Q96AZ6	ISG20_HUMAN	Homo sapiens (Human)
Specnuezhenide	P09237	MMP7_HUMAN	Homo sapiens (Human)
Specnuezhenide	P11586	C1TC_HUMAN	Homo sapiens (Human)
Specnuezhenide	P31749	AKT1_HUMAN	Homo sapiens (Human)
Specnuezhenide	P12268	IMDH2_HUMAN	Homo sapiens (Human)
Specnuezhenide	P19793	RXRA_HUMAN	Homo sapiens (Human)
Specnuezhenide	Q99933	BAG1_HUMAN	Homo sapiens (Human)
Specnuezhenide	P51570	GALK1_HUMAN	Homo sapiens (Human)
Specnuezhenide	Q96T66	NMNA3_HUMAN	Homo sapiens (Human)
Specnuezhenide	P68400	CSK21_HUMAN	Homo sapiens (Human)
Specnuezhenide	P18031	PTN1_HUMAN	Homo sapiens (Human)
Specnuezhenide	P22894	MMP8_HUMAN	Homo sapiens (Human)
Specnuezhenide	P49354	FNTA_HUMAN	Homo sapiens (Human)

Specnuezhenide	P23526	SAHH_HUMAN	Homo sapiens (Human)
Specnuezhenide	P62166	NCS1_HUMAN	Homo sapiens (Human)
Specnuezhenide	P08069	IGF1R_HUMAN	Homo sapiens (Human)
Specnuezhenide	Q9P2W7	B3GA1_HUMAN	Homo sapiens (Human)
Specnuezhenide	P04179	SODM_HUMAN	Homo sapiens (Human)
Specnuezhenide	P28845	DHI1_HUMAN	Homo sapiens (Human)
Specnuezhenide	P23141	EST1_HUMAN	Homo sapiens (Human)
Specnuezhenide	P27487	DPP4_HUMAN	Homo sapiens (Human)
Specnuezhenide	P05451	REG1A_HUMAN	Homo sapiens (Human)
Specnuezhenide	P35968	VGFR2_HUMAN	Homo sapiens (Human)
Specnuezhenide	P35558	PCKGC_HUMAN	Homo sapiens (Human)
Specnuezhenide	P43155	CACP_HUMAN	Homo sapiens (Human)
Specnuezhenide	Q07960	RHG01_HUMAN	Homo sapiens (Human)
Specnuezhenide	P04035	HMDH_HUMAN	Homo sapiens (Human)
Specnuezhenide	P04746	AMYP_HUMAN	Homo sapiens (Human)
Specnuezhenide	Q08188	TGM3_HUMAN	Homo sapiens (Human)
Specnuezhenide	P11362	FGFR1_HUMAN	Homo sapiens (Human)
Specnuezhenide	Q03181	PPARD_HUMAN	Homo sapiens (Human)
Specnuezhenide	Q15382	RHEB_HUMAN	Homo sapiens (Human)
Specnuezhenide	Q9BZX2	UCK2_HUMAN	Homo sapiens (Human)
Specnuezhenide	P50579	MAP2_HUMAN	Homo sapiens (Human)
Specnuezhenide	P27338	AOFB_HUMAN	Homo sapiens (Human)
Specnuezhenide	P60568	IL2_HUMAN	Homo sapiens (Human)
Oleanolic acid	P08842	STS_HUMAN	Homo sapiens (Human)
Oleanolic acid	P02768	ALBU_HUMAN	Homo sapiens (Human)
Oleanolic acid	P35968	VGFR2_HUMAN	Homo sapiens (Human)
Oleanolic acid	P53779	MK10_HUMAN	Homo sapiens (Human)
Oleanolic acid	P08254	MMP3_HUMAN	Homo sapiens (Human)
Oleanolic acid	P12643	BMP2_HUMAN	Homo sapiens (Human)
Oleanolic acid	P10828	THB_HUMAN	Homo sapiens (Human)
Oleanolic acid	P27338	AOFB_HUMAN	Homo sapiens (Human)
Oleanolic acid	P45452	MMP13_HUMAN	Homo sapiens (Human)
Oleanolic acid	P11309	PIM1_HUMAN	Homo sapiens (Human)
Oleanolic acid	P00918	CAH2_HUMAN	Homo sapiens (Human)
Oleanolic acid	P49137	MAPK2_HUMAN	Homo sapiens (Human)
Oleanolic acid	P02652	APOA2_HUMAN	Homo sapiens (Human)
Oleanolic acid	P52895	AK1C2_HUMAN	Homo sapiens (Human)
Oleanolic acid	Q8NBQ5	DHB11_HUMAN	Homo sapiens (Human)
Oleanolic acid	P62937	PPIA_HUMAN	Homo sapiens (Human)
Oleanolic acid	P00751	CFAB_HUMAN	Homo sapiens (Human)
Oleanolic acid	P52732	KIF11_HUMAN	Homo sapiens (Human)
Oleanolic acid	P06276	CHLE_HUMAN	Homo sapiens (Human)
Oleanolic acid	P00734	THRB_HUMAN	Homo sapiens (Human)
Oleanolic acid	P28482	MK01_HUMAN	Homo sapiens (Human)
Oleanolic acid	P02774	VTDB_HUMAN	Homo sapiens (Human)
Oleanolic acid	P20701	ITAL_HUMAN	Homo sapiens (Human)
Oleanolic acid	P55210	CASP7_HUMAN	Homo sapiens (Human)
Oleanolic acid	P02766	TTHY_HUMAN	Homo sapiens (Human)
Oleanolic acid	P56817	BACE1_HUMAN	Homo sapiens (Human)
Oleanolic acid	Q06520	ST2A1_HUMAN	Homo sapiens (Human)
Oleanolic acid	P00915	CAH1_HUMAN	Homo sapiens (Human)
Oleanolic acid	Q02127	PYRD_HUMAN	Homo sapiens (Human)
Oleanolic acid	P09211	GSTP1_HUMAN	Homo sapiens (Human)
Oleanolic acid	P06401	PRGR_HUMAN	Homo sapiens (Human)
Oleanolic acid	P35398	RORA_HUMAN	Homo sapiens (Human)
Oleanolic acid	P09874	PARP1_HUMAN	Homo sapiens (Human)
Oleanolic acid	P10275	ANDR_HUMAN	Homo sapiens (Human)
Oleanolic acid	P14061	DHB1_HUMAN	Homo sapiens (Human)

Oleanolic acid	P08235	MCR_HUMAN	Homo sapiens (Human)
Oleanolic acid	P28845	DHI1_HUMAN	Homo sapiens (Human)
Oleanolic acid	P53041	PPP5_HUMAN	Homo sapiens (Human)
Oleanolic acid	P37231	PPARG_HUMAN	Homo sapiens (Human)
Oleanolic acid	P12931	SRC_HUMAN	Homo sapiens (Human)
Oleanolic acid	P20248	CCNA2_HUMAN	Homo sapiens (Human)
Oleanolic acid	O43617	TPPC3_HUMAN	Homo sapiens (Human)
Oleanolic acid	P29474	NOS3_HUMAN	Homo sapiens (Human)
Oleanolic acid	O15530	PDPK1_HUMAN	Homo sapiens (Human)
Oleanolic acid	P45983	MK08_HUMAN	Homo sapiens (Human)
Oleanolic acid	P24941	CDK2_HUMAN	Homo sapiens (Human)
Oleanolic acid	P03372	ESR1_HUMAN	Homo sapiens (Human)
Oleanolic acid	P78536	ADA17_HUMAN	Homo sapiens (Human)
Oleanolic acid	O14965	AURKA_HUMAN	Homo sapiens (Human)
Oleanolic acid	P00746	CFAD_HUMAN	Homo sapiens (Human)
Oleanolic acid	P00326	ADH1G_HUMAN	Homo sapiens (Human)
Oleanolic acid	O14757	CHK1_HUMAN	Homo sapiens (Human)
Oleanolic acid	P49354	FNTA_HUMAN	Homo sapiens (Human)
Oleanolic acid	P00325	ADH1B_HUMAN	Homo sapiens (Human)
Oleanolic acid	Q16539	MK14_HUMAN	Homo sapiens (Human)
Oleanolic acid	Q07343	PDE4B_HUMAN	Homo sapiens (Human)
Oleanolic acid	O76054	S14L2_HUMAN	Homo sapiens (Human)
Oleanolic acid	P11766	ADHX_HUMAN	Homo sapiens (Human)
Oleanolic acid	Q08499	PDE4D_HUMAN	Homo sapiens (Human)
Oleanolic acid	O15496	PA2GX_HUMAN	Homo sapiens (Human)
Oleanolic acid	P04278	SHBG_HUMAN	Homo sapiens (Human)
Oleanolic acid	P62508	ERR3_HUMAN	Homo sapiens (Human)
Oleanolic acid	Q06124	PTN11_HUMAN	Homo sapiens (Human)
Oleanolic acid	Q16828	DUS6_HUMAN	Homo sapiens (Human)
Oleanolic acid	P08758	ANXA5_HUMAN	Homo sapiens (Human)
Oleanolic acid	P23141	EST1_HUMAN	Homo sapiens (Human)
Oleanolic acid	Q04828	AK1C1_HUMAN	Homo sapiens (Human)
Oleanolic acid	P54760	EPHB4_HUMAN	Homo sapiens (Human)
Oleanolic acid	P63316	TNNC1_HUMAN	Homo sapiens (Human)
Oleanolic acid	P00797	RENI_HUMAN	Homo sapiens (Human)
Oleanolic acid	P00533	EGFR_HUMAN	Homo sapiens (Human)
Oleanolic acid	Q96RI1	NR1H4_HUMAN	Homo sapiens (Human)
Oleanolic acid	P55055	NR1H2_HUMAN	Homo sapiens (Human)
Oleanolic acid	Q9BY41	HDAC8_HUMAN	Homo sapiens (Human)
Oleanolic acid	P07900	HS90A_HUMAN	Homo sapiens (Human)
Oleanolic acid	P27487	DPP4_HUMAN	Homo sapiens (Human)
Oleanolic acid	P15056	BRAF_HUMAN	Homo sapiens (Human)
Oleanolic acid	P15121	ALDR_HUMAN	Homo sapiens (Human)
Oleanolic acid	Q00796	DHSO_HUMAN	Homo sapiens (Human)
Oleanolic acid	P00390	GSHR_HUMAN	Homo sapiens (Human)
Oleanolic acid	P0DP23	CALM1_HUMAN	Homo sapiens (Human)
Oleanolic acid	P0DP24	CALM2_HUMAN	Homo sapiens (Human)
Oleanolic acid	P0DP25	CALM3_HUMAN	Homo sapiens (Human)
Oleanolic acid	P06239	LCK_HUMAN	Homo sapiens (Human)
Oleanolic acid	P08581	MET_HUMAN	Homo sapiens (Human)
Oleanolic acid	O60760	HPGDS_HUMAN	Homo sapiens (Human)
Oleanolic acid	Q13231	CHIT1_HUMAN	Homo sapiens (Human)
Oleanolic acid	P49763	PLGF_HUMAN	Homo sapiens (Human)
Oleanolic acid	P08263	GSTA1_HUMAN	Homo sapiens (Human)
Oleanolic acid	O15382	BCAT2_HUMAN	Homo sapiens (Human)
Oleanolic acid	P18031	PTN1_HUMAN	Homo sapiens (Human)
Oleanolic acid	P06746	DPOLB_HUMAN	Homo sapiens (Human)
Oleanolic acid	O60218	AK1BA_HUMAN	Homo sapiens (Human)

Oleanolic acid	P51449	RORG_HUMAN	Homo sapiens (Human)
Oleanolic acid	P10586	PTPRF_HUMAN	Homo sapiens (Human)
Oleanolic acid	P17706	PTN2_HUMAN	Homo sapiens (Human)
Oleanolic acid	P24666	PPAC_HUMAN	Homo sapiens (Human)
Oleanolic acid	P30305	MPIP2_HUMAN	Homo sapiens (Human)
Salidroside	P00918	CAH2_HUMAN	Homo sapiens (Human)
Salidroside	P56817	BACE1_HUMAN	Homo sapiens (Human)
Salidroside	Q92731	ESR2_HUMAN	Homo sapiens (Human)
Salidroside	P07339	CATD_HUMAN	Homo sapiens (Human)
Salidroside	Q9BW91	NUDT9_HUMAN	Homo sapiens (Human)
Salidroside	P25774	CATS_HUMAN	Homo sapiens (Human)
Salidroside	P54760	EPHB4_HUMAN	Homo sapiens (Human)
Salidroside	Q13126	MTAP_HUMAN	Homo sapiens (Human)
Salidroside	P53582	MAP11_HUMAN	Homo sapiens (Human)
Salidroside	P04062	GLCM_HUMAN	Homo sapiens (Human)
Salidroside	P37173	TGFR2_HUMAN	Homo sapiens (Human)
Salidroside	P11309	PIM1_HUMAN	Homo sapiens (Human)
Salidroside	P08631	HCK_HUMAN	Homo sapiens (Human)
Salidroside	P20248	CCNA2_HUMAN	Homo sapiens (Human)
Salidroside	P62942	FKB1A_HUMAN	Homo sapiens (Human)
Salidroside	P04745	AMY1_HUMAN	Homo sapiens (Human)
Salidroside	P62937	PPIA_HUMAN	Homo sapiens (Human)
Salidroside	O14757	CHK1_HUMAN	Homo sapiens (Human)
Salidroside	O14965	AURKA_HUMAN	Homo sapiens (Human)
Salidroside	P28482	MK01_HUMAN	Homo sapiens (Human)
Salidroside	P09211	GSTP1_HUMAN	Homo sapiens (Human)
Salidroside	P15121	ALDR_HUMAN	Homo sapiens (Human)
Salidroside	P03372	ESR1_HUMAN	Homo sapiens (Human)
Salidroside	P17342	ANPRC_HUMAN	Homo sapiens (Human)
Salidroside	Q12884	SEPR_HUMAN	Homo sapiens (Human)
Salidroside	P00915	CAH1_HUMAN	Homo sapiens (Human)
Salidroside	P24941	CDK2_HUMAN	Homo sapiens (Human)
Salidroside	P06276	CHLE_HUMAN	Homo sapiens (Human)
Salidroside	P29218	IMPA1_HUMAN	Homo sapiens (Human)
Salidroside	Q07343	PDE4B_HUMAN	Homo sapiens (Human)
Salidroside	P11142	HSP7C_HUMAN	Homo sapiens (Human)
Salidroside	Q05315	LEG10_HUMAN	Homo sapiens (Human)
Salidroside	O43570	CAH12_HUMAN	Homo sapiens (Human)
Salidroside	P07900	HS90A_HUMAN	Homo sapiens (Human)
Salidroside	P55055	NR1H2_HUMAN	Homo sapiens (Human)
Salidroside	P06401	PRGR_HUMAN	Homo sapiens (Human)
Salidroside	P23946	CMA1_HUMAN	Homo sapiens (Human)
Salidroside	P19367	HXK1_HUMAN	Homo sapiens (Human)
Salidroside	P26196	DDX6_HUMAN	Homo sapiens (Human)
Salidroside	O60911	CATL2_HUMAN	Homo sapiens (Human)
Salidroside	P42574	CASP3_HUMAN	Homo sapiens (Human)
Salidroside	P00491	PNPH_HUMAN	Homo sapiens (Human)
Salidroside	Q16769	QPCT_HUMAN	Homo sapiens (Human)
Salidroside	P00533	EGFR_HUMAN	Homo sapiens (Human)
Salidroside	Q15078	CD5R1_HUMAN	Homo sapiens (Human)
Salidroside	P54577	SYYC_HUMAN	Homo sapiens (Human)
Salidroside	P51570	GALK1_HUMAN	Homo sapiens (Human)
Salidroside	P53355	DAPK1_HUMAN	Homo sapiens (Human)
Salidroside	P28845	DHI1_HUMAN	Homo sapiens (Human)
Salidroside	P02766	TTHY_HUMAN	Homo sapiens (Human)
Salidroside	P52732	KIF11_HUMAN	Homo sapiens (Human)
Salidroside	P00734	THRB_HUMAN	Homo sapiens (Human)
Salidroside	P00746	CFAD_HUMAN	Homo sapiens (Human)

Salidroside	Q16539	MK14_HUMAN	Homo sapiens (Human)
Salidroside	P14174	MIF_HUMAN	Homo sapiens (Human)
Salidroside	P12931	SRC_HUMAN	Homo sapiens (Human)
Salidroside	P29474	NOS3_HUMAN	Homo sapiens (Human)
Salidroside	P17707	DCAM_HUMAN	Homo sapiens (Human)
Salidroside	Q07912	ACK1_HUMAN	Homo sapiens (Human)
Salidroside	P10275	ANDR_HUMAN	Homo sapiens (Human)
Salidroside	Q9NVS9	PNPO_HUMAN	Homo sapiens (Human)
Salidroside	P37231	PPARG_HUMAN	Homo sapiens (Human)
Salidroside	P00325	ADH1B_HUMAN	Homo sapiens (Human)
Salidroside	P03950	ANGI_HUMAN	Homo sapiens (Human)
Salidroside	P62508	ERR3_HUMAN	Homo sapiens (Human)
Salidroside	O76074	PDE5A_HUMAN	Homo sapiens (Human)
Salidroside	P06737	PYGL_HUMAN	Homo sapiens (Human)
Salidroside	P78536	ADA17_HUMAN	Homo sapiens (Human)
Salidroside	P07858	CATB_HUMAN	Homo sapiens (Human)
Salidroside	P00374	DYR_HUMAN	Homo sapiens (Human)
Salidroside	Q08499	PDE4D_HUMAN	Homo sapiens (Human)
Salidroside	P31749	AKT1_HUMAN	Homo sapiens (Human)
Salidroside	P08758	ANXA5_HUMAN	Homo sapiens (Human)
Salidroside	P36897	TGFR1_HUMAN	Homo sapiens (Human)
Salidroside	Q13370	PDE3B_HUMAN	Homo sapiens (Human)
Salidroside	P04818	TYSY_HUMAN	Homo sapiens (Human)
Salidroside	P00797	RENI_HUMAN	Homo sapiens (Human)
Salidroside	P55210	CASP7_HUMAN	Homo sapiens (Human)
Salidroside	P04278	SHBG_HUMAN	Homo sapiens (Human)
Salidroside	P04035	HMDH_HUMAN	Homo sapiens (Human)
Salidroside	P45452	MMP13_HUMAN	Homo sapiens (Human)
Salidroside	P18075	BMP7_HUMAN	Homo sapiens (Human)
Salidroside	P68400	CSK21_HUMAN	Homo sapiens (Human)
Salidroside	P00751	CFAB_HUMAN	Homo sapiens (Human)
Salidroside	P53779	MK10_HUMAN	Homo sapiens (Human)
Salidroside	P00390	GSHR_HUMAN	Homo sapiens (Human)
Salidroside	P48449	ERG7_HUMAN	Homo sapiens (Human)
Salidroside	P06239	LCK_HUMAN	Homo sapiens (Human)
Salidroside	P78368	KC1G2_HUMAN	Homo sapiens (Human)
Salidroside	P27487	DPP4_HUMAN	Homo sapiens (Human)
Salidroside	P00439	PH4H_HUMAN	Homo sapiens (Human)
Salidroside	P35968	VGFR2_HUMAN	Homo sapiens (Human)
Salidroside	P16083	NQO2_HUMAN	Homo sapiens (Human)
Salidroside	P07686	HEXB_HUMAN	Homo sapiens (Human)
Salidroside	O15530	PDPK1_HUMAN	Homo sapiens (Human)
Salidroside	P01709	LV208_HUMAN	Homo sapiens (Human)
Salidroside	P19971	TYPH_HUMAN	Homo sapiens (Human)
Salidroside	P25311	ZA2G_HUMAN	Homo sapiens (Human)
Salidroside	P53350	PLK1_HUMAN	Homo sapiens (Human)
Salidroside	P43155	CACP_HUMAN	Homo sapiens (Human)
Salidroside	Q06520	ST2A1_HUMAN	Homo sapiens (Human)
Salidroside	P43235	CATK_HUMAN	Homo sapiens (Human)
Salidroside	Q99933	BAG1_HUMAN	Homo sapiens (Human)
Salidroside	P00742	FA10_HUMAN	Homo sapiens (Human)
Salidroside	Q02127	PYRD_HUMAN	Homo sapiens (Human)
Salidroside	Q08881	ITK_HUMAN	Homo sapiens (Human)
Salidroside	P45983	MK08_HUMAN	Homo sapiens (Human)
Salidroside	P08254	MMP3_HUMAN	Homo sapiens (Human)
Salidroside	P11362	FGFR1_HUMAN	Homo sapiens (Human)
Salidroside	P00736	C1R_HUMAN	Homo sapiens (Human)
Salidroside	Q9P2W7	B3GA1_HUMAN	Homo sapiens (Human)

Salidroside	P32320	CDD_HUMAN	Homo sapiens (Human)
Salidroside	P35558	PCKGC_HUMAN	Homo sapiens (Human)
Salidroside	P22894	MMP8_HUMAN	Homo sapiens (Human)
Salidroside	P05451	REG1A_HUMAN	Homo sapiens (Human)
Salidroside	P49841	GSK3B_HUMAN	Homo sapiens (Human)
Salidroside	P47929	LEG7_HUMAN	Homo sapiens (Human)
Salidroside	P11586	C1TC_HUMAN	Homo sapiens (Human)
Salidroside	Q00534	CDK6_HUMAN	Homo sapiens (Human)
Salidroside	P23526	SAHH_HUMAN	Homo sapiens (Human)
Salidroside	P18031	PTN1_HUMAN	Homo sapiens (Human)
Salidroside	P08069	IGF1R_HUMAN	Homo sapiens (Human)
Salidroside	P08235	MCR_HUMAN	Homo sapiens (Human)
Salidroside	P04179	SODM_HUMAN	Homo sapiens (Human)
Salidroside	P34913	HYES_HUMAN	Homo sapiens (Human)
Salidroside	P16442	BGAT_HUMAN	Homo sapiens (Human)
Salidroside	P15309	PPAP_HUMAN	Homo sapiens (Human)
Salidroside	P27707	DCK_HUMAN	Homo sapiens (Human)
(20S)-24-ene-3β,20-diol-3-acetat	P27338	AOFB_HUMAN	Homo sapiens (Human)
(20S)-24-ene-3β,20-diol-3-acetat	P53779	MK10_HUMAN	Homo sapiens (Human)
(20S)-24-ene-3β,20-diol-3-acetat	P12643	BMP2_HUMAN	Homo sapiens (Human)
(20S)-24-ene-3β,20-diol-3-acetat	P00918	CAH2_HUMAN	Homo sapiens (Human)
(20S)-24-ene-3β,20-diol-3-acetat	Q16539	MK14_HUMAN	Homo sapiens (Human)
(20S)-24-ene-3β,20-diol-3-acetat	P08254	MMP3_HUMAN	Homo sapiens (Human)
(20S)-24-ene-3β,20-diol-3-acetat	P49137	MAPK2_HUMAN	Homo sapiens (Human)
(20S)-24-ene-3β,20-diol-3-acetat	P52895	AK1C2_HUMAN	Homo sapiens (Human)
(20S)-24-ene-3β,20-diol-3-acetat	P00751	CFAB_HUMAN	Homo sapiens (Human)
(20S)-24-ene-3β,20-diol-3-acetat	P08842	STS_HUMAN	Homo sapiens (Human)
(20S)-24-ene-3β,20-diol-3-acetat	P28482	MK01_HUMAN	Homo sapiens (Human)
(20S)-24-ene-3β,20-diol-3-acetat	P02766	TTHY_HUMAN	Homo sapiens (Human)
(20S)-24-ene-3β,20-diol-3-acetat	P62937	PPIA_HUMAN	Homo sapiens (Human)
(20S)-24-ene-3β,20-diol-3-acetat	P45452	MMP13_HUMAN	Homo sapiens (Human)
(20S)-24-ene-3β,20-diol-3-acetat	Q92731	ESR2_HUMAN	Homo sapiens (Human)
(20S)-24-ene-3β,20-diol-3-acetat	P02652	APOA2_HUMAN	Homo sapiens (Human)
(20S)-24-ene-3β,20-diol-3-acetat	P02768	ALBU_HUMAN	Homo sapiens (Human)
(20S)-24-ene-3β,20-diol-3-acetat	P52732	KIF11_HUMAN	Homo sapiens (Human)
(20S)-24-ene-3β,20-diol-3-acetat	P11309	PIM1_HUMAN	Homo sapiens (Human)
(20S)-24-ene-3β,20-diol-3-acetat	P45983	MK08_HUMAN	Homo sapiens (Human)
(20S)-24-ene-3β,20-diol-3-acetat	P00734	THRB_HUMAN	Homo sapiens (Human)
(20S)-24-ene-3β,20-diol-3-acetat	P35968	VGFR2_HUMAN	Homo sapiens (Human)
(20S)-24-ene-3β,20-diol-3-acetat	P10275	ANDR_HUMAN	Homo sapiens (Human)
(20S)-24-ene-3β,20-diol-3-acetat	Q8NBQ5	DHB11_HUMAN	Homo sapiens (Human)
(20S)-24-ene-3β,20-diol-3-acetat	P02774	VTDB_HUMAN	Homo sapiens (Human)
(20S)-24-ene-3β,20-diol-3-acetat	P06276	CHLE_HUMAN	Homo sapiens (Human)
(20S)-24-ene-3β,20-diol-3-acetat	P06401	PRGR_HUMAN	Homo sapiens (Human)
(20S)-24-ene-3β,20-diol-3-acetat	P23141	EST1_HUMAN	Homo sapiens (Human)
(20S)-24-ene-3β,20-diol-3-acetat	P03372	ESR1_HUMAN	Homo sapiens (Human)
(20S)-24-ene-3β,20-diol-3-acetat	P15121	ALDR_HUMAN	Homo sapiens (Human)
(20S)-24-ene-3β,20-diol-3-acetat	P10828	THB_HUMAN	Homo sapiens (Human)
(20S)-24-ene-3β,20-diol-3-acetat	P20248	CCNA2_HUMAN	Homo sapiens (Human)
(20S)-24-ene-3β,20-diol-3-acetat	P11511	CP19A_HUMAN	Homo sapiens (Human)
(20S)-24-ene-3β,20-diol-3-acetat	P63316	TNNC1_HUMAN	Homo sapiens (Human)
(20S)-24-ene-3β,20-diol-3-acetat	P55210	CASP7_HUMAN	Homo sapiens (Human)
(20S)-24-ene-3β,20-diol-3-acetat	Q06520	ST2A1_HUMAN	Homo sapiens (Human)
(20S)-24-ene-3β,20-diol-3-acetat	P24941	CDK2_HUMAN	Homo sapiens (Human)
(20S)-24-ene-3β,20-diol-3-acetat	Q02127	PYRD_HUMAN	Homo sapiens (Human)
(20S)-24-ene-3β,20-diol-3-acetat	O76074	PDE5A_HUMAN	Homo sapiens (Human)
(20S)-24-ene-3β,20-diol-3-acetat	P09211	GSTP1_HUMAN	Homo sapiens (Human)
(20S)-24-ene-3β,20-diol-3-acetat	P20701	ITAL_HUMAN	Homo sapiens (Human)

(20S)-24-ene-3β,20-diol-3-acetat	P54760	EPHB4_HUMAN	Homo sapiens (Human)
(20S)-24-ene-3β,20-diol-3-acetat	P08235	MCR_HUMAN	Homo sapiens (Human)
(20S)-24-ene-3β,20-diol-3-acetat	P35398	RORA_HUMAN	Homo sapiens (Human)
(20S)-24-ene-3β,20-diol-3-acetat	P78536	ADA17_HUMAN	Homo sapiens (Human)
(20S)-24-ene-3β,20-diol-3-acetat	P37231	PPARG_HUMAN	Homo sapiens (Human)
(20S)-24-ene-3β,20-diol-3-acetat	P08758	ANXA5_HUMAN	Homo sapiens (Human)
(20S)-24-ene-3β,20-diol-3-acetat	O14757	CHK1_HUMAN	Homo sapiens (Human)
(20S)-24-ene-3β,20-diol-3-acetat	P19793	RXRA_HUMAN	Homo sapiens (Human)
(20S)-24-ene-3β,20-diol-3-acetat	Q03181	PPARD_HUMAN	Homo sapiens (Human)
(20S)-24-ene-3β,20-diol-3-acetat	P14061	DHB1_HUMAN	Homo sapiens (Human)
(20S)-24-ene-3β,20-diol-3-acetat	Q96RI1	NR1H4_HUMAN	Homo sapiens (Human)
(20S)-24-ene-3β,20-diol-3-acetat	P04278	SHBG_HUMAN	Homo sapiens (Human)
(20S)-24-ene-3β,20-diol-3-acetat	P29474	NOS3_HUMAN	Homo sapiens (Human)
(20S)-24-ene-3β,20-diol-3-acetat	Q13126	MTAP_HUMAN	Homo sapiens (Human)
(20S)-24-ene-3β,20-diol-3-acetat	P18031	PTN1_HUMAN	Homo sapiens (Human)
(20S)-24-ene-3β,20-diol-3-acetat	P00742	FA10_HUMAN	Homo sapiens (Human)
(20S)-24-ene-3β,20-diol-3-acetat	Q07343	PDE4B_HUMAN	Homo sapiens (Human)
(20S)-24-ene-3β,20-diol-3-acetat	P53350	PLK1_HUMAN	Homo sapiens (Human)
(20S)-24-ene-3β,20-diol-3-acetat	P53041	PPP5_HUMAN	Homo sapiens (Human)
(20S)-24-ene-3β,20-diol-3-acetat	P50579	MAP2_HUMAN	Homo sapiens (Human)
(20S)-24-ene-3β,20-diol-3-acetat	O76054	S14L2_HUMAN	Homo sapiens (Human)
(20S)-24-ene-3β,20-diol-3-acetat	P12931	SRC_HUMAN	Homo sapiens (Human)
(20S)-24-ene-3β,20-diol-3-acetat	P00533	EGFR_HUMAN	Homo sapiens (Human)
(20S)-24-ene-3β,20-diol-3-acetat	P22894	MMP8_HUMAN	Homo sapiens (Human)
(20S)-24-ene-3β,20-diol-3-acetat	P56817	BACE1_HUMAN	Homo sapiens (Human)
(20S)-24-ene-3β,20-diol-3-acetat	P49354	FNTA_HUMAN	Homo sapiens (Human)
(20S)-24-ene-3β,20-diol-3-acetat	P15056	BRAF_HUMAN	Homo sapiens (Human)
(20S)-24-ene-3β,20-diol-3-acetat	P42574	CASP3_HUMAN	Homo sapiens (Human)
(20S)-24-ene-3β,20-diol-3-acetat	O15382	BCAT2_HUMAN	Homo sapiens (Human)
(20S)-24-ene-3β,20-diol-3-acetat	P11142	HSP7C_HUMAN	Homo sapiens (Human)
(20S)-24-ene-3β,20-diol-3-acetat	P00390	GSHR_HUMAN	Homo sapiens (Human)
(20S)-24-ene-3β,20-diol-3-acetat	P00325	ADH1B_HUMAN	Homo sapiens (Human)
(20S)-24-ene-3β,20-diol-3-acetat	P00374	DYR_HUMAN	Homo sapiens (Human)
(20S)-24-ene-3β,20-diol-3-acetat	P28845	DHI1_HUMAN	Homo sapiens (Human)
(20S)-24-ene-3β,20-diol-3-acetat	P42330	AK1C3_HUMAN	Homo sapiens (Human)
(20S)-24-ene-3β,20-diol-3-acetat	P04818	TYSY_HUMAN	Homo sapiens (Human)
(20S)-24-ene-3β,20-diol-3-acetat	P36897	TGFR1_HUMAN	Homo sapiens (Human)
(20S)-24-ene-3β,20-diol-3-acetat	P11362	FGFR1_HUMAN	Homo sapiens (Human)
(20S)-24-ene-3β,20-diol-3-acetat	P16083	NQO2_HUMAN	Homo sapiens (Human)
(20S)-24-ene-3β,20-diol-3-acetat	P27487	DPP4_HUMAN	Homo sapiens (Human)
(20S)-24-ene-3β,20-diol-3-acetat	P06239	LCK_HUMAN	Homo sapiens (Human)
(20S)-24-ene-3β,20-diol-3-acetat	Q16828	DUS6_HUMAN	Homo sapiens (Human)
(20S)-24-ene-3β,20-diol-3-acetat	Q06124	PTN11_HUMAN	Homo sapiens (Human)
(20S)-24-ene-3β,20-diol-3-acetat	P42768	WASP_HUMAN	Homo sapiens (Human)
(20S)-24-ene-3β,20-diol-3-acetat	P21802	FGFR2_HUMAN	Homo sapiens (Human)
(20S)-24-ene-3β,20-diol-3-acetat	P62508	ERR3_HUMAN	Homo sapiens (Human)
(20S)-24-ene-3β,20-diol-3-acetat	P09874	PARP1_HUMAN	Homo sapiens (Human)
(20S)-24-ene-3β,20-diol-3-acetat	P48449	ERG7_HUMAN	Homo sapiens (Human)
(20S)-24-ene-3β,20-diol-3-acetat	P00326	ADH1G_HUMAN	Homo sapiens (Human)
(20S)-24-ene-3β,20-diol-3-acetat	O15530	PDPK1_HUMAN	Homo sapiens (Human)
(20S)-24-ene-3β,20-diol-3-acetat	Q08499	PDE4D_HUMAN	Homo sapiens (Human)
(20S)-24-ene-3β,20-diol-3-acetat	Q00987	MDM2_HUMAN	Homo sapiens (Human)
(20S)-24-ene-3β,20-diol-3-acetat	P15559	NQO1_HUMAN	Homo sapiens (Human)
(20S)-24-ene-3β,20-diol-3-acetat	Q00796	DHSO_HUMAN	Homo sapiens (Human)
(20S)-24-ene-3β,20-diol-3-acetat	P11474	ERR1_HUMAN	Homo sapiens (Human)
(20S)-24-ene-3β,20-diol-3-acetat	P49841	GSK3B_HUMAN	Homo sapiens (Human)
(20S)-24-ene-3β,20-diol-3-acetat	P0DP23	CALM1_HUMAN	Homo sapiens (Human)
(20S)-24-ene-3β,20-diol-3-acetat	P0DP24	CALM2_HUMAN	Homo sapiens (Human)

(20S)-24-ene-3β,20-diol-3-acetate	P0DP25	CALM3_HUMAN	Homo sapiens (Human)
(20S)-24-ene-3β,20-diol-3-acetate	P08581	MET_HUMAN	Homo sapiens (Human)
(20S)-24-ene-3β,20-diol-3-acetate	P08263	GSTA1_HUMAN	Homo sapiens (Human)
(20S)-24-ene-3β,20-diol-3-acetate	O15540	FABP7_HUMAN	Homo sapiens (Human)
(20S)-24-ene-3β,20-diol-3-acetate	P68400	CSK21_HUMAN	Homo sapiens (Human)
(20S)-24-ene-3β,20-diol-3-acetate	Q13133	NR1H3_HUMAN	Homo sapiens (Human)
(20S)-24-ene-3β,20-diol-3-acetate	Q9BY41	HDAC8_HUMAN	Homo sapiens (Human)
(20S)-24-ene-3β,20-diol-3-acetate	O00204	ST2B1_HUMAN	Homo sapiens (Human)
(20S)-24-ene-3β,20-diol-3-acetate	O60760	HPGDS_HUMAN	Homo sapiens (Human)
(20S)-24-ene-3β,20-diol-3-acetate	O43617	TPPC3_HUMAN	Homo sapiens (Human)
(20S)-24-ene-3β,20-diol-3-acetate	P00746	CFAD_HUMAN	Homo sapiens (Human)
(20S)-24-ene-3β,20-diol-3-acetate	P07858	CATB_HUMAN	Homo sapiens (Human)
(20S)-24-ene-3β,20-diol-3-acetate	P52333	JAK3_HUMAN	Homo sapiens (Human)
(20S)-24-ene-3β,20-diol-3-acetate	Q04828	AK1C1_HUMAN	Homo sapiens (Human)
(20S)-24-ene-3β,20-diol-3-acetate	P34913	HYES_HUMAN	Homo sapiens (Human)
Linarin	P45452	MMP13_HUMAN	Homo sapiens (Human)
Linarin	P17342	ANPRC_HUMAN	Homo sapiens (Human)
Linarin	P04062	GLCM_HUMAN	Homo sapiens (Human)
Linarin	Q9BZR6	RTN4R_HUMAN	Homo sapiens (Human)
Linarin	P56817	BACE1_HUMAN	Homo sapiens (Human)
Linarin	Q12884	SEPR_HUMAN	Homo sapiens (Human)
Linarin	P09211	GSTP1_HUMAN	Homo sapiens (Human)
Linarin	P11309	PIM1_HUMAN	Homo sapiens (Human)
Linarin	P11142	HSP7C_HUMAN	Homo sapiens (Human)
Linarin	Q9NP99	TREM1_HUMAN	Homo sapiens (Human)
Linarin	Q92731	ESR2_HUMAN	Homo sapiens (Human)
Linarin	P00491	PNPH_HUMAN	Homo sapiens (Human)
Linarin	P00533	EGFR_HUMAN	Homo sapiens (Human)
Linarin	O43570	CAH12_HUMAN	Homo sapiens (Human)
Linarin	O14965	AURKA_HUMAN	Homo sapiens (Human)
Linarin	P53582	MAP11_HUMAN	Homo sapiens (Human)
Linarin	O60911	CATL2_HUMAN	Homo sapiens (Human)
Linarin	P24071	FCAR_HUMAN	Homo sapiens (Human)
Linarin	P25774	CATS_HUMAN	Homo sapiens (Human)
Linarin	P62937	PPIA_HUMAN	Homo sapiens (Human)
Linarin	P03372	ESR1_HUMAN	Homo sapiens (Human)
Linarin	P00915	CAH1_HUMAN	Homo sapiens (Human)
Linarin	P07900	HS90A_HUMAN	Homo sapiens (Human)
Linarin	Q16539	MK14_HUMAN	Homo sapiens (Human)
Linarin	P02766	TTHY_HUMAN	Homo sapiens (Human)
Linarin	P06276	CHLE_HUMAN	Homo sapiens (Human)
Linarin	Q07343	PDE4B_HUMAN	Homo sapiens (Human)
Linarin	P00918	CAH2_HUMAN	Homo sapiens (Human)
Linarin	P37173	TGFR2_HUMAN	Homo sapiens (Human)
Linarin	P20248	CCNA2_HUMAN	Homo sapiens (Human)
Linarin	P23946	CMA1_HUMAN	Homo sapiens (Human)
Linarin	Q9BW91	NUDT9_HUMAN	Homo sapiens (Human)
Linarin	P26196	DDX6_HUMAN	Homo sapiens (Human)
Linarin	P55055	NR1H2_HUMAN	Homo sapiens (Human)
Linarin	P53779	MK10_HUMAN	Homo sapiens (Human)
Linarin	O14757	CHK1_HUMAN	Homo sapiens (Human)
Linarin	P08631	HCK_HUMAN	Homo sapiens (Human)
Linarin	P00734	THRB_HUMAN	Homo sapiens (Human)
Linarin	P23141	EST1_HUMAN	Homo sapiens (Human)
Linarin	Q13126	MTAP_HUMAN	Homo sapiens (Human)
Linarin	P62942	FKB1A_HUMAN	Homo sapiens (Human)
Linarin	P42330	AK1C3_HUMAN	Homo sapiens (Human)
Linarin	Q8NBQ5	DHB11_HUMAN	Homo sapiens (Human)

Linarin	P10275	ANDR_HUMAN	Homo sapiens (Human)
Linarin	P24941	CDK2_HUMAN	Homo sapiens (Human)
Linarin	P00749	UROK_HUMAN	Homo sapiens (Human)
Linarin	Q00534	CDK6_HUMAN	Homo sapiens (Human)
Linarin	P11511	CP19A_HUMAN	Homo sapiens (Human)
Linarin	P04745	AMY1_HUMAN	Homo sapiens (Human)
Linarin	P02768	ALBU_HUMAN	Homo sapiens (Human)
Linarin	P42574	CASP3_HUMAN	Homo sapiens (Human)
Linarin	P02652	APOA2_HUMAN	Homo sapiens (Human)
Linarin	P08842	STS_HUMAN	Homo sapiens (Human)
Linarin	Q13231	CHIT1_HUMAN	Homo sapiens (Human)
Linarin	P29218	IMPA1_HUMAN	Homo sapiens (Human)
Linarin	P03950	ANGI_HUMAN	Homo sapiens (Human)
Linarin	P07339	CATD_HUMAN	Homo sapiens (Human)
Linarin	P45983	MK08_HUMAN	Homo sapiens (Human)
Linarin	P00751	CFAB_HUMAN	Homo sapiens (Human)
Linarin	P08254	MMP3_HUMAN	Homo sapiens (Human)
Linarin	P06401	PRGR_HUMAN	Homo sapiens (Human)
Linarin	P54760	EPHB4_HUMAN	Homo sapiens (Human)
Linarin	Q16769	QPCT_HUMAN	Homo sapiens (Human)
Linarin	P25311	ZA2G_HUMAN	Homo sapiens (Human)
Linarin	O76074	PDE5A_HUMAN	Homo sapiens (Human)
Linarin	P07858	CATB_HUMAN	Homo sapiens (Human)
Linarin	P49841	GSK3B_HUMAN	Homo sapiens (Human)
Linarin	Q05315	LEG10_HUMAN	Homo sapiens (Human)
Linarin	P15121	ALDR_HUMAN	Homo sapiens (Human)
Linarin	Q13370	PDE3B_HUMAN	Homo sapiens (Human)
Linarin	P53355	DAPK1_HUMAN	Homo sapiens (Human)
Linarin	P27338	AOFB_HUMAN	Homo sapiens (Human)
Linarin	P04278	SHBG_HUMAN	Homo sapiens (Human)
Linarin	P19367	HXK1_HUMAN	Homo sapiens (Human)
Linarin	P48449	ERG7_HUMAN	Homo sapiens (Human)
Linarin	P12931	SRC_HUMAN	Homo sapiens (Human)
Linarin	P18075	BMP7_HUMAN	Homo sapiens (Human)
Linarin	P63316	TNNC1_HUMAN	Homo sapiens (Human)
Linarin	O15382	BCAT2_HUMAN	Homo sapiens (Human)
Linarin	P02743	SAMP_HUMAN	Homo sapiens (Human)
Linarin	P29474	NOS3_HUMAN	Homo sapiens (Human)
Linarin	P47929	LEG7_HUMAN	Homo sapiens (Human)
Linarin	P16083	NQO2_HUMAN	Homo sapiens (Human)
Linarin	P00390	GSHR_HUMAN	Homo sapiens (Human)
Linarin	P14174	MIF_HUMAN	Homo sapiens (Human)
Linarin	P36897	TGFR1_HUMAN	Homo sapiens (Human)
Linarin	P19971	TYPH_HUMAN	Homo sapiens (Human)
Linarin	P78368	KC1G2_HUMAN	Homo sapiens (Human)
Linarin	P14061	DHB1_HUMAN	Homo sapiens (Human)
Linarin	P78536	ADA17_HUMAN	Homo sapiens (Human)
Linarin	P04179	SODM_HUMAN	Homo sapiens (Human)
Linarin	P43155	CACP_HUMAN	Homo sapiens (Human)
Linarin	P00374	DYR_HUMAN	Homo sapiens (Human)
Linarin	Q08499	PDE4D_HUMAN	Homo sapiens (Human)
Linarin	P01709	LV208_HUMAN	Homo sapiens (Human)
Linarin	P00439	PH4H_HUMAN	Homo sapiens (Human)
Linarin	P35968	VGFR2_HUMAN	Homo sapiens (Human)
Linarin	P62166	NCS1_HUMAN	Homo sapiens (Human)
Linarin	P06737	PYGL_HUMAN	Homo sapiens (Human)
Linarin	P27487	DPP4_HUMAN	Homo sapiens (Human)
Linarin	P22894	MMP8_HUMAN	Homo sapiens (Human)

Linarin	P00736	C1R_HUMAN	Homo sapiens (Human)
Linarin	P62508	ERR3_HUMAN	Homo sapiens (Human)
Linarin	P08758	ANXA5_HUMAN	Homo sapiens (Human)
Linarin	P23526	SAHH_HUMAN	Homo sapiens (Human)
Linarin	P37231	PPARG_HUMAN	Homo sapiens (Human)
Linarin	P04746	AMYP_HUMAN	Homo sapiens (Human)
Linarin	P17707	DCAM_HUMAN	Homo sapiens (Human)
Linarin	Q03181	PPARD_HUMAN	Homo sapiens (Human)
Linarin	P15056	BRAF_HUMAN	Homo sapiens (Human)
Linarin	P55210	CASP7_HUMAN	Homo sapiens (Human)
Linarin	Q00796	DHSO_HUMAN	Homo sapiens (Human)
Linarin	P35558	PCKGC_HUMAN	Homo sapiens (Human)
Linarin	P00746	CFAD_HUMAN	Homo sapiens (Human)
Linarin	P49354	FNTA_HUMAN	Homo sapiens (Human)
Linarin	P52732	KIF11_HUMAN	Homo sapiens (Human)
Linarin	Q9NVS9	PNPO_HUMAN	Homo sapiens (Human)
Linarin	P00742	FA10_HUMAN	Homo sapiens (Human)
Linarin	P53350	PLK1_HUMAN	Homo sapiens (Human)
Linarin	O15496	PA2GX_HUMAN	Homo sapiens (Human)
Linarin	P16152	CBR1_HUMAN	Homo sapiens (Human)
Linarin	O15530	PDPK1_HUMAN	Homo sapiens (Human)
Linarin	P11362	FGFR1_HUMAN	Homo sapiens (Human)
Linarin	Q96T66	NMNA3_HUMAN	Homo sapiens (Human)
Linarin	Q02127	PYRD_HUMAN	Homo sapiens (Human)
Linarin	P32320	CDD_HUMAN	Homo sapiens (Human)
Linarin	P43235	CATK_HUMAN	Homo sapiens (Human)
Linarin	Q06520	ST2A1_HUMAN	Homo sapiens (Human)
Linarin	Q15382	RHEB_HUMAN	Homo sapiens (Human)
Linarin	P05162	LEG2_HUMAN	Homo sapiens (Human)
Linarin	P28482	MK01_HUMAN	Homo sapiens (Human)
Linarin	P00797	RENI_HUMAN	Homo sapiens (Human)
Linarin	P15559	NQO1_HUMAN	Homo sapiens (Human)
Linarin	P04818	TYSY_HUMAN	Homo sapiens (Human)
Linarin	Q07912	ACK1_HUMAN	Homo sapiens (Human)
Linarin	P08069	IGF1R_HUMAN	Homo sapiens (Human)
Linarin	P15153	RAC2_HUMAN	Homo sapiens (Human)
Linarin	P09960	LKHA4_HUMAN	Homo sapiens (Human)
Linarin	P00325	ADH1B_HUMAN	Homo sapiens (Human)
Linarin	Q99933	BAG1_HUMAN	Homo sapiens (Human)
Linarin	P49789	FHIT_HUMAN	Homo sapiens (Human)
Linarin	Q16836	HCDH_HUMAN	Homo sapiens (Human)
Linarin	Q16828	DUS6_HUMAN	Homo sapiens (Human)
Linarin	P18031	PTN1_HUMAN	Homo sapiens (Human)
Linarin	P05091	ALDH2_HUMAN	Homo sapiens (Human)
Linarin	P31749	AKT1_HUMAN	Homo sapiens (Human)
Linarin	P60174	TPIS_HUMAN	Homo sapiens (Human)
Linarin	P02774	VTDB_HUMAN	Homo sapiens (Human)
Linarin	P62491	RB11A_HUMAN	Homo sapiens (Human)
Linarin	P84077	ARF1_HUMAN	Homo sapiens (Human)
Linarin	P08235	MCR_HUMAN	Homo sapiens (Human)
Linarin	Q04760	LGUL_HUMAN	Homo sapiens (Human)
Linarin	Q96AZ6	ISG20_HUMAN	Homo sapiens (Human)
Linarin	P68400	CSK21_HUMAN	Homo sapiens (Human)
Linarin	P28845	DHI1_HUMAN	Homo sapiens (Human)
Linarin	P27707	DCK_HUMAN	Homo sapiens (Human)
Linarin	Q9P2W7	B3GA1_HUMAN	Homo sapiens (Human)
Linarin	P08246	ELNE_HUMAN	Homo sapiens (Human)
Linarin	P01009	A1AT_HUMAN	Homo sapiens (Human)

Linarin	Q10588	BST1_HUMAN	Homo sapiens (Human)
Linarin	P08581	MET_HUMAN	Homo sapiens (Human)
Linarin	Q04828	AK1C1_HUMAN	Homo sapiens (Human)
Linarin	P07195	LDHB_HUMAN	Homo sapiens (Human)
Linarin	P35228	NOS2_HUMAN	Homo sapiens (Human)
Linarin	P30043	BLVRB_HUMAN	Homo sapiens (Human)
Linarin	P15090	FABP4_HUMAN	Homo sapiens (Human)
Linarin	P06239	LCK_HUMAN	Homo sapiens (Human)
Linarin	P17931	LEG3_HUMAN	Homo sapiens (Human)
Linarin	P15309	PPAP_HUMAN	Homo sapiens (Human)
Linarin	P49137	MAPK2_HUMAN	Homo sapiens (Human)
acacetin	Q16678	CP1B1_HUMAN	Homo sapiens (Human)
acacetin	P08631	HCK_HUMAN	Homo sapiens (Human)
acacetin	P00918	CAH2_HUMAN	Homo sapiens (Human)
acacetin	P03372	ESR1_HUMAN	Homo sapiens (Human)
acacetin	P00915	CAH1_HUMAN	Homo sapiens (Human)
acacetin	P06276	CHLE_HUMAN	Homo sapiens (Human)
acacetin	P24941	CDK2_HUMAN	Homo sapiens (Human)
acacetin	P11309	PIM1_HUMAN	Homo sapiens (Human)
acacetin	P00734	THRB_HUMAN	Homo sapiens (Human)
acacetin	P10275	ANDR_HUMAN	Homo sapiens (Human)
acacetin	P11511	CP19A_HUMAN	Homo sapiens (Human)
acacetin	P00390	GSHR_HUMAN	Homo sapiens (Human)
acacetin	P02768	ALBU_HUMAN	Homo sapiens (Human)
acacetin	P02652	APOA2_HUMAN	Homo sapiens (Human)
acacetin	Q00534	CDK6_HUMAN	Homo sapiens (Human)
acacetin	P08842	STS_HUMAN	Homo sapiens (Human)
acacetin	P02766	TTHY_HUMAN	Homo sapiens (Human)
acacetin	P00751	CFAB_HUMAN	Homo sapiens (Human)
acacetin	P53779	MK10_HUMAN	Homo sapiens (Human)
acacetin	P03950	ANGI_HUMAN	Homo sapiens (Human)
acacetin	P29474	NOS3_HUMAN	Homo sapiens (Human)
acacetin	O15382	BCAT2_HUMAN	Homo sapiens (Human)
acacetin	P54760	EPHB4_HUMAN	Homo sapiens (Human)
acacetin	Q92731	ESR2_HUMAN	Homo sapiens (Human)
acacetin	P14061	DHB1_HUMAN	Homo sapiens (Human)
acacetin	P78536	ADA17_HUMAN	Homo sapiens (Human)
acacetin	P04278	SHBG_HUMAN	Homo sapiens (Human)
acacetin	P27338	AOFB_HUMAN	Homo sapiens (Human)
acacetin	P11142	HSP7C_HUMAN	Homo sapiens (Human)
acacetin	P35968	VGFR2_HUMAN	Homo sapiens (Human)
acacetin	Q16539	MK14_HUMAN	Homo sapiens (Human)
acacetin	P15056	BRAF_HUMAN	Homo sapiens (Human)
acacetin	P06401	PRGR_HUMAN	Homo sapiens (Human)
acacetin	P14174	MIF_HUMAN	Homo sapiens (Human)
acacetin	P15559	NQO1_HUMAN	Homo sapiens (Human)
acacetin	P45983	MK08_HUMAN	Homo sapiens (Human)
acacetin	O14965	AURKA_HUMAN	Homo sapiens (Human)
acacetin	P15121	ALDR_HUMAN	Homo sapiens (Human)
acacetin	Q16828	DUS6_HUMAN	Homo sapiens (Human)
acacetin	P06737	PYGL_HUMAN	Homo sapiens (Human)
acacetin	P62508	ERR3_HUMAN	Homo sapiens (Human)
acacetin	O14757	CHK1_HUMAN	Homo sapiens (Human)
acacetin	P45452	MMP13_HUMAN	Homo sapiens (Human)
acacetin	Q06520	ST2A1_HUMAN	Homo sapiens (Human)
acacetin	P56817	BACE1_HUMAN	Homo sapiens (Human)
acacetin	P23141	EST1_HUMAN	Homo sapiens (Human)
acacetin	P00491	PNPH_HUMAN	Homo sapiens (Human)

acacetin	O15530	PDPK1_HUMAN	Homo sapiens (Human)
acacetin	Q08499	PDE4D_HUMAN	Homo sapiens (Human)
acacetin	P00374	DYR_HUMAN	Homo sapiens (Human)
acacetin	P02774	VTDB_HUMAN	Homo sapiens (Human)
acacetin	P07900	HS90A_HUMAN	Homo sapiens (Human)
acacetin	P11766	ADHX_HUMAN	Homo sapiens (Human)
acacetin	P09960	LKHA4_HUMAN	Homo sapiens (Human)
acacetin	P20248	CCNA2_HUMAN	Homo sapiens (Human)
butin	P11511	CP19A_HUMAN	Homo sapiens (Human)
butin	P43166	CAH7_HUMAN	Homo sapiens (Human)
butin	O43570	CAH12_HUMAN	Homo sapiens (Human)
butin	P22748	CAH4_HUMAN	Homo sapiens (Human)
butin	Q16678	CP1B1_HUMAN	Homo sapiens (Human)
butin	P08631	HCK_HUMAN	Homo sapiens (Human)
butin	P00918	CAH2_HUMAN	Homo sapiens (Human)
butin	P03372	ESR1_HUMAN	Homo sapiens (Human)
butin	P00915	CAH1_HUMAN	Homo sapiens (Human)
butin	P06276	CHLE_HUMAN	Homo sapiens (Human)
butin	P24941	CDK2_HUMAN	Homo sapiens (Human)
butin	P11309	PIM1_HUMAN	Homo sapiens (Human)
butin	P00734	THRB_HUMAN	Homo sapiens (Human)
butin	P10275	ANDR_HUMAN	Homo sapiens (Human)
butin	P11511	CP19A_HUMAN	Homo sapiens (Human)
butin	P00390	GSHR_HUMAN	Homo sapiens (Human)
butin	P02768	ALBU_HUMAN	Homo sapiens (Human)
butin	P02652	APOA2_HUMAN	Homo sapiens (Human)
butin	Q00534	CDK6_HUMAN	Homo sapiens (Human)
butin	P08842	STS_HUMAN	Homo sapiens (Human)
butin	P02766	TTHY_HUMAN	Homo sapiens (Human)
butin	P00751	CFAB_HUMAN	Homo sapiens (Human)
butin	P53779	MK10_HUMAN	Homo sapiens (Human)
butin	P03950	ANGI_HUMAN	Homo sapiens (Human)
butin	P29474	NOS3_HUMAN	Homo sapiens (Human)
butin	O15382	BCAT2_HUMAN	Homo sapiens (Human)
butin	P54760	EPHB4_HUMAN	Homo sapiens (Human)
butin	Q92731	ESR2_HUMAN	Homo sapiens (Human)
butin	P14061	DHB1_HUMAN	Homo sapiens (Human)
butin	P78536	ADA17_HUMAN	Homo sapiens (Human)
butin	P04278	SHBG_HUMAN	Homo sapiens (Human)
butin	P27338	AOFB_HUMAN	Homo sapiens (Human)
butin	P11142	HSP7C_HUMAN	Homo sapiens (Human)
butin	P35968	VGFR2_HUMAN	Homo sapiens (Human)
butin	Q16539	MK14_HUMAN	Homo sapiens (Human)
butin	P15056	BRAF_HUMAN	Homo sapiens (Human)
butin	P06401	PRGR_HUMAN	Homo sapiens (Human)
butin	P14174	MIF_HUMAN	Homo sapiens (Human)
butin	P15559	NQO1_HUMAN	Homo sapiens (Human)
butin	P45983	MK08_HUMAN	Homo sapiens (Human)
butin	O14965	AURKA_HUMAN	Homo sapiens (Human)
butin	P15121	ALDR_HUMAN	Homo sapiens (Human)
butin	Q16828	DUS6_HUMAN	Homo sapiens (Human)
butin	P06737	PYGL_HUMAN	Homo sapiens (Human)
butin	P62508	ERR3_HUMAN	Homo sapiens (Human)
butin	O14757	CHK1_HUMAN	Homo sapiens (Human)
butin	P45452	MMP13_HUMAN	Homo sapiens (Human)
butin	Q06520	ST2A1_HUMAN	Homo sapiens (Human)
butin	P56817	BACE1_HUMAN	Homo sapiens (Human)
butin	P23141	EST1_HUMAN	Homo sapiens (Human)

butin	P00491	PNPH_HUMAN	Homo sapiens (Human)
butin	O15530	PDPK1_HUMAN	Homo sapiens (Human)
butin	Q08499	PDE4D_HUMAN	Homo sapiens (Human)
butin	P00374	DYR_HUMAN	Homo sapiens (Human)
butin	P02774	VTDB_HUMAN	Homo sapiens (Human)
butin	P07900	HS90A_HUMAN	Homo sapiens (Human)
butin	P11766	ADHX_HUMAN	Homo sapiens (Human)
butin	P09960	LKHA4_HUMAN	Homo sapiens (Human)
butin	P20248	CCNA2_HUMAN	Homo sapiens (Human)
Pratensein	P00491	PNPH_HUMAN	Homo sapiens (Human)
Pratensein	P03372	ESR1_HUMAN	Homo sapiens (Human)
Pratensein	P00918	CAH2_HUMAN	Homo sapiens (Human)
Pratensein	Q92731	ESR2_HUMAN	Homo sapiens (Human)
Pratensein	P08254	MMP3_HUMAN	Homo sapiens (Human)
Pratensein	P11309	PIM1_HUMAN	Homo sapiens (Human)
Pratensein	P00915	CAH1_HUMAN	Homo sapiens (Human)
Pratensein	P35968	VGFR2_HUMAN	Homo sapiens (Human)
Pratensein	P53779	MK10_HUMAN	Homo sapiens (Human)
Pratensein	Q12884	SEPR_HUMAN	Homo sapiens (Human)
Pratensein	P06276	CHLE_HUMAN	Homo sapiens (Human)
Pratensein	Q16539	MK14_HUMAN	Homo sapiens (Human)
Pratensein	P23141	EST1_HUMAN	Homo sapiens (Human)
Pratensein	P56817	BACE1_HUMAN	Homo sapiens (Human)
Pratensein	O14757	CHK1_HUMAN	Homo sapiens (Human)
Pratensein	P10275	ANDR_HUMAN	Homo sapiens (Human)
Pratensein	P00751	CFAB_HUMAN	Homo sapiens (Human)
Pratensein	P45983	MK08_HUMAN	Homo sapiens (Human)
Pratensein	P20248	CCNA2_HUMAN	Homo sapiens (Human)
Pratensein	P24941	CDK2_HUMAN	Homo sapiens (Human)
Pratensein	P78536	ADA17_HUMAN	Homo sapiens (Human)
Pratensein	P02768	ALBU_HUMAN	Homo sapiens (Human)
Pratensein	P11142	HSP7C_HUMAN	Homo sapiens (Human)
Pratensein	P08842	STS_HUMAN	Homo sapiens (Human)
Pratensein	P02652	APOA2_HUMAN	Homo sapiens (Human)
Pratensein	O14965	AURKA_HUMAN	Homo sapiens (Human)
Pratensein	P04062	GLCM_HUMAN	Homo sapiens (Human)
Pratensein	P07339	CATD_HUMAN	Homo sapiens (Human)
Pratensein	P06401	PRGR_HUMAN	Homo sapiens (Human)
Pratensein	P11362	FGFR1_HUMAN	Homo sapiens (Human)
Pratensein	P00439	PH4H_HUMAN	Homo sapiens (Human)
Pratensein	P54760	EPHB4_HUMAN	Homo sapiens (Human)
Pratensein	P45452	MMP13_HUMAN	Homo sapiens (Human)
Pratensein	P03950	ANGI_HUMAN	Homo sapiens (Human)
Pratensein	O15382	BCAT2_HUMAN	Homo sapiens (Human)
Pratensein	Q13126	MTAP_HUMAN	Homo sapiens (Human)
Pratensein	P27338	AOFB_HUMAN	Homo sapiens (Human)
Pratensein	P11511	CP19A_HUMAN	Homo sapiens (Human)
Pratensein	Q00534	CDK6_HUMAN	Homo sapiens (Human)
Pratensein	P04278	SHBG_HUMAN	Homo sapiens (Human)
Pratensein	Q06520	ST2A1_HUMAN	Homo sapiens (Human)
Pratensein	P00734	THRB_HUMAN	Homo sapiens (Human)
Pratensein	P00533	EGFR_HUMAN	Homo sapiens (Human)
Pratensein	P14174	MIF_HUMAN	Homo sapiens (Human)
Pratensein	P22894	MMP8_HUMAN	Homo sapiens (Human)
Pratensein	P09960	LKHA4_HUMAN	Homo sapiens (Human)
Pratensein	P15559	NQO1_HUMAN	Homo sapiens (Human)
Pratensein	Q08499	PDE4D_HUMAN	Homo sapiens (Human)
Pratensein	P14061	DHB1_HUMAN	Homo sapiens (Human)

Pratensein	P06737	PYGL_HUMAN	Homo sapiens (Human)
Pratensein	Q16828	DUS6_HUMAN	Homo sapiens (Human)
Pratensein	P02766	TTHY_HUMAN	Homo sapiens (Human)
Pratensein	P18075	BMP7_HUMAN	Homo sapiens (Human)
Pratensein	P15121	ALDR_HUMAN	Homo sapiens (Human)
Pratensein	P78368	KC1G2_HUMAN	Homo sapiens (Human)
Pratensein	P00374	DYR_HUMAN	Homo sapiens (Human)
Pratensein	Q07343	PDE4B_HUMAN	Homo sapiens (Human)
Pratensein	P00390	GSHR_HUMAN	Homo sapiens (Human)
Pratensein	P27487	DPP4_HUMAN	Homo sapiens (Human)
Pratensein	P50579	MAP2_HUMAN	Homo sapiens (Human)
Pratensein	P62508	ERR3_HUMAN	Homo sapiens (Human)
Pratensein	P28845	DHI1_HUMAN	Homo sapiens (Human)
Pratensein	P07900	HS90A_HUMAN	Homo sapiens (Human)
Pratensein	Q03181	PPARD_HUMAN	Homo sapiens (Human)
Pratensein	P52732	KIF11_HUMAN	Homo sapiens (Human)
Pratensein	P53350	PLK1_HUMAN	Homo sapiens (Human)
Pratensein	Q13370	PDE3B_HUMAN	Homo sapiens (Human)
Pratensein	P35558	PCKGC_HUMAN	Homo sapiens (Human)
Pratensein	P31749	AKT1_HUMAN	Homo sapiens (Human)
Pratensein	O15530	PDPK1_HUMAN	Homo sapiens (Human)
Pratensein	P00742	FA10_HUMAN	Homo sapiens (Human)
Pratensein	P29474	NOS3_HUMAN	Homo sapiens (Human)
Pratensein	P12931	SRC_HUMAN	Homo sapiens (Human)
Pratensein	P07858	CATB_HUMAN	Homo sapiens (Human)
Pratensein	Q02127	PYRD_HUMAN	Homo sapiens (Human)
Pratensein	P04745	AMY1_HUMAN	Homo sapiens (Human)
Pratensein	P16152	CBR1_HUMAN	Homo sapiens (Human)
Pratensein	Q07912	ACK1_HUMAN	Homo sapiens (Human)
Pratensein	P02774	VTDB_HUMAN	Homo sapiens (Human)
Pratensein	P04818	TYSY_HUMAN	Homo sapiens (Human)
Pratensein	Q15382	RHEB_HUMAN	Homo sapiens (Human)
Pratensein	P49354	FNTA_HUMAN	Homo sapiens (Human)
Pratensein	P16442	BGAT_HUMAN	Homo sapiens (Human)
demethylwedelolactone	P20248	CCNA2_HUMAN	Homo sapiens (Human)
demethylwedelolactone	P10275	ANDR_HUMAN	Homo sapiens (Human)
demethylwedelolactone	P24941	CDK2_HUMAN	Homo sapiens (Human)
demethylwedelolactone	P03372	ESR1_HUMAN	Homo sapiens (Human)
demethylwedelolactone	P00734	THRB_HUMAN	Homo sapiens (Human)
demethylwedelolactone	P06276	CHLE_HUMAN	Homo sapiens (Human)
demethylwedelolactone	P53779	MK10_HUMAN	Homo sapiens (Human)
demethylwedelolactone	P00918	CAH2_HUMAN	Homo sapiens (Human)
demethylwedelolactone	Q16740	CLPP_HUMAN	Homo sapiens (Human)
demethylwedelolactone	Q12884	SEPR_HUMAN	Homo sapiens (Human)
demethylwedelolactone	P45983	MK08_HUMAN	Homo sapiens (Human)
demethylwedelolactone	P48736	PK3CG_HUMAN	Homo sapiens (Human)
demethylwedelolactone	P00915	CAH1_HUMAN	Homo sapiens (Human)
demethylwedelolactone	P11511	CP19A_HUMAN	Homo sapiens (Human)
demethylwedelolactone	P62937	PPIA_HUMAN	Homo sapiens (Human)
demethylwedelolactone	P00491	PNPH_HUMAN	Homo sapiens (Human)
demethylwedelolactone	Q16539	MK14_HUMAN	Homo sapiens (Human)
demethylwedelolactone	P12643	BMP2_HUMAN	Homo sapiens (Human)
demethylwedelolactone	P00533	EGFR_HUMAN	Homo sapiens (Human)
demethylwedelolactone	P02652	APOA2_HUMAN	Homo sapiens (Human)
demethylwedelolactone	P23141	EST1_HUMAN	Homo sapiens (Human)
demethylwedelolactone	P00751	CFAB_HUMAN	Homo sapiens (Human)
demethylwedelolactone	Q08499	PDE4D_HUMAN	Homo sapiens (Human)
demethylwedelolactone	P08842	STS_HUMAN	Homo sapiens (Human)

demethylwedelolactone	P11309	PIM1_HUMAN	Homo sapiens (Human)
demethylwedelolactone	Q8NBQ5	DHB11_HUMAN	Homo sapiens (Human)
demethylwedelolactone	P02768	ALBU_HUMAN	Homo sapiens (Human)
demethylwedelolactone	O14965	AURKA_HUMAN	Homo sapiens (Human)
demethylwedelolactone	Q07343	PDE4B_HUMAN	Homo sapiens (Human)
demethylwedelolactone	O43570	CAH12_HUMAN	Homo sapiens (Human)
demethylwedelolactone	P08254	MMP3_HUMAN	Homo sapiens (Human)
demethylwedelolactone	P07900	HS90A_HUMAN	Homo sapiens (Human)
demethylwedelolactone	P35221	CTNA1_HUMAN	Homo sapiens (Human)
demethylwedelolactone	P56817	BACE1_HUMAN	Homo sapiens (Human)
demethylwedelolactone	P54760	EPHB4_HUMAN	Homo sapiens (Human)
demethylwedelolactone	P52732	KIF11_HUMAN	Homo sapiens (Human)
demethylwedelolactone	P00390	GSHR_HUMAN	Homo sapiens (Human)
demethylwedelolactone	P00439	PH4H_HUMAN	Homo sapiens (Human)
demethylwedelolactone	P62508	ERR3_HUMAN	Homo sapiens (Human)
demethylwedelolactone	P35968	VGFR2_HUMAN	Homo sapiens (Human)
demethylwedelolactone	P14061	DHB1_HUMAN	Homo sapiens (Human)
demethylwedelolactone	P53041	PPP5_HUMAN	Homo sapiens (Human)
demethylwedelolactone	P11766	ADHX_HUMAN	Homo sapiens (Human)
demethylwedelolactone	P12931	SRC_HUMAN	Homo sapiens (Human)
demethylwedelolactone	O14757	CHK1_HUMAN	Homo sapiens (Human)
demethylwedelolactone	P80188	NGAL_HUMAN	Homo sapiens (Human)
demethylwedelolactone	Q13126	MTAP_HUMAN	Homo sapiens (Human)
demethylwedelolactone	Q16828	DUS6_HUMAN	Homo sapiens (Human)
demethylwedelolactone	P14174	MIF_HUMAN	Homo sapiens (Human)
demethylwedelolactone	P02766	TTHY_HUMAN	Homo sapiens (Human)
demethylwedelolactone	P15121	ALDR_HUMAN	Homo sapiens (Human)
demethylwedelolactone	P11142	HSP7C_HUMAN	Homo sapiens (Human)
demethylwedelolactone	P16083	NQO2_HUMAN	Homo sapiens (Human)
demethylwedelolactone	O15530	PDPK1_HUMAN	Homo sapiens (Human)
demethylwedelolactone	P42330	AK1C3_HUMAN	Homo sapiens (Human)
demethylwedelolactone	P00374	DYR_HUMAN	Homo sapiens (Human)
demethylwedelolactone	P29474	NOS3_HUMAN	Homo sapiens (Human)
demethylwedelolactone	P22894	MMP8_HUMAN	Homo sapiens (Human)
demethylwedelolactone	P03950	ANGI_HUMAN	Homo sapiens (Human)
demethylwedelolactone	P00746	CFAD_HUMAN	Homo sapiens (Human)
demethylwedelolactone	P06401	PRGR_HUMAN	Homo sapiens (Human)
demethylwedelolactone	P54577	SYYC_HUMAN	Homo sapiens (Human)
demethylwedelolactone	P04278	SHBG_HUMAN	Homo sapiens (Human)
demethylwedelolactone	P52895	AK1C2_HUMAN	Homo sapiens (Human)
demethylwedelolactone	P78536	ADA17_HUMAN	Homo sapiens (Human)
demethylwedelolactone	Q06520	ST2A1_HUMAN	Homo sapiens (Human)
demethylwedelolactone	Q13370	PDE3B_HUMAN	Homo sapiens (Human)
demethylwedelolactone	P28845	DHI1_HUMAN	Homo sapiens (Human)
demethylwedelolactone	O76074	PDE5A_HUMAN	Homo sapiens (Human)
demethylwedelolactone	P18075	BMP7_HUMAN	Homo sapiens (Human)
demethylwedelolactone	Q03181	PPARD_HUMAN	Homo sapiens (Human)
demethylwedelolactone	P04818	TYSY_HUMAN	Homo sapiens (Human)
demethylwedelolactone	P04179	SODM_HUMAN	Homo sapiens (Human)
demethylwedelolactone	P11362	FGFR1_HUMAN	Homo sapiens (Human)
demethylwedelolactone	P45452	MMP13_HUMAN	Homo sapiens (Human)
demethylwedelolactone	P49763	PLGF_HUMAN	Homo sapiens (Human)
demethylwedelolactone	P02774	VTDB_HUMAN	Homo sapiens (Human)
wedelolactone	P00918	CAH2_HUMAN	Homo sapiens (Human)
wedelolactone	P03372	ESR1_HUMAN	Homo sapiens (Human)
wedelolactone	P00734	THRB_HUMAN	Homo sapiens (Human)
wedelolactone	P53779	MK10_HUMAN	Homo sapiens (Human)
wedelolactone	Q16740	CLPP_HUMAN	Homo sapiens (Human)

wedelolactone	P00915	CAH1_HUMAN	Homo sapiens (Human)
wedelolactone	P10275	ANDR_HUMAN	Homo sapiens (Human)
wedelolactone	P45983	MK08_HUMAN	Homo sapiens (Human)
wedelolactone	P06276	CHLE_HUMAN	Homo sapiens (Human)
wedelolactone	Q16539	MK14_HUMAN	Homo sapiens (Human)
wedelolactone	P62937	PPIA_HUMAN	Homo sapiens (Human)
wedelolactone	P48736	PK3CG_HUMAN	Homo sapiens (Human)
wedelolactone	P11511	CP19A_HUMAN	Homo sapiens (Human)
wedelolactone	Q12884	SEPR_HUMAN	Homo sapiens (Human)
wedelolactone	P00491	PNPH_HUMAN	Homo sapiens (Human)
wedelolactone	P12643	BMP2_HUMAN	Homo sapiens (Human)
wedelolactone	P00533	EGFR_HUMAN	Homo sapiens (Human)
wedelolactone	P02652	APOA2_HUMAN	Homo sapiens (Human)
wedelolactone	P53041	PPP5_HUMAN	Homo sapiens (Human)
wedelolactone	P09211	GSTP1_HUMAN	Homo sapiens (Human)
wedelolactone	P23141	EST1_HUMAN	Homo sapiens (Human)
wedelolactone	P08842	STS_HUMAN	Homo sapiens (Human)
wedelolactone	P00751	CFAB_HUMAN	Homo sapiens (Human)
wedelolactone	Q08499	PDE4D_HUMAN	Homo sapiens (Human)
wedelolactone	P24941	CDK2_HUMAN	Homo sapiens (Human)
wedelolactone	Q8NBQ5	DHB11_HUMAN	Homo sapiens (Human)
wedelolactone	P11309	PIM1_HUMAN	Homo sapiens (Human)
wedelolactone	P02768	ALBU_HUMAN	Homo sapiens (Human)
wedelolactone	O14965	AURKA_HUMAN	Homo sapiens (Human)
wedelolactone	P11142	HSP7C_HUMAN	Homo sapiens (Human)
wedelolactone	Q07343	PDE4B_HUMAN	Homo sapiens (Human)
wedelolactone	O43570	CAH12_HUMAN	Homo sapiens (Human)
wedelolactone	P55055	NR1H2_HUMAN	Homo sapiens (Human)
wedelolactone	P08254	MMP3_HUMAN	Homo sapiens (Human)
wedelolactone	P20248	CCNA2_HUMAN	Homo sapiens (Human)
wedelolactone	P07900	HS90A_HUMAN	Homo sapiens (Human)
wedelolactone	Q92731	ESR2_HUMAN	Homo sapiens (Human)
wedelolactone	P35221	CTNA1_HUMAN	Homo sapiens (Human)
wedelolactone	P02766	TTHY_HUMAN	Homo sapiens (Human)
wedelolactone	P16083	NQO2_HUMAN	Homo sapiens (Human)
wedelolactone	P52732	KIF11_HUMAN	Homo sapiens (Human)
wedelolactone	P80188	NGAL_HUMAN	Homo sapiens (Human)
wedelolactone	P00439	PH4H_HUMAN	Homo sapiens (Human)
wedelolactone	P45452	MMP13_HUMAN	Homo sapiens (Human)
wedelolactone	P42330	AK1C3_HUMAN	Homo sapiens (Human)
wedelolactone	O14757	CHK1_HUMAN	Homo sapiens (Human)
wedelolactone	P00390	GSHR_HUMAN	Homo sapiens (Human)
wedelolactone	P56817	BACE1_HUMAN	Homo sapiens (Human)
wedelolactone	P54760	EPHB4_HUMAN	Homo sapiens (Human)
wedelolactone	P29474	NOS3_HUMAN	Homo sapiens (Human)
wedelolactone	O76074	PDE5A_HUMAN	Homo sapiens (Human)
wedelolactone	P49763	PLGF_HUMAN	Homo sapiens (Human)
wedelolactone	P12931	SRC_HUMAN	Homo sapiens (Human)
wedelolactone	P11766	ADHX_HUMAN	Homo sapiens (Human)
wedelolactone	P14174	MIF_HUMAN	Homo sapiens (Human)
wedelolactone	P62508	ERR3_HUMAN	Homo sapiens (Human)
wedelolactone	Q13126	MTAP_HUMAN	Homo sapiens (Human)
wedelolactone	Q16828	DUS6_HUMAN	Homo sapiens (Human)
wedelolactone	P06401	PRGR_HUMAN	Homo sapiens (Human)
wedelolactone	O15530	PDPK1_HUMAN	Homo sapiens (Human)
wedelolactone	P15121	ALDR_HUMAN	Homo sapiens (Human)
wedelolactone	P43235	CATK_HUMAN	Homo sapiens (Human)
wedelolactone	P22894	MMP8_HUMAN	Homo sapiens (Human)

wedelolactone	P00374	DYR_HUMAN	Homo sapiens (Human)
wedelolactone	P03950	ANGI_HUMAN	Homo sapiens (Human)
wedelolactone	P04278	SHBG_HUMAN	Homo sapiens (Human)
wedelolactone	P00746	CFAD_HUMAN	Homo sapiens (Human)
wedelolactone	P78536	ADA17_HUMAN	Homo sapiens (Human)
wedelolactone	O15382	BCAT2_HUMAN	Homo sapiens (Human)
wedelolactone	P18075	BMP7_HUMAN	Homo sapiens (Human)
wedelolactone	Q13370	PDE3B_HUMAN	Homo sapiens (Human)
wedelolactone	P28845	DHI1_HUMAN	Homo sapiens (Human)
wedelolactone	P35968	VGFR2_HUMAN	Homo sapiens (Human)
wedelolactone	P14061	DHB1_HUMAN	Homo sapiens (Human)
wedelolactone	Q03181	PPARD_HUMAN	Homo sapiens (Human)
wedelolactone	P11362	FGFR1_HUMAN	Homo sapiens (Human)
wedelolactone	P04818	TYSY_HUMAN	Homo sapiens (Human)
wedelolactone	Q06520	ST2A1_HUMAN	Homo sapiens (Human)
wedelolactone	P00742	FA10_HUMAN	Homo sapiens (Human)
wedelolactone	P04179	SODM_HUMAN	Homo sapiens (Human)
wedelolactone	P02774	VTDB_HUMAN	Homo sapiens (Human)
wedelolactone	Q00796	DHSO_HUMAN	Homo sapiens (Human)
wedelolactone	P09917	LOX5_HUMAN	Homo sapiens (Human)
wedelolactone	P16152	CBR1_HUMAN	Homo sapiens (Human)
luteolin	P11511	CP19A_HUMAN	Homo sapiens (Human)
luteolin	P00918	CAH2_HUMAN	Homo sapiens (Human)
luteolin	P10275	ANDR_HUMAN	Homo sapiens (Human)
luteolin	P00734	THRB_HUMAN	Homo sapiens (Human)
luteolin	P08631	HCK_HUMAN	Homo sapiens (Human)
luteolin	P00915	CAH1_HUMAN	Homo sapiens (Human)
luteolin	P03372	ESR1_HUMAN	Homo sapiens (Human)
luteolin	P24941	CDK2_HUMAN	Homo sapiens (Human)
luteolin	P06276	CHLE_HUMAN	Homo sapiens (Human)
luteolin	P06401	PRGR_HUMAN	Homo sapiens (Human)
luteolin	P56817	BACE1_HUMAN	Homo sapiens (Human)
luteolin	P09211	GSTP1_HUMAN	Homo sapiens (Human)
luteolin	P00491	PNPH_HUMAN	Homo sapiens (Human)
luteolin	P20248	CCNA2_HUMAN	Homo sapiens (Human)
luteolin	P11309	PIM1_HUMAN	Homo sapiens (Human)
luteolin	P00390	GSHR_HUMAN	Homo sapiens (Human)
luteolin	P48736	PK3CG_HUMAN	Homo sapiens (Human)
luteolin	P00533	EGFR_HUMAN	Homo sapiens (Human)
luteolin	Q12884	SEPR_HUMAN	Homo sapiens (Human)
luteolin	P08842	STS_HUMAN	Homo sapiens (Human)
luteolin	P02652	APOA2_HUMAN	Homo sapiens (Human)
luteolin	P00751	CFAB_HUMAN	Homo sapiens (Human)
luteolin	P02768	ALBU_HUMAN	Homo sapiens (Human)
luteolin	P02766	TTHY_HUMAN	Homo sapiens (Human)
luteolin	Q00534	CDK6_HUMAN	Homo sapiens (Human)
luteolin	P53779	MK10_HUMAN	Homo sapiens (Human)
luteolin	P45983	MK08_HUMAN	Homo sapiens (Human)
luteolin	P35968	VGFR2_HUMAN	Homo sapiens (Human)
luteolin	P04062	GLCM_HUMAN	Homo sapiens (Human)
luteolin	O14965	AURKA_HUMAN	Homo sapiens (Human)
luteolin	P62508	ERR3_HUMAN	Homo sapiens (Human)
luteolin	P14061	DHB1_HUMAN	Homo sapiens (Human)
luteolin	O14757	CHK1_HUMAN	Homo sapiens (Human)
luteolin	P54760	EPHB4_HUMAN	Homo sapiens (Human)
luteolin	P04745	AMY1_HUMAN	Homo sapiens (Human)
luteolin	P29474	NOS3_HUMAN	Homo sapiens (Human)
luteolin	P80188	NGAL_HUMAN	Homo sapiens (Human)

luteolin	O15382	BCAT2_HUMAN	Homo sapiens (Human)
luteolin	P03950	ANGI_HUMAN	Homo sapiens (Human)
luteolin	P00439	PH4H_HUMAN	Homo sapiens (Human)
luteolin	P78536	ADA17_HUMAN	Homo sapiens (Human)
luteolin	P16152	CBR1_HUMAN	Homo sapiens (Human)
luteolin	Q00796	DHSO_HUMAN	Homo sapiens (Human)
luteolin	P27338	AOFB_HUMAN	Homo sapiens (Human)
luteolin	P11142	HSP7C_HUMAN	Homo sapiens (Human)
luteolin	P78368	KC1G2_HUMAN	Homo sapiens (Human)
luteolin	P14174	MIF_HUMAN	Homo sapiens (Human)
luteolin	Q16828	DUS6_HUMAN	Homo sapiens (Human)
luteolin	P06737	PYGL_HUMAN	Homo sapiens (Human)
luteolin	P04278	SHBG_HUMAN	Homo sapiens (Human)
luteolin	P15559	NQO1_HUMAN	Homo sapiens (Human)
luteolin	P18075	BMP7_HUMAN	Homo sapiens (Human)
luteolin	Q08499	PDE4D_HUMAN	Homo sapiens (Human)
luteolin	Q13126	MTAP_HUMAN	Homo sapiens (Human)
luteolin	P19793	RXRA_HUMAN	Homo sapiens (Human)
luteolin	P15121	ALDR_HUMAN	Homo sapiens (Human)
luteolin	P45452	MMP13_HUMAN	Homo sapiens (Human)
luteolin	P11766	ADHX_HUMAN	Homo sapiens (Human)
luteolin	O15496	PA2GX_HUMAN	Homo sapiens (Human)
luteolin	P08254	MMP3_HUMAN	Homo sapiens (Human)
luteolin	P23141	EST1_HUMAN	Homo sapiens (Human)
luteolin	Q06520	ST2A1_HUMAN	Homo sapiens (Human)
luteolin	O15530	PDPK1_HUMAN	Homo sapiens (Human)
luteolin	P17707	DCAM_HUMAN	Homo sapiens (Human)
luteolin	P31749	AKT1_HUMAN	Homo sapiens (Human)
luteolin	P12931	SRC_HUMAN	Homo sapiens (Human)
luteolin	P02774	VTDB_HUMAN	Homo sapiens (Human)
luteolin	P00374	DYR_HUMAN	Homo sapiens (Human)
luteolin	P07900	HS90A_HUMAN	Homo sapiens (Human)
luteolin	P28845	DHI1_HUMAN	Homo sapiens (Human)
luteolin	P00742	FA10_HUMAN	Homo sapiens (Human)
luteolin	Q16539	MK14_HUMAN	Homo sapiens (Human)
luteolin	P00746	CFAD_HUMAN	Homo sapiens (Human)
luteolin	P49354	FNTA_HUMAN	Homo sapiens (Human)
luteolin	Q03181	PPARD_HUMAN	Homo sapiens (Human)
luteolin	P08235	MCR_HUMAN	Homo sapiens (Human)
luteolin	P52732	KIF11_HUMAN	Homo sapiens (Human)
luteolin	P35558	PCKGC_HUMAN	Homo sapiens (Human)
luteolin	P04818	TYSY_HUMAN	Homo sapiens (Human)
luteolin	P54577	SYYC_HUMAN	Homo sapiens (Human)
luteolin	Q02127	PYRD_HUMAN	Homo sapiens (Human)
luteolin	Q9NPH5	NOX4_HUMAN	Homo sapiens (Human)
luteolin	Q15078	CD5R1_HUMAN	Homo sapiens (Human)
luteolin	Q00535	CDK5_HUMAN	Homo sapiens (Human)
luteolin	P47989	XDH_HUMAN	Homo sapiens (Human)
luteolin	P21397	AOFA_HUMAN	Homo sapiens (Human)
luteolin	P36888	FLT3_HUMAN	Homo sapiens (Human)
luteolin	Q8WWL7	CCNB3_HUMAN	Homo sapiens (Human)
luteolin	P06493	CDK1_HUMAN	Homo sapiens (Human)
luteolin	P14635	CCNB1_HUMAN	Homo sapiens (Human)
luteolin	O95067	CCNB2_HUMAN	Homo sapiens (Human)
luteolin	P09917	LOX5_HUMAN	Homo sapiens (Human)
luteolin	P30542	AA1R_HUMAN	Homo sapiens (Human)
luteolin	P43166	CAH7_HUMAN	Homo sapiens (Human)
luteolin	Q04760	LGUL_HUMAN	Homo sapiens (Human)

luteolin	P05067	A4_HUMAN	Homo sapiens (Human)
luteolin	P43405	KSYK_HUMAN	Homo sapiens (Human)
luteolin	P49841	GSK3B_HUMAN	Homo sapiens (Human)
luteolin	P09874	PARP1_HUMAN	Homo sapiens (Human)
luteolin	P14780	MMP9_HUMAN	Homo sapiens (Human)
luteolin	O43570	CAH12_HUMAN	Homo sapiens (Human)
luteolin	P08253	MMP2_HUMAN	Homo sapiens (Human)
luteolin	P22748	CAH4_HUMAN	Homo sapiens (Human)
luteolin	P39900	MMP12_HUMAN	Homo sapiens (Human)
luteolin	P28907	CD38_HUMAN	Homo sapiens (Human)
luteolin	Q16678	CP1B1_HUMAN	Homo sapiens (Human)
luteolin	Q9UNQ0	ABCG2_HUMAN	Homo sapiens (Human)
luteolin	O60218	AK1BA_HUMAN	Homo sapiens (Human)
luteolin	Q9H2K2	TNKS2_HUMAN	Homo sapiens (Human)
luteolin	O95271	TNKS1_HUMAN	Homo sapiens (Human)
luteolin	P11387	TOP1_HUMAN	Homo sapiens (Human)
luteolin	P05089	ARGI1_HUMAN	Homo sapiens (Human)
luteolin	Q13332	PTPRS_HUMAN	Homo sapiens (Human)
luteolin	P33527	MRP1_HUMAN	Homo sapiens (Human)
luteolin	P22303	ACES_HUMAN	Homo sapiens (Human)
quercetin	Q9NPH5	NOX4_HUMAN	Homo sapiens (Human)
quercetin	P30518	V2R_HUMAN	Homo sapiens (Human)
quercetin	P15121	ALDR_HUMAN	Homo sapiens (Human)
quercetin	P47989	XDH_HUMAN	Homo sapiens (Human)
quercetin	P21397	AOFA_HUMAN	Homo sapiens (Human)
quercetin	P08069	IGF1R_HUMAN	Homo sapiens (Human)
quercetin	P36888	FLT3_HUMAN	Homo sapiens (Human)
quercetin	P11511	CP19A_HUMAN	Homo sapiens (Human)
quercetin	P00533	EGFR_HUMAN	Homo sapiens (Human)
quercetin	P00734	THRB_HUMAN	Homo sapiens (Human)
quercetin	P00918	CAH2_HUMAN	Homo sapiens (Human)
quercetin	P11309	PIM1_HUMAN	Homo sapiens (Human)
quercetin	P09917	LOX5_HUMAN	Homo sapiens (Human)
quercetin	Q96GD4	AURKB_HUMAN	Homo sapiens (Human)
quercetin	P21917	DRD4_HUMAN	Homo sapiens (Human)
quercetin	P30542	AA1R_HUMAN	Homo sapiens (Human)
quercetin	P43166	CAH7_HUMAN	Homo sapiens (Human)
quercetin	Q04760	LGUL_HUMAN	Homo sapiens (Human)
quercetin	P05164	PERM_HUMAN	Homo sapiens (Human)
quercetin	P27986	P85A_HUMAN	Homo sapiens (Human)
quercetin	P29274	AA2AR_HUMAN	Homo sapiens (Human)
quercetin	P53355	DAPK1_HUMAN	Homo sapiens (Human)
quercetin	P06737	PYGL_HUMAN	Homo sapiens (Human)
quercetin	P00915	CAH1_HUMAN	Homo sapiens (Human)
quercetin	P49841	GSK3B_HUMAN	Homo sapiens (Human)
quercetin	P12931	SRC_HUMAN	Homo sapiens (Human)
quercetin	Q05397	FAK1_HUMAN	Homo sapiens (Human)
quercetin	P37059	DHB2_HUMAN	Homo sapiens (Human)
quercetin	P35968	VGFR2_HUMAN	Homo sapiens (Human)
quercetin	P45452	MMP13_HUMAN	Homo sapiens (Human)
quercetin	P08254	MMP3_HUMAN	Homo sapiens (Human)
quercetin	P07451	CAH3_HUMAN	Homo sapiens (Human)
quercetin	P16050	LOX15_HUMAN	Homo sapiens (Human)
quercetin	P33527	MRP1_HUMAN	Homo sapiens (Human)
quercetin	P53350	PLK1_HUMAN	Homo sapiens (Human)
quercetin	P23280	CAH6_HUMAN	Homo sapiens (Human)
quercetin	P06493	CDK1_HUMAN	Homo sapiens (Human)
quercetin	P14780	MMP9_HUMAN	Homo sapiens (Human)

quercetin	O43570	CAH12_HUMAN	Homo sapiens (Human)
quercetin	P08253	MMP2_HUMAN	Homo sapiens (Human)
quercetin	Q16512	PKN1_HUMAN	Homo sapiens (Human)
quercetin	Q9ULX7	CAH14_HUMAN	Homo sapiens (Human)
quercetin	Q16790	CAH9_HUMAN	Homo sapiens (Human)
quercetin	P68400	CSK21_HUMAN	Homo sapiens (Human)
quercetin	P18054	LOX12_HUMAN	Homo sapiens (Human)
quercetin	P08581	MET_HUMAN	Homo sapiens (Human)
quercetin	P22748	CAH4_HUMAN	Homo sapiens (Human)
quercetin	P51955	NEK2_HUMAN	Homo sapiens (Human)
quercetin	P25024	CXCR1_HUMAN	Homo sapiens (Human)
quercetin	Q13554	KCC2B_HUMAN	Homo sapiens (Human)
quercetin	Q9UM73	ALK_HUMAN	Homo sapiens (Human)
quercetin	P31749	AKT1_HUMAN	Homo sapiens (Human)
quercetin	P08183	MDR1_HUMAN	Homo sapiens (Human)
quercetin	Q9HC98	NEK6_HUMAN	Homo sapiens (Human)
quercetin	P04054	PA21B_HUMAN	Homo sapiens (Human)
quercetin	P35218	CAH5A_HUMAN	Homo sapiens (Human)
quercetin	P56817	BACE1_HUMAN	Homo sapiens (Human)
quercetin	Q16678	CP1B1_HUMAN	Homo sapiens (Human)
quercetin	P30530	UFO_HUMAN	Homo sapiens (Human)
quercetin	Q9UNQ0	ABCG2_HUMAN	Homo sapiens (Human)
quercetin	O60285	NUAK1_HUMAN	Homo sapiens (Human)
quercetin	P52895	AK1C2_HUMAN	Homo sapiens (Human)
quercetin	Q04828	AK1C1_HUMAN	Homo sapiens (Human)
quercetin	P42330	AK1C3_HUMAN	Homo sapiens (Human)
quercetin	P17516	AK1C4_HUMAN	Homo sapiens (Human)
quercetin	Q8N1Q1	CAH13_HUMAN	Homo sapiens (Human)
quercetin	P14550	AK1A1_HUMAN	Homo sapiens (Human)
quercetin	Q9HC97	GPR35_HUMAN	Homo sapiens (Human)
quercetin	P43405	KSYK_HUMAN	Homo sapiens (Human)
quercetin	P10636	TAU_HUMAN	Homo sapiens (Human)
quercetin	B2RXH2	KDM4E_HUMAN	Homo sapiens (Human)
quercetin	P11388	TOP2A_HUMAN	Homo sapiens (Human)
quercetin	P06213	INSR_HUMAN	Homo sapiens (Human)
quercetin	P22303	ACES_HUMAN	Homo sapiens (Human)
quercetin	Q15746	MYLK_HUMAN	Homo sapiens (Human)
quercetin	P48736	PK3CG_HUMAN	Homo sapiens (Human)
quercetin	P27695	APEX1_HUMAN	Homo sapiens (Human)
quercetin	P08631	HCK_HUMAN	Homo sapiens (Human)
quercetin	P03372	ESR1_HUMAN	Homo sapiens (Human)
quercetin	P00751	CFAB_HUMAN	Homo sapiens (Human)
quercetin	P06276	CHLE_HUMAN	Homo sapiens (Human)
quercetin	P06401	PRGR_HUMAN	Homo sapiens (Human)
quercetin	P62937	PPIA_HUMAN	Homo sapiens (Human)
quercetin	P53779	MK10_HUMAN	Homo sapiens (Human)
quercetin	P45983	MK08_HUMAN	Homo sapiens (Human)
quercetin	Q00534	CDK6_HUMAN	Homo sapiens (Human)
quercetin	P04062	GLCM_HUMAN	Homo sapiens (Human)
quercetin	Q12884	SEPR_HUMAN	Homo sapiens (Human)
quercetin	P02766	TTHY_HUMAN	Homo sapiens (Human)
quercetin	P09211	GSTP1_HUMAN	Homo sapiens (Human)
quercetin	P24941	CDK2_HUMAN	Homo sapiens (Human)
quercetin	P26196	DDX6_HUMAN	Homo sapiens (Human)
quercetin	P00491	PNPH_HUMAN	Homo sapiens (Human)
quercetin	Q9NP99	TREM1_HUMAN	Homo sapiens (Human)
quercetin	P00390	GSHR_HUMAN	Homo sapiens (Human)
quercetin	P20248	CCNA2_HUMAN	Homo sapiens (Human)

quercetin	O14965	AURKA_HUMAN	Homo sapiens (Human)
quercetin	P10275	ANDR_HUMAN	Homo sapiens (Human)
quercetin	P55055	NR1H2_HUMAN	Homo sapiens (Human)
quercetin	O14757	CHK1_HUMAN	Homo sapiens (Human)
quercetin	P80188	NGAL_HUMAN	Homo sapiens (Human)
quercetin	P03950	ANGI_HUMAN	Homo sapiens (Human)
quercetin	O15382	BCAT2_HUMAN	Homo sapiens (Human)
quercetin	P00439	PH4H_HUMAN	Homo sapiens (Human)
quercetin	P11142	HSP7C_HUMAN	Homo sapiens (Human)
quercetin	P04745	AMY1_HUMAN	Homo sapiens (Human)
quercetin	P27338	AOFB_HUMAN	Homo sapiens (Human)
quercetin	Q15078	CD5R1_HUMAN	Homo sapiens (Human)
quercetin	P16152	CBR1_HUMAN	Homo sapiens (Human)
quercetin	P78368	KC1G2_HUMAN	Homo sapiens (Human)
quercetin	P04278	SHBG_HUMAN	Homo sapiens (Human)
quercetin	O15530	PDPK1_HUMAN	Homo sapiens (Human)
quercetin	P00736	C1R_HUMAN	Homo sapiens (Human)
quercetin	Q00796	DHSO_HUMAN	Homo sapiens (Human)
quercetin	P14061	DHB1_HUMAN	Homo sapiens (Human)
quercetin	P53582	MAP11_HUMAN	Homo sapiens (Human)
quercetin	Q13126	MTAP_HUMAN	Homo sapiens (Human)
quercetin	P15559	NQO1_HUMAN	Homo sapiens (Human)
quercetin	Q16539	MK14_HUMAN	Homo sapiens (Human)
quercetin	P18075	BMP7_HUMAN	Homo sapiens (Human)
quercetin	P78536	ADA17_HUMAN	Homo sapiens (Human)
quercetin	O15496	PA2GX_HUMAN	Homo sapiens (Human)
quercetin	P54760	EPHB4_HUMAN	Homo sapiens (Human)
quercetin	Q15382	RHEB_HUMAN	Homo sapiens (Human)
quercetin	P07858	CATB_HUMAN	Homo sapiens (Human)
quercetin	P17707	DCAM_HUMAN	Homo sapiens (Human)
quercetin	P62508	ERR3_HUMAN	Homo sapiens (Human)
quercetin	P00374	DYR_HUMAN	Homo sapiens (Human)
quercetin	P09960	LKHA4_HUMAN	Homo sapiens (Human)
quercetin	P27707	DCK_HUMAN	Homo sapiens (Human)
quercetin	P55210	CASP7_HUMAN	Homo sapiens (Human)
quercetin	Q08499	PDE4D_HUMAN	Homo sapiens (Human)
quercetin	P19971	TYPH_HUMAN	Homo sapiens (Human)
quercetin	P00746	CFAD_HUMAN	Homo sapiens (Human)
quercetin	P00797	RENI_HUMAN	Homo sapiens (Human)
quercetin	P49354	FNTA_HUMAN	Homo sapiens (Human)
quercetin	Q03181	PPARD_HUMAN	Homo sapiens (Human)
quercetin	P28845	DHI1_HUMAN	Homo sapiens (Human)
quercetin	P14174	MIF_HUMAN	Homo sapiens (Human)
quercetin	P11362	FGFR1_HUMAN	Homo sapiens (Human)
3'-O-Methylorobol	P00918	CAH2_HUMAN	Homo sapiens (Human)
3'-O-Methylorobol	Q92731	ESR2_HUMAN	Homo sapiens (Human)
3'-O-Methylorobol	P03372	ESR1_HUMAN	Homo sapiens (Human)
3'-O-Methylorobol	P08254	MMP3_HUMAN	Homo sapiens (Human)
3'-O-Methylorobol	P11309	PIM1_HUMAN	Homo sapiens (Human)
3'-O-Methylorobol	P00915	CAH1_HUMAN	Homo sapiens (Human)
3'-O-Methylorobol	P06276	CHLE_HUMAN	Homo sapiens (Human)
3'-O-Methylorobol	P35968	VGFR2_HUMAN	Homo sapiens (Human)
3'-O-Methylorobol	P53779	MK10_HUMAN	Homo sapiens (Human)
3'-O-Methylorobol	P00491	PNPH_HUMAN	Homo sapiens (Human)
3'-O-Methylorobol	Q16539	MK14_HUMAN	Homo sapiens (Human)
3'-O-Methylorobol	P00533	EGFR_HUMAN	Homo sapiens (Human)
3'-O-Methylorobol	P23141	EST1_HUMAN	Homo sapiens (Human)
3'-O-Methylorobol	P10275	ANDR_HUMAN	Homo sapiens (Human)

3'-O-Methylorobol	P04062	GLCM_HUMAN	Homo sapiens (Human)
3'-O-Methylorobol	Q07343	PDE4B_HUMAN	Homo sapiens (Human)
3'-O-Methylorobol	P11511	CP19A_HUMAN	Homo sapiens (Human)
3'-O-Methylorobol	P45983	MK08_HUMAN	Homo sapiens (Human)
3'-O-Methylorobol	O14757	CHK1_HUMAN	Homo sapiens (Human)
3'-O-Methylorobol	P24941	CDK2_HUMAN	Homo sapiens (Human)
3'-O-Methylorobol	P07339	CATD_HUMAN	Homo sapiens (Human)
3'-O-Methylorobol	P00751	CFAB_HUMAN	Homo sapiens (Human)
3'-O-Methylorobol	P02768	ALBU_HUMAN	Homo sapiens (Human)
3'-O-Methylorobol	P08842	STS_HUMAN	Homo sapiens (Human)
3'-O-Methylorobol	P02652	APOA2_HUMAN	Homo sapiens (Human)
3'-O-Methylorobol	P11362	FGFR1_HUMAN	Homo sapiens (Human)
3'-O-Methylorobol	P78536	ADA17_HUMAN	Homo sapiens (Human)
3'-O-Methylorobol	P06401	PRGR_HUMAN	Homo sapiens (Human)
3'-O-Methylorobol	P80188	NGAL_HUMAN	Homo sapiens (Human)
3'-O-Methylorobol	P45452	MMP13_HUMAN	Homo sapiens (Human)
3'-O-Methylorobol	P56817	BACE1_HUMAN	Homo sapiens (Human)
3'-O-Methylorobol	P04278	SHBG_HUMAN	Homo sapiens (Human)
3'-O-Methylorobol	O15382	BCAT2_HUMAN	Homo sapiens (Human)
3'-O-Methylorobol	P11142	HSP7C_HUMAN	Homo sapiens (Human)
3'-O-Methylorobol	P27338	AOFB_HUMAN	Homo sapiens (Human)
3'-O-Methylorobol	P18075	BMP7_HUMAN	Homo sapiens (Human)
3'-O-Methylorobol	Q08499	PDE4D_HUMAN	Homo sapiens (Human)
3'-O-Methylorobol	Q16828	DUS6_HUMAN	Homo sapiens (Human)
3'-O-Methylorobol	P19793	RXRA_HUMAN	Homo sapiens (Human)
3'-O-Methylorobol	P14174	MIF_HUMAN	Homo sapiens (Human)
3'-O-Methylorobol	P00734	THRB_HUMAN	Homo sapiens (Human)
3'-O-Methylorobol	P06737	PYGL_HUMAN	Homo sapiens (Human)
3'-O-Methylorobol	P09960	LKHA4_HUMAN	Homo sapiens (Human)
3'-O-Methylorobol	P49354	FNTA_HUMAN	Homo sapiens (Human)
3'-O-Methylorobol	P03950	ANGI_HUMAN	Homo sapiens (Human)
3'-O-Methylorobol	P22894	MMP8_HUMAN	Homo sapiens (Human)
3'-O-Methylorobol	P00374	DYR_HUMAN	Homo sapiens (Human)
3'-O-Methylorobol	P00390	GSHR_HUMAN	Homo sapiens (Human)
3'-O-Methylorobol	P62508	ERR3_HUMAN	Homo sapiens (Human)
3'-O-Methylorobol	Q00534	CDK6_HUMAN	Homo sapiens (Human)
3'-O-Methylorobol	P04745	AMY1_HUMAN	Homo sapiens (Human)
3'-O-Methylorobol	P50579	MAP2_HUMAN	Homo sapiens (Human)
3'-O-Methylorobol	P31749	AKT1_HUMAN	Homo sapiens (Human)
3'-O-Methylorobol	P27487	DPP4_HUMAN	Homo sapiens (Human)
3'-O-Methylorobol	P07858	CATB_HUMAN	Homo sapiens (Human)
3'-O-Methylorobol	P14061	DHB1_HUMAN	Homo sapiens (Human)
3'-O-Methylorobol	P28845	DHI1_HUMAN	Homo sapiens (Human)
3'-O-Methylorobol	P15121	ALDR_HUMAN	Homo sapiens (Human)
3'-O-Methylorobol	P07900	HS90A_HUMAN	Homo sapiens (Human)
3'-O-Methylorobol	P00742	FA10_HUMAN	Homo sapiens (Human)
3'-O-Methylorobol	Q03181	PPARD_HUMAN	Homo sapiens (Human)
3'-O-Methylorobol	P29474	NOS3_HUMAN	Homo sapiens (Human)
3'-O-Methylorobol	Q13370	PDE3B_HUMAN	Homo sapiens (Human)
3'-O-Methylorobol	P54760	EPHB4_HUMAN	Homo sapiens (Human)
3'-O-Methylorobol	P52732	KIF11_HUMAN	Homo sapiens (Human)
3'-O-Methylorobol	Q02127	PYRD_HUMAN	Homo sapiens (Human)
3'-O-Methylorobol	P78368	KC1G2_HUMAN	Homo sapiens (Human)
3'-O-Methylorobol	Q06520	ST2A1_HUMAN	Homo sapiens (Human)
3'-O-Methylorobol	P53350	PLK1_HUMAN	Homo sapiens (Human)
3'-O-Methylorobol	P35558	PCKGC_HUMAN	Homo sapiens (Human)
3'-O-Methylorobol	P02774	VTDB_HUMAN	Homo sapiens (Human)
3'-O-Methylorobol	P20248	CCNA2_HUMAN	Homo sapiens (Human)

3'-O-Methylorobol	Q13126	MTAP_HUMAN	Homo sapiens (Human)
3'-O-Methylorobol	O76074	PDE5A_HUMAN	Homo sapiens (Human)
3'-O-Methylorobol	O15530	PDPK1_HUMAN	Homo sapiens (Human)
3'-O-Methylorobol	P04818	TYSY_HUMAN	Homo sapiens (Human)
3'-O-Methylorobol	P17707	DCAM_HUMAN	Homo sapiens (Human)

Targets of chemicals after removing the overlap	
Entry	Gene names
P52895	AKR1C2
P27338	MAOB
P11309	PIM1
P12643	BMP2
P08842	STS
P49137	MAPKAPK2
P02652	APOA2
P55210	CASP7
P10828	THRB
P00918	CA2
P02768	ALB
P28482	MAPK1
P06401	PGR
P08235	NR3C2
P14061	HSD17B1
P45452	MMP13
P52732	KIF11
P35398	RORA
O76054	SEC14L2
P02774	GC
P03372	ESR1
Q06520	SULT2A1
P20701	ITGAL
P78536	ADAM17
Q16539	MAPK14
P45983	MAPK8
P28845	HSD11B1
P02766	TTR
P24941	CDK2
P10275	AR
P12931	SRC
Q07343	PDE4B
P00533	EGFR
Q96RI1	NR1H4
P0DP23	CALM1
P0DP24	CALM2
P0DP25	CALM3
O15530	PDPK1
P63316	TNNC1
P00734	F2
P56817	BACE1
Q06124	PTPN11
P11086	PNMT
P53041	PPP5C
P36897	TGFBR1
P04278	SHBG
P15056	BRAF
P00915	CA1
P23141	CES1

P15121	AKR1B1
P08758	ANXA5
P35968	KDR
O14757	CHEK1
P37231	PPARG
P27487	DPP4
Q16828	DUSP6
O43617	TRAPPC3
P49638	TTPA
O60760	HPGDS
P50579	METAP2
P04818	TYMS
Q02127	DHODH
Q14994	NR1I3
P08263	GSTA1
P00742	F10
P06276	BCHE
P55263	ADK
P42768	WAS
Q9BY41	HDAC8
P08069	IGF1R
Q13126	MTAP
Q04828	AKR1C1
O00204	SULT2B1
P02753	RBP4
P29474	NOS3
P43405	SYK
Q13133	NR1H3
P19793	RXRA
P54760	EPHB4
P11474	ESRRA
P15559	NQO1
P62508	ESRRG
P55055	NR1H2
P00326	ADH1C
P04035	HMGCR
Q16850	CYP51A1
Q9NPH5	NOX4
P47989	XDH
P14679	TYR
P36888	FLT3
P09917	ALOX5
P43166	CA7
P37059	HSD17B2
P33527	ABCC1
P35869	AHR
O43570	CA12
P08183	ABCB1
Q16678	CYP1B1
Q9UNQ0	ABCG2
P30542	ADORA1
P22748	CA4
P22303	ACHE
P21397	MAOA
Q04760	GLO1
P49841	GSK3B
P14780	MMP9
P08253	MMP2

P16050	ALOX15
P18054	ALOX12
Q13332	PTPRS
P08631	HCK
Q00534	CDK6
P62937	PPIA
P04062	GBA
Q9NP99	TREM1
P00751	CFB
P00390	GSR
P53779	MAPK10
Q15078	CDK5R1
P03950	ANG
O15382	BCAT2
P11142	HSPA8
O14965	AURKA
P00736	C1R
P06737	PYGL
P07858	CTSB
P00491	PNP
Q08499	PDE4D
P27707	DCK
O15496	PLA2G10
P07900	HSP90AA1
P00374	DHFR
P09960	LTA4H
P20248	CCNA2
P08254	MMP3
P35558	PCK1
P39900	MMP12
P11511	CYP19A1
P09211	GSTP1
Q12884	FAP
P48736	PIK3CG
Q16740	CLPP
P25774	CTSS
Q00796	SORD
P00749	PLAU
P80188	LCN2
P00439	PAH
P04745	AMY1A
P78368	CSNK1G2
P16152	CBR1
P17707	AMD1
P17342	NPR3
P47929	LGALS7
P18075	BMP7
P19971	TYMP
P11766	ADH5
Q15382	RHEB
P23526	AHCY
P00746	CFD
P01709	IGLV2-8
P07339	CTSD
Q9BW91	NUDT9
P62942	FKBP1A
O60911	CTSV
P53582	METAP1

P26196	DDX6
P37173	TGFBR2
P23946	CMA1
Q92731	ESR2
Q8NBQ5	HSD17B11
P25311	AZGP1
Q9P2W7	B3GAT1
P19367	HK1
Q05315	CLC
P51570	GALK1
P16083	NQO2
Q9NVS9	PNPO
Q13231	CHIT1
P48449	LSS
P18031	PTPN1
P02743	APCS
P53350	PLK1
P14174	MIF
Q99933	BAG1
P49354	FNTA
P31749	AKT1
P43235	CTSK
P42330	AKR1C3
Q96T66	NMNAT3
P05451	REG1A
P11362	FGFR1
Q13370	PDE3B
P29218	IMPA1
P09874	PARP1
P00325	ADH1B
O76074	PDE5A
P04746	AMY2A
P15090	FABP4
Q96AZ6	ISG20
P23919	DTYMK
P43155	CRAT
P06239	LCK
P49789	FHIT
P11712	CYP2C9
P84077	ARF1
Q03181	PPARD
P00797	REN
P08581	MET
P61586	RHOA
P11586	MTHFD1
P16442	ABO
O43252	PAPSS1
P22894	MMP8
P52333	JAK3
Q08881	ITK
Q07912	TNK2
P34896	SHMT1
P15153	RAC2
P05019	IGF1
P01009	SERPINA1
Q10588	BST1
P05091	ALDH2
P54577	YARS

P34913	EPHX2
Q00987	MDM2
P04179	SOD2
P30043	BLVRB
P21802	FGFR2
Q16773	KYAT1
P00747	PLG
P49366	DHPS
P08709	F7
Q14541	HNF4G
O75469	NR1I2
P08246	ELANE
P49763	PGF
P42574	CASP3
P13598	ICAM2
Q9BZR6	RTN4R
P24071	FCAR
P53355	DAPK1
P07686	HEXB
P35221	CTNNA1
P16109	SELP
Q01469	FABP5
P68400	CSNK2A1
P10827	THRA
P06126	CD1A
P41240	CSK
Q07869	PPARA
Q9P286	PAK5
P32320	CDA
Q16769	QPCT
P09237	MMP7
P12268	IMPDH2
P62166	NCS1
Q07960	ARHGAP1
Q08188	TGM3
Q9BZX2	UCK2
P60568	IL2
P06746	POLB
O60218	AKR1B10
P51449	RORC
P10586	PTPRF
P17706	PTPN2
P24666	ACP1
P30305	CDC25B
P15309	ACPP
O15540	FABP7
P05162	LGALS2
Q16836	HADH
P60174	TPI1
P62491	RAB11A
P07195	LDHB
P35228	NOS2
P17931	LGALS3
Q00535	CDK5
Q8WWL7	CCNB3
P06493	CDK1
P14635	CCNB1
O95067	CCNB2

P05067	APP
P28907	CD38
Q9H2K2	TNKS2
O95271	TNKS
P11387	TOP1
P05089	ARG1
P30518	AVPR2
Q96GD4	AURKB
P21917	DRD4
P05164	MPO
P27986	PIK3R1
P29274	ADORA2A
Q05397	PTK2
P07451	CA3
P23280	CA6
Q16512	PKN1
Q9ULX7	CA14
Q16790	CA9
P51955	NEK2
P25024	CXCR1
Q13554	CAMK2B
Q9UM73	ALK
Q9HC98	NEK6
P04054	PLA2G1B
P35218	CA5A
P30530	AXL
O60285	NUAK1
P17516	AKR1C4
Q8N1Q1	CA13
P14550	AKR1A1
Q9HC97	GPR35
P10636	MAPT
B2RXH2	KDM4E
P11388	TOP2A
P06213	INSR
Q15746	MYLK
P27695	APEX1
