# Supplementary material for: Systematic Elucidation of the Potential Mechanism of Erzhi Pill against Drug-Induced Liver Injury via Network Pharmacology Approach
Source: Evid Based Complement Alternat Med. 2020 Jan 6;2020:6219432. doi: 10.1155/2020/6219432 (PMC6970004; doi:10.1155/2020/6219432)
Supplement: https://www.uniprot.org/ Supplementary Materials — Table S1: putative targets for chemicals contained in EZP. Chemname was obtained from TCMSP. Entry, Entry name, and Protein name were obtained from PharmMapper. Entry was converted to Gene name by Uniprot. (https://www.uniprot.org/) Table S2: targets related to DILI. Detailed information of targets related to DILI, there were 267 targets from DrugBank and 357 targets from GeneCards with 42 targets duplicated. Table S3: common targets related to bioactive compounds. Detailed information of 89 common targets related to 23 bioactive compounds. Table S4: the results of GO and KEGG pathway enrichment. 223 BPs, 25 CCs, 65 MFs enriched have a P value less than 0.05, and 86 KEGG pathways were recognized as P < 0.05. [file 6219432.f1.zip › 6219432.f1/Supplementary Table S2.pdf]

**Title: Uncovering the pharmacological mechanism of Erzhi pill on drug-induced liver injury based on network pharmacology**

## Table S2 Targets related to DILI

| Targets from DrugBank | Targets from GeneCards |
|---|---|
| P21554 | P01375 |
| Q15822 | P05231 |
| Q05901 | P04637 |
| P10632 | P01308 |
| Q86VL8 | P01584 |
| Q2M3G0 | P01579 |
| P08183 | P00734 |
| P31948 | P22301 |
| P00387 | P01137 |
| P06737 | P25445 |
| Q12882 | P42574 |
| Q9UHQ9 | P02768 |
| P23141 | P35228 |
| P04275 | Q14790 |
| Q96RQ9 | P00533 |
| P07148 | P10145 |
| Q9UI32 | P05362 |
| P49788 | P24298 |
| P41743 | P10635 |
| P33261 | P48023 |
| P30041 | P08183 |
| P11498 | P11712 |
| Q8NFJ5 | P35354 |
| P39900 | P13500 |
| P05089 | P15692 |
| Q9Y694 | P02649 |
| P04080 | P04275 |
| Q13867 | Q9TQE0 |
| P50416 | P12821 |
| P11168 | P28482 |
| Q06520 | P08684 |
| P01308 | P01106 |
| P02753 | P30491 |
| P05186 | P33261 |
| P55789 | P05164 |
| P98066 | P35222 |
| P09467 | P37231 |
| O95255 | P31749 |
| P24298 | Q16665 |
| Q9H222 | P29965 |
| Q9H221 | P05181 |
| P05177 | P01589 |
| P41235 | P24385 |
| P04798 | P10415 |
| P33260 | P12259 |
| Q96SL4 | Q30201 |
| P13584 | P60568 |
| O43272 | P45983 |
| Q13133 | Q9NRA2 |
| P20815 | P41159 |

| Total targets after eliminating the redundancy | |
|---|---|
| Entry | Gene names |
| P21554 | CNR1 |
| Q15822 | CHRNA2 |
| Q05901 | CHRNB3 |
| P10632 | CYP2C8 |
| Q86VL8 | SLC47A2 |
| Q2M3G0 | ABCB5 |
| P08183 | ABCB1 |
| P31948 | STIP1 |
| P00387 | CYB5R3 |
| P06737 | PYGL |
| Q12882 | DPYD |
| Q9UHQ9 | CYB5R1 |
| P23141 | CES1 |
| P04275 | VWF |
| Q96RQ9 | IL4I1 |
| P07148 | FABP1 |
| Q9UI32 | GLS2 |
| P49788 | RARRES1 |
| P41743 | PRKCI |
| P33261 | CYP2C19 |
| P30041 | PRDX6 |
| P11498 | PC |
| Q8NFJ5 | GPRC5A |
| P39900 | MMP12 |
| P05089 | ARG1 |
| Q9Y694 | SLC22A7 |
| P04080 | CSTB |
| Q13867 | BLMH |
| P50416 | CPT1A |
| P11168 | SLC2A2 |
| Q06520 | SULT2A1 |
| P01308 | INS |
| P02753 | RBP4 |
| P05186 | ALPL |
| P55789 | GFER |
| P98066 | TNFAIP6 |
| P09467 | FBP1 |
| O95255 | ABCC6 |
| P24298 | GPT |
| Q9H222 | ABCG5 |
| Q9H221 | ABCG8 |
| P05177 | CYP1A2 |
| P41235 | HNF4A |
| P04798 | CYP1A1 |
| P33260 | CYP2C18 |
| Q96SL4 | GPX7 |
| P13584 | CYP4B1 |
| O43272 | PRODH |
| Q13133 | NR1H3 |

| | |
|---|---|
| P24462 | P16410 |
| Q9NPD5 | P09601 |
| P02647 | P05121 |
| P49638 | P01133 |
| P23381 | P14780 |
| Q9Y6L6 | P60484 |
| P98160 | P19438 |
| P20813 | P02741 |
| P21453 | P02787 |
| O14791 | P42345 |
| P22570 | P11166 |
| O14939 | Q96RI1 |
| Q07820 | P10316 |
| P19525 | P04040 |
| Q16568 | P01138 |
| Q04828 | P14136 |
| P02778 | P14210 |
| Q6NUM9 | P07204 |
| Q14624 | P13726 |
| P30043 | Q15848 |
| Q16881 | P23786 |
| P11712 | P00441 |
| P18825 | P18510 |
| P10176 | P01100 |
| Q15238 | P05112 |
| O95470 | P42771 |
| Q9UHC9 | P10147 |
| Q92570 | P05019 |
| Q9P0J0 | P01588 |
| Q96Q40 | P40763 |
| P55055 | P35225 |
| P16118 | O00206 |
| P13945 | P01583 |
| Q9HD40 | P01009 |
| Q9H0K1 | P04114 |
| Q04760 | P02647 |
| P05107 | P01562 |
| P51692 | P29474 |
| P35557 | P05305 |
| Q14032 | P42336 |
| P51161 | P41235 |
| P07550 | P05177 |
| P08588 | P21964 |
| P30520 | P23560 |
| P28907 | P21817 |
| P08684 | O60674 |
| O14788 | P22309 |
| O75340 | P14416 |
| P29508 | P05412 |
| P30613 | Q07812 |
| P35968 | Q96P20 |
| Q5NUL3 | P00797 |
| P08100 | P11413 |
| P33681 | P99999 |
| Q02156 | P05067 |
| P43353 | P16671 |
| P23469 | P01116 |

| | |
|---|---|
| P20815 | CYP3A5 |
| P24462 | CYP3A7 |
| Q9NPD5 | SLCO1B3 |
| P02647 | APOA1 |
| P49638 | TTPA |
| P23381 | WARS |
| Q9Y6L6 | SLCO1B1 |
| P98160 | HSPG2 |
| P20813 | CYP2B6 |
| P21453 | S1PR1 |
| O14791 | APOL1 |
| P22570 | FDXR |
| O14939 | PLD2 |
| Q07820 | MCL1 |
| P19525 | EIF2AK2 |
| Q16568 | CARTPT |
| Q04828 | AKR1C1 |
| P02778 | CXCL10 |
| Q6NUM9 | RETSAT |
| Q14624 | ITIH4 |
| P30043 | BLVRB |
| Q16881 | TXNRD1 |
| P11712 | CYP2C9 |
| P18825 | ADRA2C |
| P10176 | COX8A |
| Q15238 | PSG5 |
| O95470 | SGPL1 |
| Q9UHC9 | NPC1L1 |
| Q92570 | NR4A3 |
| Q9P0J0 | NDUFA13 |
| Q96Q40 | CDK15 |
| P55055 | NR1H2 |
| P16118 | PFKFB1 |
| P13945 | ADRB3 |
| Q9HD40 | SEPSECS |
| Q9H0K1 | SIK2 |
| Q04760 | GLO1 |
| P05107 | ITGB2 |
| P51692 | STAT5B |
| P35557 | GCK |
| Q14032 | BAAT |
| P51161 | FABP6 |
| P07550 | ADRB2 |
| P08588 | ADRB1 |
| P30520 | ADSS |
| P28907 | CD38 |
| P08684 | CYP3A4 |
| O14788 | TNFSF11 |
| O75340 | PDCD6 |
| P29508 | SERPINB3 |
| P30613 | PKLR |
| P35968 | KDR |
| Q5NUL3 | FFAR4 |
| P08100 | RHO |
| P33681 | CD80 |
| Q02156 | PRKCE |
| P43353 | ALDH3B1 |

| | |
|---|---|
| Q9Y5N1 | P10721 |
| P19235 | P51681 |
| Q96KS0 | P04626 |
| Q9BV57 | P98170 |
| Q16539 | P48061 |
| P27986 | P13569 |
| O00459 | P50591 |
| P29474 | P10636 |
| P08913 | Q07869 |
| P18089 | P11245 |
| P36888 | P40225 |
| P32238 | P27361 |
| P62993 | Q16236 |
| P30044 | O14746 |
| P20333 | P01920 |
| O00748 | P00740 |
| Q15413 | O95342 |
| Q969G6 | Q07817 |
| Q99584 | P22004 |
| Q13464 | P08581 |
| P78348 | P06881 |
| P18031 | P01112 |
| Q14643 | P25054 |
| P31942 | P09488 |
| Q9Y5Y4 | P06858 |
| P03950 | Q16539 |
| Q05655 | P55211 |
| Q99497 | P05113 |
| Q8IU85 | P37840 |
| P01009 | O95477 |
| P07900 | P09038 |
| Q96PY6 | P17813 |
| Q96QT4 | Q14116 |
| P20138 | P09919 |
| O75838 | P01008 |
| P10276 | P40939 |
| O75676 | P27797 |
| Q9UQB9 | Q04206 |
| Q16678 | P19440 |
| Q14721 | P11215 |
| P17252 | P02771 |
| P01023 | P12830 |
| P02775 | P02766 |
| Q9Y2I1 | P01130 |
| Q13163 | P08246 |
| P05771 | P09874 |
| P11142 | P38936 |
| P62745 | P00451 |
| P07814 | P04798 |
| P43026 | P21439 |
| P20273 | P07101 |
| P63000 | P04818 |
| Q96PD7 | P12643 |
| Q8TDS4 | O15455 |
| Q99527 | P09211 |
| P16070 | P42224 |
| P30405 | Q92887 |

| | |
|---|---|
| P23469 | PTPRE |
| Q9Y5N1 | HRH3 |
| P19235 | EPOR |
| Q96KS0 | EGLN2 |
| Q9BV57 | ADI1 |
| Q16539 | MAPK14 |
| P27986 | PIK3R1 |
| O00459 | PIK3R2 |
| P29474 | NOS3 |
| P08913 | ADRA2A |
| P18089 | ADRA2B |
| P36888 | FLT3 |
| P32238 | CCKAR |
| P62993 | GRB2 |
| P30044 | PRDX5 |
| P20333 | TNFRSF1B |
| O00748 | CES2 |
| Q15413 | RYR3 |
| Q969G6 | RFK |
| Q99584 | S100A13 |
| Q13464 | ROCK1 |
| P78348 | ASIC1 |
| P18031 | PTPN1 |
| Q14643 | ITPR1 |
| P31942 | HNRNPH3 |
| Q9Y5Y4 | PTGDR2 |
| P03950 | ANG |
| Q05655 | PRKCD |
| Q99497 | PARK7 |
| Q8IU85 | CAMK1D |
| P01009 | SERPINA1 |
| P07900 | HSP90AA1 |
| Q96PY6 | NEK1 |
| Q96QT4 | TRPM7 |
| P20138 | CD33 |
| O75838 | CIB2 |
| P10276 | RARA |
| O75676 | RPS6KA4 |
| Q9UQB9 | AURKC |
| Q16678 | CYP1B1 |
| Q14721 | KCNB1 |
| P17252 | PRKCA |
| P01023 | A2M |
| P02775 | PPBP |
| Q9Y2I1 | NISCH |
| Q13163 | MAP2K5 |
| P05771 | PRKCB |
| P11142 | HSPA8 |
| P62745 | RHOB |
| P07814 | EPRS |
| P43026 | GDF5 |
| P20273 | CD22 |
| P63000 | RAC1 |
| Q96PD7 | DGAT2 |
| Q8TDS4 | HCAR2 |
| Q99527 | GPER1 |
| P16070 | CD44 |

| | |
|---|---|
| O14786 | P30556 |
| P28062 | P01909 |
| Q15746 | P16109 |
| P09619 | P04035 |
| P48745 | P06213 |
| Q86UE8 | P01344 |
| P43487 | P35968 |
| O15197 | P10451 |
| P61073 | P19838 |
| P05129 | P04141 |
| P01042 | P11532 |
| O15530 | P00742 |
| P51812 | P13501 |
| P53667 | P01127 |
| Q03518 | P11802 |
| O75116 | O75874 |
| Q16513 | P00492 |
| P42680 | P08700 |
| Q9NQU5 | Q9Y2R2 |
| O00206 | P12318 |
| Q9H093 | P54098 |
| Q9Y6E0 | P02778 |
| P04406 | P61278 |
| Q7Z2W7 | P51671 |
| P29317 | P0C0L4 |
| Q07912 | P02545 |
| Q04759 | Q9BZS1 |
| Q15139 | P51580 |
| O14920 | Q16552 |
| Q9HC29 | P16581 |
| O15264 | P01024 |
| Q13131 | P51587 |
| O43293 | P19320 |
| P57059 | Q76LX8 |
| Q9H6Z9 | P08253 |
| P36896 | P28329 |
| P06241 | O75197 |
| P16389 | P04179 |
| Q99828 | P36888 |
| Q13882 | O15553 |
| P16234 | P07900 |
| P45984 | P03372 |
| Q06418 | P15502 |
| P20742 | O60603 |
| O60674 | P05089 |
| P51813 | P03956 |
| Q96GD4 | P48735 |
| Q15418 | P81172 |
| P17612 | P11226 |
| P42338 | Q9UJS0 |
| P35398 | P09104 |
| O60285 | P15559 |
| P51787 | P20813 |
| Q13164 | Q06124 |
| Q9HBH9 | P04271 |
| P52564 | P00738 |
| P49840 | Q8IZI9 |

| | |
|---|---|
| P30405 | PPIF |
| O14786 | NRP1 |
| P28062 | PSMB8 |
| Q15746 | MYLK |
| P09619 | PDGFRB |
| P48745 | CCN3 |
| Q86UE8 | TLK2 |
| P43487 | RANBP1 |
| O15197 | EPHB6 |
| P61073 | CXCR4 |
| P05129 | PRKCG |
| P01042 | KNG1 |
| O15530 | PDPK1 |
| P51812 | RPS6KA3 |
| P53667 | LIMK1 |
| Q03518 | TAP1 |
| O75116 | ROCK2 |
| Q16513 | PKN2 |
| P42680 | TEC |
| Q9NQU5 | PAK6 |
| O00206 | TLR4 |
| Q9H093 | NUAK2 |
| Q9Y6E0 | STK24 |
| P04406 | GAPDH |
| Q7Z2W7 | TRPM8 |
| P29317 | EPHA2 |
| Q07912 | TNK2 |
| Q04759 | PRKCQ |
| Q15139 | PRKD1 |
| O14920 | IKBKB |
| Q9HC29 | NOD2 |
| O15264 | MAPK13 |
| Q13131 | PRKAA1 |
| O43293 | DAPK3 |
| P57059 | SIK1 |
| Q9H6Z9 | EGLN3 |
| P36896 | ACVR1B |
| P06241 | FYN |
| P16389 | KCNA2 |
| Q99828 | CIB1 |
| Q13882 | PTK6 |
| P16234 | PDGFRA |
| P45984 | MAPK9 |
| Q06418 | TYRO3 |
| P20742 | PZP |
| O60674 | JAK2 |
| P51813 | BMX |
| Q96GD4 | AURKB |
| Q15418 | RPS6KA1 |
| P17612 | PRKACA |
| P42338 | PIK3CB |
| P35398 | RORA |
| O60285 | NUAK1 |
| P51787 | KCNQ1 |
| Q13164 | MAPK7 |
| Q9HBH9 | MKNK2 |
| P52564 | MAP2K6 |

| | |
|---|---|
| O00329 | P22303 |
| P05412 | Q03135 |
| Q99829 | P23219 |
| P28482 | P20823 |
| P07332 | O15392 |
| P24941 | Q13315 |
| P19838 | P01042 |
| P29590 | P00747 |
| P36404 | O60260 |
| P00519 | Q13813 |
| Q8IYT8 | P61073 |
| P54764 | P35498 |
| Q15759 | P0C0L5 |
| P10909 | P42898 |
| Q13188 | P01236 |
| Q9H2X6 | P21554 |
| Q8IW41 | P30499 |
| P56537 | P01019 |
| Q13177 | P05787 |
| Q99653 | Q9Y259 |
| Q9HBA0 | P16220 |
| P31749 | Q16667 |
| Q14164 | P35575 |
| Q13153 | P51608 |
| P23246 | P00395 |
| P06702 | P10253 |
| Q9HB55 | P47989 |
| Q05513 | P15976 |
| P35348 | P08670 |
| P35368 | P08575 |
| P25100 | O43520 |
| Q14571 | P09936 |
| Q14573 | P01160 |
| P04839 | P05783 |
| P13498 | P12004 |
| P14598 | P01189 |
| P19878 | P08758 |
| Q15080 | Q7Z6L0 |
| P15153 | P00749 |
| P54646 | P11912 |
| Q9Y478 | P33527 |
| O43741 | P20815 |
| P54619 | Q9UNQ0 |
| Q9UGJ0 | P20674 |
| Q9UGI9 | O75469 |
| Q00653 | P25942 |
| | P34932 |
| | P00750 |
| | P02818 |
| | P01111 |
| | P48357 |
| | Q13158 |
| | Q15116 |
| | P29401 |
| | P12931 |
| | P11509 |
| | O43181 |

| | |
|---|---|
| P49840 | GSK3A |
| O00329 | PIK3CD |
| P05412 | JUN |
| Q99829 | CPNE1 |
| P28482 | MAPK1 |
| P07332 | FES |
| P24941 | CDK2 |
| P19838 | NFKB1 |
| P29590 | PML |
| P36404 | ARL2 |
| P00519 | ABL1 |
| Q8IYT8 | ULK2 |
| P54764 | EPHA4 |
| Q15759 | MAPK11 |
| P10909 | CLU |
| Q13188 | STK3 |
| Q9H2X6 | HIPK2 |
| Q8IW41 | MAPKAPK5 |
| P56537 | EIF6 |
| Q13177 | PAK2 |
| Q99653 | CHP1 |
| Q9HBA0 | TRPV4 |
| P31749 | AKT1 |
| Q14164 | IKBKE |
| Q13153 | PAK1 |
| P23246 | SFPQ |
| P06702 | S100A9 |
| Q9HB55 | CYP3A43 |
| Q05513 | PRKCZ |
| P35348 | ADRA1A |
| P35368 | ADRA1B |
| P25100 | ADRA1D |
| Q14571 | ITPR2 |
| Q14573 | ITPR3 |
| P04839 | CYBB |
| P13498 | CYBA |
| P14598 | NCF1 |
| P19878 | NCF2 |
| Q15080 | NCF4 |
| P15153 | RAC2 |
| P54646 | PRKAA2 |
| Q9Y478 | PRKAB1 |
| O43741 | PRKAB2 |
| P54619 | PRKAG1 |
| Q9UGJ0 | PRKAG2 |
| Q9UGI9 | PRKAG3 |
| Q00653 | NFKB2 |
| P01375 | TNF |
| P05231 | IL6 |
| P04637 | TP53 |
| P01584 | IL1B |
| P01579 | IFNG |
| P00734 | F2 |
| P22301 | IL10 |
| P01137 | TGFB1 |
| P25445 | FAS |
| P42574 | CASP3 |

| |
|---|
| O00443 |
| P21583 |
| P39905 |
| Q9HC29 |
| P28223 |
| Q9BYX4 |
| Q14653 |
| P14778 |
| Q07820 |
| P06276 |
| P02786 |
| P49715 |
| P15056 |
| P04629 |
| P09429 |
| P00390 |
| P09917 |
| P21802 |
| P48736 |
| P09603 |
| Q12882 |
| O14788 |
| P06737 |
| P05187 |
| P55055 |
| P01011 |
| P07550 |
| Q13568 |
| P60033 |
| P01303 |
| O00220 |
| P11473 |
| P04839 |
| Q16620 |
| P29466 |
| P49682 |
| P17693 |
| P46531 |
| P21397 |
| P14222 |
| Q9NST1 |
| P68871 |
| P08709 |
| O14936 |
| Q9BYW2 |
| P08603 |
| P00374 |
| P01033 |
| P35670 |
| P14174 |
| P05106 |
| P04049 |
| P61769 |
| P04150 |
| P20333 |
| P00450 |
| P06850 |

| | |
|---|---|
| P02768 | ALB |
| P35228 | NOS2 |
| Q14790 | CASP8 |
| P00533 | EGFR |
| P10145 | CXCL8 |
| P05362 | ICAM1 |
| P10635 | CYP2D6 |
| P48023 | FASLG |
| P35354 | PTGS2 |
| P13500 | CCL2 |
| P15692 | VEGFA |
| P02649 | APOE |
| Q9TQE0 | HLA-DRB1 |
| P12821 | ACE |
| P01106 | MYC |
| P30491 | HLA-B |
| P05164 | MPO |
| P35222 | CTNNB1 |
| P37231 | PPARG |
| Q16665 | HIF1A |
| P29965 | CD40LG |
| P05181 | CYP2E1 |
| P01589 | IL2RA |
| P24385 | CCND1 |
| P10415 | BCL2 |
| P12259 | F5 |
| Q30201 | HFE |
| P60568 | IL2 |
| P45983 | MAPK8 |
| Q9NRA2 | SLC17A5 |
| P41159 | LEP |
| P16410 | CTLA4 |
| P09601 | HMOX1 |
| P05121 | SERPINE1 |
| P01133 | EGF |
| P14780 | MMP9 |
| P60484 | PTEN |
| P19438 | TNFRSF1A |
| P02741 | CRP |
| P02787 | TF |
| P42345 | MTOR |
| P11166 | SLC2A1 |
| Q96RI1 | NR1H4 |
| P10316 | HLA-A |
| P04040 | CAT |
| P01138 | NGF |
| P14136 | GFAP |
| P14210 | HGF |
| P07204 | THBD |
| P13726 | F3 |
| Q15848 | ADIPOQ |
| P23786 | CPT2 |
| P00441 | SOD1 |
| P18510 | IL1RN |
| P01100 | FOS |
| P05112 | IL4 |
| P42771 | CDKN2A |

| |
|---|
| P29475 |
| O60934 |
| P21757 |
| P16930 |
| Q09428 |
| Q13427 |
| P00813 |
| O14763 |
| Q13224 |
| Q05655 |
| P11362 |
| P38398 |
| Q00987 |
| P17252 |
| P35372 |
| P01574 |
| P19544 |
| Q9HD89 |
| P01266 |
| P15941 |
| Q8NER1 |

| | |
|---|---|
| P10147 | CCL3 |
| P05019 | IGF1 |
| P01588 | EPO |
| P40763 | STAT3 |
| P35225 | IL13 |
| P01583 | IL1A |
| P04114 | APOB |
| P01562 | IFNA1; |
| P05305 | EDN1 |
| P42336 | PIK3CA |
| P21964 | COMT |
| P23560 | BDNF |
| P21817 | RYR1 |
| P22309 | UGT1A1 |
| P14416 | DRD2 |
| Q07812 | BAX |
| Q96P20 | NLRP3 |
| P00797 | REN |
| P11413 | G6PD |
| P99999 | CYCS |
| P05067 | APP |
| P16671 | CD36 |
| P01116 | KRAS |
| P10721 | KIT |
| P51681 | CCR5 |
| P04626 | ERBB2 |
| P98170 | XIAP |
| P48061 | CXCL12 |
| P13569 | CFTR |
| P50591 | TNFSF10 |
| P10636 | MAPT |
| Q07869 | PPARA |
| P11245 | NAT2 |
| P40225 | THPO |
| P27361 | MAPK3 |
| Q16236 | NFE2L2 |
| O14746 | TERT |
| P01920 | HLA-DQB1 |
| P00740 | F9 |
| O95342 | ABCB11 |
| Q07817 | BCL2L1 |
| P22004 | BMP6 |
| P08581 | MET |
| P06881 | CALCA |
| P01112 | HRAS |
| P25054 | APC |
| P09488 | GSTM1 |
| P06858 | LPL |
| P55211 | CASP9 |
| P05113 | IL5 |
| P37840 | SNCA |
| O95477 | ABCA1 |
| P09038 | FGF2 |
| P17813 | ENG |
| Q14116 | IL18 |
| P09919 | CSF3 |
| P01008 | SERPINC1 |
| P40939 | HADHA |

| | |
|---|---|
| P27797 | CALR |
| Q04206 | RELA |
| P19440 | GGT1 |
| P11215 | ITGAM |
| P02771 | AFP |
| P12830 | CDH1 |
| P02766 | TTR |
| P01130 | LDLR |
| P08246 | ELANE |
| P09874 | PARP1 |
| P38936 | CDKN1A |
| P00451 | F8 |
| P21439 | ABCB4 |
| P07101 | TH |
| P04818 | TYMS |
| P12643 | BMP2 |
| O15455 | TLR3 |
| P09211 | GSTP1 |
| P42224 | STAT1 |
| Q92887 | ABCC2 |
| P30556 | AGTR1 |
| P01909 | HLA-DQA1 |
| P16109 | SELP |
| P04035 | HMGCR |
| P06213 | INSR |
| P01344 | IGF2 |
| P10451 | SPP1 |
| P04141 | CSF2 |
| P11532 | DMD |
| P00742 | F10 |
| P13501 | CCL5 |
| P01127 | PDGFB |
| P11802 | CDK4 |
| O75874 | IDH1 |
| P00492 | HPRT1 |
| P08700 | IL3 |
| Q9Y2R2 | PTPN22 |
| P12318 | FCGR2A |
| P54098 | POLG |
| P61278 | SST |
| P51671 | CCL11 |
| P0C0L4 | C4A |
| P02545 | LMNA |
| Q9BZS1 | FOXP3 |
| P51580 | TPMT |
| Q16552 | IL17A |
| P16581 | SELE |
| P01024 | C3 |
| P51587 | BRCA2 |
| P19320 | VCAM1 |
| Q76LX8 | ADAMTS13 |
| P08253 | MMP2 |
| P28329 | CHAT |
| O75197 | LRP5 |
| P04179 | SOD2 |
| O15553 | MEFV |
| P03372 | ESR1 |
| P15502 | ELN |

| | |
|---|---|
| O60603 | TLR2 |
| P03956 | MMP1 |
| P48735 | IDH2 |
| P81172 | HAMP |
| P11226 | MBL2 |
| Q9UJS0 | SLC25A13 |
| P09104 | ENO2 |
| P15559 | NQO1 |
| Q06124 | PTPN11 |
| P04271 | S100B |
| P00738 | HP |
| Q8IZI9 | IFNL3 |
| P22303 | ACHE |
| Q03135 | CAV1 |
| P23219 | PTGS1 |
| P20823 | HNF1A |
| O15392 | BIRC5 |
| Q13315 | ATM |
| P00747 | PLG |
| O60260 | PRKN |
| Q13813 | SPTAN1 |
| P35498 | SCN1A |
| P0C0L5 | C4B |
| P42898 | MTHFR |
| P01236 | PRL |
| P30499 | HLA-C |
| P01019 | AGT |
| P05787 | KRT8 |
| Q9Y259 | CHKB |
| P16220 | CREB1 |
| Q16667 | CDKN3 |
| P35575 | G6PC |
| P51608 | MECP2 |
| P00395 | MT-CO1 |
| P10253 | GAA |
| P47989 | XDH |
| P15976 | GATA1 |
| P08670 | VIM |
| P08575 | PTPRC |
| O43520 | ATP8B1 |
| P09936 | UCHL1 |
| P01160 | NPPA |
| P05783 | KRT18 |
| P12004 | PCNA |
| P01189 | POMC |
| P08758 | ANXA5 |
| Q7Z6L0 | PRRT2 |
| P00749 | PLAU |
| P11912 | CD79A |
| P33527 | ABCC1 |
| Q9UNQ0 | ABCG2 |
| P20674 | COX5A |
| O75469 | NR1I2 |
| P25942 | CD40 |
| P34932 | HSPA4 |
| P00750 | PLAT |
| P02818 | BGLAP |
| P01111 | NRAS |

| | |
|---|---|
| P48357 | LEPR |
| Q13158 | FADD |
| Q15116 | PDCD1 |
| P29401 | TKT |
| P12931 | SRC |
| P11509 | CYP2A6 |
| O43181 | NDUFS4 |
| O00443 | PIK3C2A |
| P21583 | KITLG |
| P39905 | GDNF |
| P28223 | HTR2A |
| Q9BYX4 | IFIH1 |
| Q14653 | IRF3 |
| P14778 | IL1R1 |
| P06276 | BCHE |
| P02786 | TFRC |
| P49715 | CEBPA |
| P15056 | BRAF |
| P04629 | NTRK1 |
| P09429 | HMGB1 |
| P00390 | GSR |
| P09917 | ALOX5 |
| P21802 | FGFR2 |
| P48736 | PIK3CG |
| P09603 | CSF1 |
| P05187 | ALPP |
| P01011 | SERPINA3 |
| Q13568 | IRF5 |
| P60033 | CD81 |
| P01303 | NPY |
| O00220 | TNFRSF10A |
| P11473 | VDR |
| Q16620 | NTRK2 |
| P29466 | CASP1 |
| P49682 | CXCR3 |
| P17693 | HLA-G |
| P46531 | NOTCH1 |
| P21397 | MAOA |
| P14222 | PRF1 |
| Q9NST1 | PNPLA3 |
| P68871 | HBB |
| P08709 | F7 |
| O14936 | CASK |
| Q9BYW2 | SETD2 |
| P08603 | CFH |
| P00374 | DHFR |
| P01033 | TIMP1 |
| P35670 | ATP7B |
| P14174 | MIF |
| P05106 | ITGB3 |
| P04049 | RAF1 |
| P61769 | B2M |
| P04150 | NR3C1 |
| P00450 | CP |
| P06850 | CRH |
| P29475 | NOS1 |
| O60934 | NBN |
| P21757 | MSR1 |

| | |
|---|---|
| P16930 | FAH |
| Q09428 | ABCC8 |
| Q13427 | PPIG |
| P00813 | ADA |
| O14763 | TNFRSF10B |
| Q13224 | GRIN2B |
| P11362 | FGFR1 |
| P38398 | BRCA1 |
| Q00987 | MDM2 |
| P35372 | OPRM1 |
| P01574 | IFNB1 |
| P19544 | WT1 |
| Q9HD89 | RETN |
| P01266 | TG |
| P15941 | MUC1 |
| Q8NER1 | TRPV1 |
