# Supplementary material for: Systematic Elucidation of the Potential Mechanism of Erzhi Pill against Drug-Induced Liver Injury via Network Pharmacology Approach
Source: Evid Based Complement Alternat Med. 2020 Jan 6;2020:6219432. doi: 10.1155/2020/6219432 (PMC6970004; doi:10.1155/2020/6219432)
Supplement: https://www.uniprot.org/ Supplementary Materials — Table S1: putative targets for chemicals contained in EZP. Chemname was obtained from TCMSP. Entry, Entry name, and Protein name were obtained from PharmMapper. Entry was converted to Gene name by Uniprot. (https://www.uniprot.org/) Table S2: targets related to DILI. Detailed information of targets related to DILI, there were 267 targets from DrugBank and 357 targets from GeneCards with 42 targets duplicated. Table S3: common targets related to bioactive compounds. Detailed information of 89 common targets related to 23 bioactive compounds. Table S4: the results of GO and KEGG pathway enrichment. 223 BPs, 25 CCs, 65 MFs enriched have a P value less than 0.05, and 86 KEGG pathways were recognized as P < 0.05. [file 6219432.f1.zip › 6219432.f1/Supplementary Table S3.pdf]

Title: Uncovering the pharmacological mechanism of Erzhi pill on drug-induced liver injury based on network pharmacology

Table S3 Common targets related to bioactive compounds

Chemname	Entry	Gene names
(20S)-24-ene-3β,20-diol-3-acetate	P08581	MET
(20S)-24-ene-3β,20-diol-3-acetate	Q13133	NR1H3
(20S)-24-ene-3β,20-diol-3-acetate	Q04828	AKR1C1
(20S)-24-ene-3β,20-diol-3-acetate	P28482	MAPK1
(20S)-24-ene-3β,20-diol-3-acetate	P02766	TTR
(20S)-24-ene-3β,20-diol-3-acetate	P02768	ALB
(20S)-24-ene-3β,20-diol-3-acetate	P45983	MAPK8
(20S)-24-ene-3β,20-diol-3-acetate	P00734	F2
(20S)-24-ene-3β,20-diol-3-acetate	P35968	KDR
(20S)-24-ene-3β,20-diol-3-acetate	P06276	BCHE
(20S)-24-ene-3β,20-diol-3-acetate	P23141	CES1
(20S)-24-ene-3β,20-diol-3-acetate	P03372	ESR1
(20S)-24-ene-3β,20-diol-3-acetate	Q06520	SULT2A1
(20S)-24-ene-3β,20-diol-3-acetate	P24941	CDK2
(20S)-24-ene-3β,20-diol-3-acetate	P09211	GSTP1
(20S)-24-ene-3β,20-diol-3-acetate	P35398	RORA
(20S)-24-ene-3β,20-diol-3-acetate	P37231	PPARG
(20S)-24-ene-3β,20-diol-3-acetate	P08758	ANXA5
(20S)-24-ene-3β,20-diol-3-acetate	Q96RI1	NR1H4
(20S)-24-ene-3β,20-diol-3-acetate	P29474	NOS3
(20S)-24-ene-3β,20-diol-3-acetate	P18031	PTPN1
(20S)-24-ene-3β,20-diol-3-acetate	P00742	F10
(20S)-24-ene-3β,20-diol-3-acetate	P12643	BMP2
(20S)-24-ene-3β,20-diol-3-acetate	P12931	SRC
(20S)-24-ene-3β,20-diol-3-acetate	P00533	EGFR
(20S)-24-ene-3β,20-diol-3-acetate	P15056	BRAF
(20S)-24-ene-3β,20-diol-3-acetate	P42574	CASP3
(20S)-24-ene-3β,20-diol-3-acetate	P11142	HSPA8
(20S)-24-ene-3β,20-diol-3-acetate	P00390	GSR
(20S)-24-ene-3β,20-diol-3-acetate	P00374	DHFR
(20S)-24-ene-3β,20-diol-3-acetate	P04818	TYMS
(20S)-24-ene-3β,20-diol-3-acetate	Q16539	MAPK14
(20S)-24-ene-3β,20-diol-3-acetate	P11362	FGFR1
(20S)-24-ene-3β,20-diol-3-acetate	Q06124	PTPN11
(20S)-24-ene-3β,20-diol-3-acetate	P21802	FGFR2
(20S)-24-ene-3β,20-diol-3-acetate	P09874	PARP1
(20S)-24-ene-3β,20-diol-3-acetate	O15530	PDPK1
(20S)-24-ene-3β,20-diol-3-acetate	Q00987	MDM2
(20S)-24-ene-3β,20-diol-3-acetate	P15559	NQO1
3'-O-Methylorobol	O15530	PDPK1
3'-O-Methylorobol	P00374	DHFR
3'-O-Methylorobol	P00390	GSR
3'-O-Methylorobol	P00533	EGFR
3'-O-Methylorobol	P00734	F2
3'-O-Methylorobol	P00742	F10
3'-O-Methylorobol	P02768	ALB
3'-O-Methylorobol	P03372	ESR1
3'-O-Methylorobol	P03950	ANG
3'-O-Methylorobol	P04818	TYMS
3'-O-Methylorobol	P06276	BCHE
3'-O-Methylorobol	P06737	PYGL
3'-O-Methylorobol	P07900	HSP90AA1

3'-O-Methylorobol	P11142	HSPA8
3'-O-Methylorobol	P11362	FGFR1
3'-O-Methylorobol	P14174	MIF
3'-O-Methylorobol	P23141	CES1
3'-O-Methylorobol	P24941	CDK2
3'-O-Methylorobol	P29474	NOS3
3'-O-Methylorobol	P31749	AKT1
3'-O-Methylorobol	P35968	KDR
3'-O-Methylorobol	P45983	MAPK8
3'-O-Methylorobol	Q06520	SULT2A1
3'-O-Methylorobol	Q16539	MAPK14
Acacetin	O15530	PDPK1
Acacetin	P00374	DHFR
Acacetin	P00390	GSR
Acacetin	P00734	F2
Acacetin	P02766	TTR
Acacetin	P02768	ALB
Acacetin	P03372	ESR1
Acacetin	P03950	ANG
Acacetin	P06276	BCHE
Acacetin	P06737	PYGL
Acacetin	P07900	HSP90AA1
Acacetin	P11142	HSPA8
Acacetin	P14174	MIF
Acacetin	P15056	BRAF
Acacetin	P15559	NQO1
Acacetin	P23141	CES1
Acacetin	P24941	CDK2
Acacetin	P29474	NOS3
Acacetin	P35968	KDR
Acacetin	P45983	MAPK8
Acacetin	Q06520	SULT2A1
Acacetin	Q16539	MAPK14
Acacetin	Q16678	CYP1B1
beta-sitosterol	O15530	PDPK1
beta-sitosterol	P00533	EGFR
beta-sitosterol	P00734	F2
beta-sitosterol	P00742	F10
beta-sitosterol	P02753	RBP4
beta-sitosterol	P02766	TTR
beta-sitosterol	P02768	ALB
beta-sitosterol	P03372	ESR1
beta-sitosterol	P04035	HMGCR
beta-sitosterol	P04818	TYMS
beta-sitosterol	P06276	BCHE
beta-sitosterol	P08758	ANXA5
beta-sitosterol	P12643	BMP2
beta-sitosterol	P12931	SRC
beta-sitosterol	P15056	BRAF
beta-sitosterol	P15559	NQO1
beta-sitosterol	P23141	CES1
beta-sitosterol	P24941	CDK2
beta-sitosterol	P28482	MAPK1
beta-sitosterol	P29474	NOS3
beta-sitosterol	P35398	RORA
beta-sitosterol	P35968	KDR
beta-sitosterol	P37231	PPARG
beta-sitosterol	P45983	MAPK8

beta-sitosterol	P49638	TTPA
beta-sitosterol	P55055	NR1H2
beta-sitosterol	Q04828	AKR1C1
beta-sitosterol	Q06124	PTPN11
beta-sitosterol	Q06520	SULT2A1
beta-sitosterol	Q13133	NR1H3
beta-sitosterol	Q16539	MAPK14
beta-sitosterol	Q96RI1	NR1H4
Butin	O15530	PDPK1
Butin	P00374	DHFR
Butin	P00390	GSR
Butin	P00734	F2
Butin	P02766	TTR
Butin	P02768	ALB
Butin	P03372	ESR1
Butin	P03950	ANG
Butin	P06276	BCHE
Butin	P06737	PYGL
Butin	P07900	HSP90AA1
Butin	P11142	HSPA8
Butin	P14174	MIF
Butin	P15056	BRAF
Butin	P15559	NQO1
Butin	P23141	CES1
Butin	P24941	CDK2
Butin	P29474	NOS3
Butin	P35968	KDR
Butin	P45983	MAPK8
Butin	Q06520	SULT2A1
Butin	Q16539	MAPK14
Butin	Q16678	CYP1B1
Demethylwedelolactone	O15530	PDPK1
Demethylwedelolactone	P00374	DHFR
Demethylwedelolactone	P00390	GSR
Demethylwedelolactone	P00533	EGFR
Demethylwedelolactone	P00734	F2
Demethylwedelolactone	P02766	TTR
Demethylwedelolactone	P02768	ALB
Demethylwedelolactone	P03372	ESR1
Demethylwedelolactone	P03950	ANG
Demethylwedelolactone	P04179	SOD2
Demethylwedelolactone	P04818	TYMS
Demethylwedelolactone	P06276	BCHE
Demethylwedelolactone	P07900	HSP90AA1
Demethylwedelolactone	P11142	HSPA8
Demethylwedelolactone	P11362	FGFR1
Demethylwedelolactone	P12643	BMP2
Demethylwedelolactone	P12931	SRC
Demethylwedelolactone	P14174	MIF
Demethylwedelolactone	P23141	CES1
Demethylwedelolactone	P24941	CDK2
Demethylwedelolactone	P29474	NOS3
Demethylwedelolactone	P35968	KDR
Demethylwedelolactone	P45983	MAPK8
Demethylwedelolactone	P48736	PIK3CG
Demethylwedelolactone	Q06520	SULT2A1
Demethylwedelolactone	Q16539	MAPK14
Eriodictyol	O15530	PDPK1

Eriodictyol	P00374	DHFR
Eriodictyol	P00390	GSR
Eriodictyol	P00533	EGFR
Eriodictyol	P00734	F2
Eriodictyol	P00742	F10
Eriodictyol	P02766	TTR
Eriodictyol	P02768	ALB
Eriodictyol	P03372	ESR1
Eriodictyol	P03950	ANG
Eriodictyol	P04818	TYMS
Eriodictyol	P06276	BCHE
Eriodictyol	P06737	PYGL
Eriodictyol	P07900	HSP90AA1
Eriodictyol	P08758	ANXA5
Eriodictyol	P09211	GSTP1
Eriodictyol	P11142	HSPA8
Eriodictyol	P11362	FGFR1
Eriodictyol	P12931	SRC
Eriodictyol	P14174	MIF
Eriodictyol	P15559	NQO1
Eriodictyol	P23141	CES1
Eriodictyol	P24941	CDK2
Eriodictyol	P29474	NOS3
Eriodictyol	P31749	AKT1
Eriodictyol	P35968	KDR
Eriodictyol	P45983	MAPK8
Eriodictyol	P48736	PIK3CG
Eriodictyol	Q06520	SULT2A1
Eriodictyol	Q16539	MAPK14
Eriodictyol	Q16678	CYP1B1
Kaempferol	O15530	PDPK1
Kaempferol	P00374	DHFR
Kaempferol	P00390	GSR
Kaempferol	P00533	EGFR
Kaempferol	P00734	F2
Kaempferol	P02766	TTR
Kaempferol	P03372	ESR1
Kaempferol	P03950	ANG
Kaempferol	P06276	BCHE
Kaempferol	P06737	PYGL
Kaempferol	P07900	HSP90AA1
Kaempferol	P08183	ABCB1
Kaempferol	P08253	MMP2
Kaempferol	P09917	ALOX5
Kaempferol	P11142	HSPA8
Kaempferol	P12931	SRC
Kaempferol	P14780	MMP9
Kaempferol	P15559	NQO1
Kaempferol	P21397	MAOA
Kaempferol	P22303	ACHE
Kaempferol	P24941	CDK2
Kaempferol	P33527	ABCC1
Kaempferol	P36888	FLT3
Kaempferol	P45983	MAPK8
Kaempferol	P47989	XDH
Kaempferol	P55055	NR1H2
Kaempferol	Q04760	GLO1
Kaempferol	Q16678	CYP1B1

Kaempferol	Q9UNQ0	ABCG2
Linarin	O15530	PDPK1
Linarin	P00374	DHFR
Linarin	P00390	GSR
Linarin	P00533	EGFR
Linarin	P00734	F2
Linarin	P00742	F10
Linarin	P00749	PLAU
Linarin	P00797	REN
Linarin	P01009	SERPINA1
Linarin	P02766	TTR
Linarin	P02768	ALB
Linarin	P03372	ESR1
Linarin	P03950	ANG
Linarin	P04179	SOD2
Linarin	P04818	TYMS
Linarin	P06276	BCHE
Linarin	P06737	PYGL
Linarin	P07900	HSP90AA1
Linarin	P08246	ELANE
Linarin	P08581	MET
Linarin	P08758	ANXA5
Linarin	P09211	GSTP1
Linarin	P11142	HSPA8
Linarin	P11362	FGFR1
Linarin	P12931	SRC
Linarin	P14174	MIF
Linarin	P15056	BRAF
Linarin	P15153	RAC2
Linarin	P15559	NQO1
Linarin	P18031	PTPN1
Linarin	P23141	CES1
Linarin	P24941	CDK2
Linarin	P28482	MAPK1
Linarin	P29474	NOS3
Linarin	P30043	BLVRB
Linarin	P31749	AKT1
Linarin	P35228	NOS2
Linarin	P35968	KDR
Linarin	P37231	PPARG
Linarin	P42574	CASP3
Linarin	P45983	MAPK8
Linarin	P55055	NR1H2
Linarin	Q04760	GLO1
Linarin	Q04828	AKR1C1
Linarin	Q06520	SULT2A1
Linarin	Q07912	TNK2
Linarin	Q16539	MAPK14
Lucidumoside D	O15530	PDPK1
Lucidumoside D	P00374	DHFR
Lucidumoside D	P00390	GSR
Lucidumoside D	P00533	EGFR
Lucidumoside D	P00734	F2
Lucidumoside D	P00742	F10
Lucidumoside D	P00797	REN
Lucidumoside D	P01009	SERPINA1
Lucidumoside D	P02766	TTR
Lucidumoside D	P02768	ALB

Lucidumoside D	P03372	ESR1
Lucidumoside D	P03950	ANG
Lucidumoside D	P04035	HMGCR
Lucidumoside D	P04818	TYMS
Lucidumoside D	P05019	IGF1
Lucidumoside D	P06276	BCHE
Lucidumoside D	P06737	PYGL
Lucidumoside D	P07900	HSP90AA1
Lucidumoside D	P08581	MET
Lucidumoside D	P08758	ANXA5
Lucidumoside D	P09211	GSTP1
Lucidumoside D	P09874	PARP1
Lucidumoside D	P11142	HSPA8
Lucidumoside D	P11362	FGFR1
Lucidumoside D	P11712	CYP2C9
Lucidumoside D	P12931	SRC
Lucidumoside D	P14174	MIF
Lucidumoside D	P15056	BRAF
Lucidumoside D	P15153	RAC2
Lucidumoside D	P15559	NQO1
Lucidumoside D	P18031	PTPN1
Lucidumoside D	P23141	CES1
Lucidumoside D	P24941	CDK2
Lucidumoside D	P28482	MAPK1
Lucidumoside D	P29474	NOS3
Lucidumoside D	P31749	AKT1
Lucidumoside D	P35398	RORA
Lucidumoside D	P35968	KDR
Lucidumoside D	P37231	PPARG
Lucidumoside D	P39900	MMP12
Lucidumoside D	P45983	MAPK8
Lucidumoside D	P48736	PIK3CG
Lucidumoside D	P55055	NR1H2
Lucidumoside D	Q06520	SULT2A1
Lucidumoside D	Q07912	TNK2
Lucidumoside D	Q16539	MAPK14
Lucidumoside D_qt	O15530	PDPK1
Lucidumoside D_qt	P00374	DHFR
Lucidumoside D_qt	P00390	GSR
Lucidumoside D_qt	P00533	EGFR
Lucidumoside D_qt	P00734	F2
Lucidumoside D_qt	P00742	F10
Lucidumoside D_qt	P02766	TTR
Lucidumoside D_qt	P02768	ALB
Lucidumoside D_qt	P03372	ESR1
Lucidumoside D_qt	P03950	ANG
Lucidumoside D_qt	P04818	TYMS
Lucidumoside D_qt	P06276	BCHE
Lucidumoside D_qt	P06737	PYGL
Lucidumoside D_qt	P07900	HSP90AA1
Lucidumoside D_qt	P08758	ANXA5
Lucidumoside D_qt	P09211	GSTP1
Lucidumoside D_qt	P09874	PARP1
Lucidumoside D_qt	P11142	HSPA8
Lucidumoside D_qt	P11712	CYP2C9
Lucidumoside D_qt	P12931	SRC
Lucidumoside D_qt	P14174	MIF
Lucidumoside D_qt	P15056	BRAF

Lucidumoside D_qt	P18031	PTPN1
Lucidumoside D_qt	P23141	CES1
Lucidumoside D_qt	P24941	CDK2
Lucidumoside D_qt	P28482	MAPK1
Lucidumoside D_qt	P29474	NOS3
Lucidumoside D_qt	P31749	AKT1
Lucidumoside D_qt	P35398	RORA
Lucidumoside D_qt	P35968	KDR
Lucidumoside D_qt	P37231	PPARG
Lucidumoside D_qt	P39900	MMP12
Lucidumoside D_qt	P45983	MAPK8
Lucidumoside D_qt	P55055	NR1H2
Lucidumoside D_qt	Q06520	SULT2A1
Lucidumoside D_qt	Q16539	MAPK14
Lucidusculine	O15530	PDPK1
Lucidusculine	O75469	NR1I2
Lucidusculine	P00374	DHFR
Lucidusculine	P00390	GSR
Lucidusculine	P00533	EGFR
Lucidusculine	P00734	F2
Lucidusculine	P00742	F10
Lucidusculine	P00747	PLG
Lucidusculine	P01009	SERPINA1
Lucidusculine	P02766	TTR
Lucidusculine	P02768	ALB
Lucidusculine	P03372	ESR1
Lucidusculine	P03950	ANG
Lucidusculine	P04179	SOD2
Lucidusculine	P04818	TYMS
Lucidusculine	P06276	BCHE
Lucidusculine	P07900	HSP90AA1
Lucidusculine	P08246	ELANE
Lucidusculine	P08581	MET
Lucidusculine	P08709	F7
Lucidusculine	P08758	ANXA5
Lucidusculine	P09211	GSTP1
Lucidusculine	P11712	CYP2C9
Lucidusculine	P12643	BMP2
Lucidusculine	P12931	SRC
Lucidusculine	P15559	NQO1
Lucidusculine	P18031	PTPN1
Lucidusculine	P21802	FGFR2
Lucidusculine	P24941	CDK2
Lucidusculine	P28482	MAPK1
Lucidusculine	P29474	NOS3
Lucidusculine	P30043	BLVRB
Lucidusculine	P35398	RORA
Lucidusculine	P35968	KDR
Lucidusculine	P37231	PPARG
Lucidusculine	P45983	MAPK8
Lucidusculine	P49638	TTPA
Lucidusculine	P55055	NR1H2
Lucidusculine	Q00987	MDM2
Lucidusculine	Q04828	AKR1C1
Lucidusculine	Q06124	PTPN11
Lucidusculine	Q06520	SULT2A1
Lucidusculine	Q13133	NR1H3
Lucidusculine	Q16539	MAPK14

Lucidusculine	Q96RI1	NR1H4
Luteolin	O15530	PDPK1
Luteolin	P00374	DHFR
Luteolin	P00390	GSR
Luteolin	P00533	EGFR
Luteolin	P00734	F2
Luteolin	P00742	F10
Luteolin	P02766	TTR
Luteolin	P02768	ALB
Luteolin	P03372	ESR1
Luteolin	P03950	ANG
Luteolin	P04818	TYMS
Luteolin	P05067	APP
Luteolin	P05089	ARG1
Luteolin	P06276	BCHE
Luteolin	P06737	PYGL
Luteolin	P07900	HSP90AA1
Luteolin	P08253	MMP2
Luteolin	P09211	GSTP1
Luteolin	P09874	PARP1
Luteolin	P09917	ALOX5
Luteolin	P11142	HSPA8
Luteolin	P12931	SRC
Luteolin	P14174	MIF
Luteolin	P14780	MMP9
Luteolin	P15559	NQO1
Luteolin	P21397	MAOA
Luteolin	P22303	ACHE
Luteolin	P23141	CES1
Luteolin	P24941	CDK2
Luteolin	P28907	CD38
Luteolin	P29474	NOS3
Luteolin	P31749	AKT1
Luteolin	P33527	ABCC1
Luteolin	P35968	KDR
Luteolin	P36888	FLT3
Luteolin	P39900	MMP12
Luteolin	P45983	MAPK8
Luteolin	P47989	XDH
Luteolin	P48736	PIK3CG
Luteolin	Q04760	GLO1
Luteolin	Q06520	SULT2A1
Luteolin	Q16539	MAPK14
Luteolin	Q16678	CYP1B1
Luteolin	Q9UNQ0	ABCG2
Oleanolic acid	O15530	PDPK1
Oleanolic acid	P00390	GSR
Oleanolic acid	P00533	EGFR
Oleanolic acid	P00734	F2
Oleanolic acid	P00797	REN
Oleanolic acid	P02766	TTR
Oleanolic acid	P02768	ALB
Oleanolic acid	P03372	ESR1
Oleanolic acid	P06276	BCHE
Oleanolic acid	P07900	HSP90AA1
Oleanolic acid	P08581	MET
Oleanolic acid	P08758	ANXA5
Oleanolic acid	P09211	GSTP1

Oleanolic acid	P09874	PARP1
Oleanolic acid	P12643	BMP2
Oleanolic acid	P12931	SRC
Oleanolic acid	P15056	BRAF
Oleanolic acid	P18031	PTPN1
Oleanolic acid	P23141	CES1
Oleanolic acid	P24941	CDK2
Oleanolic acid	P28482	MAPK1
Oleanolic acid	P29474	NOS3
Oleanolic acid	P35398	RORA
Oleanolic acid	P35968	KDR
Oleanolic acid	P37231	PPARG
Oleanolic acid	P45983	MAPK8
Oleanolic acid	P55055	NR1H2
Oleanolic acid	Q04828	AKR1C1
Oleanolic acid	Q06124	PTPN11
Oleanolic acid	Q06520	SULT2A1
Oleanolic acid	Q16539	MAPK14
Oleanolic acid	Q96RI1	NR1H4
Olitoriside	O15530	PDPK1
Olitoriside	P00374	DHFR
Olitoriside	P00390	GSR
Olitoriside	P00533	EGFR
Olitoriside	P00734	F2
Olitoriside	P00742	F10
Olitoriside	P00749	PLAU
Olitoriside	P00797	REN
Olitoriside	P02766	TTR
Olitoriside	P02768	ALB
Olitoriside	P03372	ESR1
Olitoriside	P03950	ANG
Olitoriside	P04818	TYMS
Olitoriside	P05019	IGF1
Olitoriside	P06276	BCHE
Olitoriside	P06737	PYGL
Olitoriside	P07900	HSP90AA1
Olitoriside	P08246	ELANE
Olitoriside	P08758	ANXA5
Olitoriside	P09211	GSTP1
Olitoriside	P11142	HSPA8
Olitoriside	P11362	FGFR1
Olitoriside	P12643	BMP2
Olitoriside	P12931	SRC
Olitoriside	P14174	MIF
Olitoriside	P15056	BRAF
Olitoriside	P15153	RAC2
Olitoriside	P15559	NQO1
Olitoriside	P23141	CES1
Olitoriside	P24941	CDK2
Olitoriside	P28482	MAPK1
Olitoriside	P29474	NOS3
Olitoriside	P31749	AKT1
Olitoriside	P35398	RORA
Olitoriside	P35968	KDR
Olitoriside	P37231	PPARG
Olitoriside	P39900	MMP12
Olitoriside	P42574	CASP3
Olitoriside	P45983	MAPK8

Olitoriside	P49638	TTPA
Olitoriside	P55055	NR1H2
Olitoriside	Q06520	SULT2A1
Olitoriside	Q07912	TNK2
Olitoriside	Q13133	NR1H3
Olitoriside	Q16539	MAPK14
Olitoriside_qt	O15530	PDPK1
Olitoriside_qt	P00374	DHFR
Olitoriside_qt	P00533	EGFR
Olitoriside_qt	P02766	TTR
Olitoriside_qt	P03372	ESR1
Olitoriside_qt	P04179	SOD2
Olitoriside_qt	P04818	TYMS
Olitoriside_qt	P08758	ANXA5
Olitoriside_qt	P12643	BMP2
Olitoriside_qt	P12931	SRC
Olitoriside_qt	P29474	NOS3
Olitoriside_qt	P37231	PPARG
Olitoriside_qt	P55055	NR1H2
Olitoriside_qt	Q06520	SULT2A1
Olitoriside_qt	Q16539	MAPK14
Pratensein	O15530	PDPK1
Pratensein	P00374	DHFR
Pratensein	P00390	GSR
Pratensein	P00533	EGFR
Pratensein	P00734	F2
Pratensein	P00742	F10
Pratensein	P02766	TTR
Pratensein	P02768	ALB
Pratensein	P03372	ESR1
Pratensein	P03950	ANG
Pratensein	P04818	TYMS
Pratensein	P06276	BCHE
Pratensein	P06737	PYGL
Pratensein	P07900	HSP90AA1
Pratensein	P11142	HSPA8
Pratensein	P11362	FGFR1
Pratensein	P12931	SRC
Pratensein	P14174	MIF
Pratensein	P15559	NQO1
Pratensein	P23141	CES1
Pratensein	P24941	CDK2
Pratensein	P29474	NOS3
Pratensein	P31749	AKT1
Pratensein	P35968	KDR
Pratensein	P45983	MAPK8
Pratensein	Q06520	SULT2A1
Pratensein	Q07912	TNK2
Pratensein	Q16539	MAPK14
Quercetin	O15530	PDPK1
Quercetin	O60285	NUAK1
Quercetin	P00374	DHFR
Quercetin	P00390	GSR
Quercetin	P00533	EGFR
Quercetin	P00734	F2
Quercetin	P00797	REN
Quercetin	P02766	TTR
Quercetin	P03372	ESR1

Quercetin	P03950	ANG
Quercetin	P05164	MPO
Quercetin	P06213	INSR
Quercetin	P06276	BCHE
Quercetin	P06737	PYGL
Quercetin	P08183	ABCB1
Quercetin	P08253	MMP2
Quercetin	P08581	MET
Quercetin	P09211	GSTP1
Quercetin	P09917	ALOX5
Quercetin	P10636	MAPT
Quercetin	P11142	HSPA8
Quercetin	P11362	FGFR1
Quercetin	P12931	SRC
Quercetin	P14174	MIF
Quercetin	P14780	MMP9
Quercetin	P15559	NQO1
Quercetin	P21397	MAOA
Quercetin	P22303	ACHE
Quercetin	P24941	CDK2
Quercetin	P27986	PIK3R1
Quercetin	P31749	AKT1
Quercetin	P33527	ABCC1
Quercetin	P35968	KDR
Quercetin	P36888	FLT3
Quercetin	P45983	MAPK8
Quercetin	P47989	XDH
Quercetin	P48736	PIK3CG
Quercetin	P55055	NR1H2
Quercetin	Q04760	GLO1
Quercetin	Q04828	AKR1C1
Quercetin	Q15746	MYLK
Quercetin	Q16539	MAPK14
Quercetin	Q16678	CYP1B1
Quercetin	Q96GD4	AURKB
Quercetin	Q9UNQ0	ABCG2
Salidroside	O15530	PDPK1
Salidroside	P00374	DHFR
Salidroside	P00390	GSR
Salidroside	P00533	EGFR
Salidroside	P00734	F2
Salidroside	P00742	F10
Salidroside	P00797	REN
Salidroside	P02766	TTR
Salidroside	P03372	ESR1
Salidroside	P03950	ANG
Salidroside	P04035	HMGCR
Salidroside	P04179	SOD2
Salidroside	P04818	TYMS
Salidroside	P06276	BCHE
Salidroside	P06737	PYGL
Salidroside	P07900	HSP90AA1
Salidroside	P08758	ANXA5
Salidroside	P09211	GSTP1
Salidroside	P11142	HSPA8
Salidroside	P11362	FGFR1
Salidroside	P12931	SRC
Salidroside	P14174	MIF

Salidroside	P18031	PTPN1
Salidroside	P24941	CDK2
Salidroside	P28482	MAPK1
Salidroside	P29474	NOS3
Salidroside	P31749	AKT1
Salidroside	P35968	KDR
Salidroside	P37231	PPARG
Salidroside	P42574	CASP3
Salidroside	P45983	MAPK8
Salidroside	P55055	NR1H2
Salidroside	Q06520	SULT2A1
Salidroside	Q07912	TNK2
Salidroside	Q16539	MAPK14
Specnuezhenide	O15530	PDPK1
Specnuezhenide	P00374	DHFR
Specnuezhenide	P00390	GSR
Specnuezhenide	P00533	EGFR
Specnuezhenide	P00734	F2
Specnuezhenide	P00742	F10
Specnuezhenide	P00749	PLAU
Specnuezhenide	P02766	TTR
Specnuezhenide	P03372	ESR1
Specnuezhenide	P03950	ANG
Specnuezhenide	P04035	HMGCR
Specnuezhenide	P04179	SOD2
Specnuezhenide	P04818	TYMS
Specnuezhenide	P06276	BCHE
Specnuezhenide	P06737	PYGL
Specnuezhenide	P07900	HSP90AA1
Specnuezhenide	P08758	ANXA5
Specnuezhenide	P09211	GSTP1
Specnuezhenide	P11142	HSPA8
Specnuezhenide	P11362	FGFR1
Specnuezhenide	P12931	SRC
Specnuezhenide	P14174	MIF
Specnuezhenide	P18031	PTPN1
Specnuezhenide	P23141	CES1
Specnuezhenide	P24941	CDK2
Specnuezhenide	P29474	NOS3
Specnuezhenide	P31749	AKT1
Specnuezhenide	P35968	KDR
Specnuezhenide	P37231	PPARG
Specnuezhenide	P39900	MMP12
Specnuezhenide	P42574	CASP3
Specnuezhenide	P45983	MAPK8
Specnuezhenide	P55055	NR1H2
Specnuezhenide	P60568	IL2
Specnuezhenide	Q06520	SULT2A1
Specnuezhenide	Q07912	TNK2
Specnuezhenide	Q16539	MAPK14
Syringaresinol diglucoside_qt	O15530	PDPK1
Syringaresinol diglucoside_qt	O75469	NR1I2
Syringaresinol diglucoside_qt	P00374	DHFR
Syringaresinol diglucoside_qt	P00390	GSR
Syringaresinol diglucoside_qt	P00533	EGFR
Syringaresinol diglucoside_qt	P00734	F2
Syringaresinol diglucoside_qt	P00742	F10
Syringaresinol diglucoside_qt	P00797	REN

Syringaresinol diglucoside_qt	P02753	RBP4
Syringaresinol diglucoside_qt	P02766	TTR
Syringaresinol diglucoside_qt	P02768	ALB
Syringaresinol diglucoside_qt	P03372	ESR1
Syringaresinol diglucoside_qt	P03950	ANG
Syringaresinol diglucoside_qt	P04179	SOD2
Syringaresinol diglucoside_qt	P04818	TYMS
Syringaresinol diglucoside_qt	P05019	IGF1
Syringaresinol diglucoside_qt	P06276	BCHE
Syringaresinol diglucoside_qt	P06737	PYGL
Syringaresinol diglucoside_qt	P07900	HSP90AA1
Syringaresinol diglucoside_qt	P08246	ELANE
Syringaresinol diglucoside_qt	P08581	MET
Syringaresinol diglucoside_qt	P08709	F7
Syringaresinol diglucoside_qt	P09211	GSTP1
Syringaresinol diglucoside_qt	P09874	PARP1
Syringaresinol diglucoside_qt	P11142	HSPA8
Syringaresinol diglucoside_qt	P11362	FGFR1
Syringaresinol diglucoside_qt	P11712	CYP2C9
Syringaresinol diglucoside_qt	P12931	SRC
Syringaresinol diglucoside_qt	P14174	MIF
Syringaresinol diglucoside_qt	P15056	BRAF
Syringaresinol diglucoside_qt	P15559	NQO1
Syringaresinol diglucoside_qt	P16109	SELP
Syringaresinol diglucoside_qt	P18031	PTPN1
Syringaresinol diglucoside_qt	P21802	FGFR2
Syringaresinol diglucoside_qt	P23141	CES1
Syringaresinol diglucoside_qt	P24941	CDK2
Syringaresinol diglucoside_qt	P28482	MAPK1
Syringaresinol diglucoside_qt	P29474	NOS3
Syringaresinol diglucoside_qt	P30043	BLVRB
Syringaresinol diglucoside_qt	P35398	RORA
Syringaresinol diglucoside_qt	P35968	KDR
Syringaresinol diglucoside_qt	P37231	PPARG
Syringaresinol diglucoside_qt	P39900	MMP12
Syringaresinol diglucoside_qt	P42574	CASP3
Syringaresinol diglucoside_qt	P45983	MAPK8
Syringaresinol diglucoside_qt	P48736	PIK3CG
Syringaresinol diglucoside_qt	P55055	NR1H2
Syringaresinol diglucoside_qt	Q00987	MDM2
Syringaresinol diglucoside_qt	Q04828	AKR1C1
Syringaresinol diglucoside_qt	Q06520	SULT2A1
Syringaresinol diglucoside_qt	Q07869	PPARA
Syringaresinol diglucoside_qt	Q13133	NR1H3
Syringaresinol diglucoside_qt	Q16539	MAPK14
Taxifolin	O15530	PDPK1
Taxifolin	P00374	DHFR
Taxifolin	P00390	GSR
Taxifolin	P00533	EGFR
Taxifolin	P00734	F2
Taxifolin	P00742	F10
Taxifolin	P00749	PLAU
Taxifolin	P02766	TTR
Taxifolin	P02768	ALB
Taxifolin	P03372	ESR1
Taxifolin	P03950	ANG
Taxifolin	P06276	BCHE
Taxifolin	P06737	PYGL

Taxifolin	P07900	HSP90AA1
Taxifolin	P08758	ANXA5
Taxifolin	P09211	GSTP1
Taxifolin	P11142	HSPA8
Taxifolin	P12931	SRC
Taxifolin	P15559	NQO1
Taxifolin	P23141	CES1
Taxifolin	P24941	CDK2
Taxifolin	P29474	NOS3
Taxifolin	P35968	KDR
Taxifolin	P39900	MMP12
Taxifolin	P45983	MAPK8
Taxifolin	P48736	PIK3CG
Taxifolin	P55055	NR1H2
Taxifolin	Q06520	SULT2A1
Taxifolin	Q16539	MAPK14
Wedelolactone	O15530	PDPK1
Wedelolactone	P00374	DHFR
Wedelolactone	P00390	GSR
Wedelolactone	P00533	EGFR
Wedelolactone	P00734	F2
Wedelolactone	P00742	F10
Wedelolactone	P02766	TTR
Wedelolactone	P02768	ALB
Wedelolactone	P03372	ESR1
Wedelolactone	P03950	ANG
Wedelolactone	P04179	SOD2
Wedelolactone	P04818	TYMS
Wedelolactone	P06276	BCHE
Wedelolactone	P07900	HSP90AA1
Wedelolactone	P09211	GSTP1
Wedelolactone	P09917	ALOX5
Wedelolactone	P11142	HSPA8
Wedelolactone	P11362	FGFR1
Wedelolactone	P12643	BMP2
Wedelolactone	P12931	SRC
Wedelolactone	P14174	MIF
Wedelolactone	P23141	CES1
Wedelolactone	P24941	CDK2
Wedelolactone	P29474	NOS3
Wedelolactone	P35968	KDR
Wedelolactone	P45983	MAPK8
Wedelolactone	P48736	PIK3CG
Wedelolactone	P55055	NR1H2
Wedelolactone	Q06520	SULT2A1
Wedelolactone	Q16539	MAPK14
