# Supplementary material for: Systematic Elucidation of the Potential Mechanism of Erzhi Pill against Drug-Induced Liver Injury via Network Pharmacology Approach
Source: Evid Based Complement Alternat Med. 2020 Jan 6;2020:6219432. doi: 10.1155/2020/6219432 (PMC6970004; doi:10.1155/2020/6219432)
Supplement: https://www.uniprot.org/ Supplementary Materials — Table S1: putative targets for chemicals contained in EZP. Chemname was obtained from TCMSP. Entry, Entry name, and Protein name were obtained from PharmMapper. Entry was converted to Gene name by Uniprot. (https://www.uniprot.org/) Table S2: targets related to DILI. Detailed information of targets related to DILI, there were 267 targets from DrugBank and 357 targets from GeneCards with 42 targets duplicated. Table S3: common targets related to bioactive compounds. Detailed information of 89 common targets related to 23 bioactive compounds. Table S4: the results of GO and KEGG pathway enrichment. 223 BPs, 25 CCs, 65 MFs enriched have a P value less than 0.05, and 86 KEGG pathways were recognized as P < 0.05. [file 6219432.f1.zip › 6219432.f1/Supplementary Table S4.pdf]

Title: Uncovering the pharmacological mechanism of Erzhi pill on drug-induced liver injury based on network pharmacology

Table S4 The results of GO and KEGG pathway enrichment

**Category**	**Term**	**Count**	**%**	**PValue**	**Genes**
KEGG_PATHWAY	hsa05200:Pathways in cancer	23	25.84	1E-10	P05019, P36888, P11362, P08581, P48736, P14780, P35228, P24941, P07900, P27986, Q00987, P15153, P09211, P08253, P28482, P21802, P42574, P37231, P00533, P12643, P31749, P15056, P45983
KEGG_PATHWAY	hsa05205:Proteoglycans in cancer	20	22.47	2.4E-13	P05019, P12931, P11362, P08581, P48736, P14780, P03372, P27986, P35968, O15530, Q00987, P08253, Q06124, P28482, P42574, P00749, Q16539, P00533, P31749, P15056
KEGG_PATHWAY	hsa04151:PI3K-Akt signaling pathway	17	19.10	7.8E-07	P05019, P11362, P08581, P48736, P24941, P60568, P07900, P27986, P35968, O15530, Q00987, P29474, P21802, P28482, P06213, P00533, P31749
KEGG_PATHWAY	hsa04015:Rap1 signaling pathway	15	16.85	5.1E-08	P05019, P12931, P11362, P08581, P48736, P27986, P35968, P15153, P21802, P28482, P06213, Q16539, P00533, P31749, P15056
KEGG_PATHWAY	hsa04068:FoxO signaling pathway	14	15.73	1.7E-09	P05019, P48736, P24941, P27986, O15530, Q00987, P04179, P28482, P06213, Q16539, P00533, P31749, P45983, P15056
KEGG_PATHWAY	hsa04510:Focal adhesion	14	15.73	3.1E-07	P05019, P12931, P08581, P48736, P27986, P35968, Q15746, O15530, P15153, P28482, P00533, P31749, P45983, P15056
KEGG_PATHWAY	hsa04014:Ras signaling pathway	14	15.73	9E-07	P05019, P27986, P35968, P15153, P11362, Q06124, P08581, P28482, P21802, P48736, P06213, P00533, P31749, P45983
KEGG_PATHWAY	hsa05215:Prostate cancer	13	14.61	1.4E-10	P05019, P11362, P48736, P24941, P27986, P07900, O15530, Q00987, P21802, P28482, P00533, P31749, P15056
KEGG_PATHWAY	hsa04931:Insulin resistance	13	14.61	1.6E-09	Q13133, P48736, P06737, P18031, P27986, O15530, P29474, Q06124, P06213, Q07869, P31749, P55055, P45983
KEGG_PATHWAY	hsa04915:Estrogen signaling pathway	12	13.48	8.2E-09	P07900, P27986, P11142, P03372, P12931, P29474, P08253, P28482, P48736, P14780, P00533, P31749
KEGG_PATHWAY	hsa04010:MAPK signaling pathway	12	13.48	9.2E-05	P10636, P11142, P15153, P11362, P28482, P21802, P42574, Q16539, P00533, P31749, P15056, P45983

KEGG_PATHWAY	hsa05160:Hepatitis C	11	12.36	1.7E-06	P27986, Q13133, O15530, P28482, P48736, Q16539, Q07869, P00533, P31749, P15056, P45983
KEGG_PATHWAY	hsa04810:Regulation of actin cytoskeleton	11	12.36	9.3E-05	P27986, Q15746, P12931, P15153, P11362, P28482, P21802, P48736, P00533, P00734, P15056
KEGG_PATHWAY	hsa05218:Melanoma	10	11.24	6.5E-08	P27986, P05019, Q00987, P11362, P08581, P28482, P48736, P00533, P31749, P15056
KEGG_PATHWAY	hsa04914:Progesterone-mediated oocyte maturation	10	11.24	3.9E-07	P07900, P27986, P05019, P28482, P48736, Q16539, P24941, P31749, P15056, P45983
KEGG_PATHWAY	hsa04071:Sphingolipid signaling pathway	10	11.24	5.9E-06	P27986, O15530, P15153, P33527, P29474, P28482, P48736, Q16539, P31749, P45983
KEGG_PATHWAY	hsa04910:Insulin signaling pathway	10	11.24	1.8E-05	P27986, O15530, P28482, P48736, P06213, P06737, P18031, P31749, P15056, P45983
KEGG_PATHWAY	hsa04370:VEGF signaling pathway	9	10.11	2.8E-07	P27986, P35968, P12931, P15153, P29474, P28482, P48736, Q16539, P31749
KEGG_PATHWAY	hsa05230:Central carbon metabolism in cancer	9	10.11	4.1E-07	P27986, P36888, P11362, P08581, P28482, P21802, P48736, P00533, P31749
KEGG_PATHWAY	hsa04066:HIF-1 signaling pathway	9	10.11	9.3E-06	P27986, P05019, P29474, P28482, P48736, P06213, P35228, P00533, P31749
KEGG_PATHWAY	hsa05145:Toxoplasmosis	9	10.11	2.5E-05	P11142, P09917, O15530, P28482, P42574, Q16539, P35228, P31749, P45983
KEGG_PATHWAY	hsa04722:Neurotrophin signaling pathway	9	10.11	4.8E-05	P27986, O15530, Q06124, P28482, P48736, Q16539, P31749, P15056, P45983
KEGG_PATHWAY	hsa04550:Signaling pathways regulating pluripotency of stem cells	9	10.11	0.00014	P27986, P05019, P11362, P28482, P21802, P48736, Q16539, P12643, P31749
KEGG_PATHWAY	hsa05161:Hepatitis B	9	10.11	0.00018	P27986, P12931, P28482, P48736, P42574, P14780, P24941, P31749, P45983
KEGG_PATHWAY	hsa05202:Transcriptional misregulation in cancer	9	10.11	0.00047	P05164, P08246, P05019, P36888, Q00987, P08581, P14780, P00749, P37231
KEGG_PATHWAY	hsa05206:MicroRNAs in cancer	9	10.11	0.01314	Q00987, P33527, P08183, P08581, P42574, Q16678, P14780, P00749, P00533
KEGG_PATHWAY	hsa05210:Colorectal cancer	8	8.99	4.6E-06	P27986, P15153, P28482, P48736, P42574, P31749, P15056, P45983
KEGG_PATHWAY	hsa05212:Pancreatic cancer	8	8.99	6.3E-06	P27986, P15153, P28482, P48736, P00533, P31749, P15056, P45983
KEGG_PATHWAY	hsa05214:Glioma	8	8.99	6.3E-06	P27986, P05019, Q00987, P28482, P48736, P00533, P31749, P15056
KEGG_PATHWAY	hsa04664:Fc epsilon RI signaling	8	8.99	8.6E-06	P27986, O15530, P15153, P28482, P48736, Q16539, P31749, P45983

KEGG_PATHWAY	hsa04520:Adherens junction	8	8.99	1.1E-05	P12931, P15153, P11362, P08581, P28482, P06213, P00533, P18031
KEGG_PATHWAY	hsa04917:Prolactin signaling pathway	8	8.99	1.1E-05	P27986, P03372, P12931, P28482, P48736, Q16539, P31749, P45983
KEGG_PATHWAY	hsa04012:ErbB signaling pathway	8	8.99	4.4E-05	P27986, P12931, P28482, P48736, P00533, P31749, P15056, P45983
KEGG_PATHWAY	hsa05231:Choline metabolism in	8	8.99	0.00011	P27986, O15530, P15153, P28482, P48736, P00533, P31749, P45983
KEGG_PATHWAY	hsa05142:Chagas disease (American trypanosomiasis)	8	8.99	0.00014	P60568, P27986, P28482, P48736, Q16539, P35228, P31749, P45983
KEGG_PATHWAY	hsa04668:TNF signaling pathway	8	8.99	0.00016	P27986, P28482, P48736, P42574, P14780, Q16539, P31749, P45983
KEGG_PATHWAY	hsa04919:Thyroid hormone signaling pathway	8	8.99	0.00026	P27986, P03372, P12931, O15530, Q00987, P28482, P48736, P31749
KEGG_PATHWAY	hsa04152:AMPK signaling pathway	8	8.99	0.00039	P27986, P05019, O15530, P48736, P04035, P06213, P37231, P31749
KEGG_PATHWAY	hsa04611:Platelet activation	8	8.99	0.00054	P27986, Q15746, P12931, P29474, P28482, P48736, Q16539, P31749
KEGG_PATHWAY	hsa04932:Non-alcoholic fatty liver disease (NAFLD)	8	8.99	0.00132	P27986, Q13133, P48736, P42574, P06213, Q07869, P31749, P45983
KEGG_PATHWAY	hsa05164:Influenza A	8	8.99	0.00296	P27986, P11142, P28482, P48736, Q16539, P00747, P31749, P45983
KEGG_PATHWAY	hsa04024:cAMP signaling pathway	8	8.99	0.00601	P27986, P15153, P28482, P48736, Q07869, P31749, P15056, P45983
KEGG_PATHWAY	hsa05219:Bladder cancer	7	7.87	4.9E-06	P12931, Q00987, P08253, P28482, P14780, P00533, P15056
KEGG_PATHWAY	hsa05213:Endometrial cancer	7	7.87	2E-05	P27986, O15530, P28482, P48736, P00533, P31749, P15056
KEGG_PATHWAY	hsa05223:Non-small cell lung	7	7.87	3.1E-05	P27986, O15530, P28482, P48736, P00533, P31749, P15056
KEGG_PATHWAY	hsa04150:mTOR signaling pathway	7	7.87	3.8E-05	P27986, P05019, O15530, P28482, P48736, P31749, P15056
KEGG_PATHWAY	hsa05211:Renal cell carcinoma	7	7.87	8E-05	P27986, P08581, Q06124, P28482, P48736, P31749, P15056
KEGG_PATHWAY	hsa05120:Epithelial cell signaling in Helicobacter pylori infection	7	7.87	8.7E-05	P12931, P08581, Q06124, P42574, Q16539, P00533, P45983
KEGG_PATHWAY	hsa05220:Chronic myeloid leukemia	7	7.87	0.00013	P27986, Q00987, Q06124, P28482, P48736, P31749, P15056
KEGG_PATHWAY	hsa04660:T cell receptor signaling pathway	7	7.87	0.00078	P60568, P27986, O15530, P28482, P48736, Q16539, P31749
KEGG_PATHWAY	hsa04726:Serotonergic synapse	7	7.87	0.00135	P09917, P28482, P21397, P42574, P05067, P11712, P15056
KEGG_PATHWAY	hsa04670:Leukocyte transendothelial migration	7	7.87	0.00162	P27986, P15153, P08253, Q06124, P48736, P14780, Q16539
KEGG_PATHWAY	hsa04650:Natural killer cell mediated cytotoxicity	7	7.87	0.00219	P27986, P15153, Q06124, P28482, P48736, P42574, P15056
KEGG_PATHWAY	hsa05169:Epstein-Barr virus infection	7	7.87	0.00219	P27986, Q00987, P48736, Q16539, P24941, P31749, P45983
KEGG_PATHWAY	hsa04380:Osteoclast differentiation	7	7.87	0.00313	P27986, P28482, P48736, Q16539, P37231, P31749, P45983

KEGG_PATHWAY	hsa05152:Tuberculosis	7	7.87	0.01317	P12931, P28482, P42574, Q16539, P35228, P31749, P45983
KEGG_PATHWAY	hsa04062:Chemokine signaling pathway	7	7.87	0.01647	P27986, P12931, P15153, P28482, P48736, P31749, P15056
KEGG_PATHWAY	hsa05203:Viral carcinogenesis	7	7.87	0.02527	P27986, P12931, Q00987, P28482, P48736, P42574, P24941
KEGG_PATHWAY	hsa04960:Aldosterone-regulated sodium reabsorption	6	6.74	6.2E-05	P27986, P05019, O15530, P28482, P48736, P06213
KEGG_PATHWAY	hsa05221:Acute myeloid leukemia	6	6.74	0.00035	P27986, P36888, P28482, P48736, P31749, P15056
KEGG_PATHWAY	hsa04610:Complement and coagulation cascades	6	6.74	0.00093	P00742, P01009, P00749, P00747, P00734, P08709
KEGG_PATHWAY	hsa04912:GnRH signaling pathway	6	6.74	0.0032	P12931, P08253, P28482, Q16539, P00533, P45983
KEGG_PATHWAY	hsa04750:Inflammatory mediator regulation of TRP channels	6	6.74	0.0044	P27986, P05019, P12931, P48736, Q16539, P45983
KEGG_PATHWAY	hsa04620:Toll-like receptor signaling pathway	6	6.74	0.00614	P27986, P28482, P48736, Q16539, P31749, P45983
KEGG_PATHWAY	hsa05162:Measles	6	6.74	0.01548	P60568, P27986, P11142, P48736, P24941, P31749
KEGG_PATHWAY	hsa04921:Oxytocin signaling pathway	6	6.74	0.02468	P28907, Q15746, P12931, P29474, P28482, P00533
KEGG_PATHWAY	hsa04930:Type II diabetes mellitus	5	5.62	0.00185	P27986, P28482, P48736, P06213, P45983
KEGG_PATHWAY	hsa04976:Bile secretion	5	5.62	0.00692	P08183, Q96RI1, Q9UNQ0, P04035, Q06520
KEGG_PATHWAY	hsa04662:B cell receptor signaling pathway	5	5.62	0.00692	P27986, P15153, P28482, P48736, P31749
KEGG_PATHWAY	hsa00980:Metabolism of xenobiotics by cytochrome P450	5	5.62	0.00883	P09211, Q16678, P11712, Q06520, Q04828
KEGG_PATHWAY	hsa05133:Pertussis	5	5.62	0.00925	P28482, P42574, Q16539, P35228, P45983
KEGG_PATHWAY	hsa04666:Fc gamma R-mediated phagocytosis	5	5.62	0.01364	P27986, P15153, P28482, P48736, P31749
KEGG_PATHWAY	hsa05222:Small cell lung cancer	5	5.62	0.01419	P27986, P48736, P24941, P35228, P31749
KEGG_PATHWAY	hsa05146:Amoebiasis	5	5.62	0.02931	P27986, P05089, P48736, P42574, P35228
KEGG_PATHWAY	hsa04725:Cholinergic synapse	5	5.62	0.03393	P27986, P28482, P48736, P22303, P31749
KEGG_PATHWAY	hsa04913:Ovarian steroidogenesis	4	4.49	0.01659	P05019, P09917, Q16678, P06213
KEGG_PATHWAY	hsa00330:Arginine and proline metabolism	4	4.49	0.01752	P05089, P29474, P21397, P35228
KEGG_PATHWAY	hsa04923:Regulation of lipolysis in adipocytes	4	4.49	0.02364	P27986, P48736, P06213, P31749

KEGG_PATHWAY	hsa04621:NOD-like receptor signaling pathway	4	4.49	0.02364	P07900, P28482, Q16539, P45983
KEGG_PATHWAY	hsa04210:Apoptosis	4	4.49	0.03078	P27986, P48736, P42574, P31749
KEGG_PATHWAY	hsa05131:Shigellosi	4	4.49	0.03338	P12931, P28482, Q16539, P45983
KEGG_PATHWAY	hsa04115:p53 signaling pathway	4	4.49	0.0375	P05019, Q00987, P42574, P24941
KEGG_PATHWAY	hsa03320:PPAR signaling pathway	4	4.49	0.0375	Q13133, O15530, P37231, Q07869
KEGG_PATHWAY	hsa04920:Adipocytokine signaling pathway	4	4.49	0.04186	Q06124, Q07869, P31749, P45983
KEGG_PATHWAY	hsa00220:Arginine biosynthesis	3	3.37	0.02013	P05089, P29474, P35228
KEGG_PATHWAY	hsa05216:Thyroid cancer	3	3.37	0.04035	P28482, P37231, P15056
GOTERM_BP_DIREC	GO:0043066~negative regulation of apoptotic process	22	24.72	1.5E-14	P05019, P12931, P02768, Q04760, P14780, P14174, P05164, P28907, P60568, P27986, P35968, Q00987, P09211, P04179, Q96RI1, P42574, P15559, P08758, P00533, P31749, P45983, P15056
GOTERM_BP_DIREC	GO:0045944~positive regulation of transcription from RNA polymerase II promoter	19	21.35	2.7E-06	Q13133, P05019, P08581, O75469, P05067, P09874, P35398, P60568, P27986, P03372, Q96RI1, P21802, P37231, Q16539, P00533, Q07869, P12643, P31749, P55055
GOTERM_BP_DIREC	GO:0007165~signal transduction	17	19.10	0.00031	P05019, P12931, P08581, O75469, P28907, P03372, P27986, P07900, P15153, Q96RI1, P28482, P00749, P37231, P08758, Q16539, P00533, P31749
GOTERM_BP_DIREC	GO:0055114~oxidation-reduction process	16	17.98	4E-07	P09917, P47989, P00390, P35228, P04035, P11712, P05164, P30043, P04179, P29474, P21397, Q16678, P15559, P12643, Q04828, P00374
GOTERM_BP_DIREC	GO:0045893~positive regulation of transcription, DNA-templated	14	15.73	2.7E-06	P05019, Q13133, P12931, O75469, P24941, P35398, P28907, P03372, P28482, P37231, P06213, Q07869, P12643, P55055
GOTERM_BP_DIREC	GO:0042493~response to drug	13	14.61	6E-08	P28907, P07900, P12931, Q00987, P33527, P04179, P08183, P05089, P04818, Q9UNQ0, P42574, P37231, P06276
GOTERM_BP_DIREC	GO:0006468~protein phosphorylation	13	14.61	4.4E-06	P27986, Q15746, O15530, Q96GD4, P11362, P28482, P48736, O60285, P05067, P12643, P31749, P15056, P45983
GOTERM_BP_DIREC	GO:0008284~positive regulation of cell proliferation	13	14.61	5.5E-06	P60568, P05019, P36888, P35968, Q00987, P15153, P11362, P28482, P21802, P06213, P24941, P00533, P00734
GOTERM_BP_DIREC	GO:0000122~negative regulation of transcription from RNA polymerase II promoter	13	14.61	0.00036	Q13133, P11362, P09874, P08246, P03372, Q00987, Q96GD4, Q96RI1, P21802, P37231, Q07869, P12643, P55055

GOTERM_BP_DIREC	GO:0030335~positive regulation of cell migration	11	12.36	4.2E-08	P27986, P00742, P05019, P35968, Q15746, P28482, P00749, P06213, P00533, P12643, P08709
GOTERM_BP_DIREC	GO:0046777~protein autophosphorylation	10	11.24	2.9E-07	P36888, P35968, P12931, O15530, Q96GD4, P11362, P21802, P06213, P00533, P31749
GOTERM_BP_DIREC	GO:0001666~response to hypoxia	10	11.24	2.9E-07	P28907, P04179, P08253, P03950, P42574, P00749, P35228, Q07869, P12643, P08709
GOTERM_BP_DIREC	GO:0001525~angiogenesis	10	11.24	2.5E-06	P35968, P11362, P29474, P08253, P21802, P48736, P03950, Q16678, Q16539, P35398
GOTERM_BP_DIREC	GO:0048015~phosphatidylinositol-mediated signaling	9	10.11	8.2E-08	P27986, P05019, O15530, P11362, Q06124, P21802, P48736, P00533, P31749
GOTERM_BP_DIREC	GO:0030168~platelet activation	9	10.11	1.5E-07	P27986, P12931, O15530, P15153, Q06124, P28482, P48736, P00734, P31749
GOTERM_BP_DIREC	GO:0018108~peptidyl-tyrosine phosphorylation	9	10.11	1.4E-06	P07900, P36888, P35968, P12931, P11362, P08581, P21802, P06213, P00533
GOTERM_BP_DIREC	GO:0070374~positive regulation of ERK1 and ERK2	9	10.11	3.7E-06	P35968, P12931, Q06124, P21802, P14174, P04035, P00533, P12643, P15056
GOTERM_BP_DIREC	GO:0008283~cell proliferation	9	10.11	0.00067	P05019, P12931, Q96GD4, P04818, P08581, P22303, P14174, P00533, P31749
GOTERM_BP_DIREC	GO:0006508~proteolysis	9	10.11	0.00468	P08246, P08253, P42574, P14780, P00749, P00747, P00734, P39900, P00797
GOTERM_BP_DIREC	GO:0043401~steroid hormone mediated signaling pathway	8	8.99	1.8E-08	Q13133, P03372, Q96RI1, O75469, P37231, Q07869, P35398, P55055
GOTERM_BP_DIREC	GO:0032869~cellular response to insulin stimulus	8	8.99	1.5E-07	P27986, P12931, O15530, P09211, P06213, P37231, P09874, P31749
GOTERM_BP_DIREC	GO:0006805~xenobiotic metabolic process	8	8.99	1.7E-07	P09211, P23141, Q16678, O75469, P15559, P11712, P35398, Q04828
GOTERM_BP_DIREC	GO:0009636~response to toxic	8	8.99	3E-07	Q00987, P09211, P04818, P28482, P23141, P49638, Q16678, P15559
GOTERM_BP_DIREC	GO:0071222~cellular response to lipopolysaccharide	8	8.99	2.1E-06	Q13133, P12931, P09211, P05089, Q96RI1, Q16539, P35228, P45983
GOTERM_BP_DIREC	GO:0006367~transcription initiation from RNA polymerase II	8	8.99	1.5E-05	Q13133, P03372, Q96RI1, O75469, P37231, Q07869, P35398, P55055
GOTERM_BP_DIREC	GO:0045087~innate immune response	8	8.99	0.00736	P12931, Q96RI1, P48736, P03950, Q07912, P14174, P05067, P37231
GOTERM_BP_DIREC	GO:0045892~negative regulation of transcription, DNA-templated	8	8.99	0.01576	P28907, P11142, P12931, Q00987, O75469, P37231, P12643, P55055
GOTERM_BP_DIREC	GO:0043406~positive regulation of MAP kinase activity	7	7.87	6.3E-07	P08246, P36888, P12931, P11362, P48736, P14174, P00533

GOTERM_BP_DIREC	GO:0014066~regulation of phosphatidylinositol 3-kinase signaling	7	7.87	3.3E-06	P27986, P11362, Q06124, P28482, P21802, P00533, P31749
GOTERM_BP_DIREC	GO:0043410~positive regulation of MAPK cascade	7	7.87	4.1E-06	P05019, P36888, P35968, P11362, P21802, P06213, P12643
GOTERM_BP_DIREC	GO:0042593~glucose homeostasis	7	7.87	1.5E-05	P02753, Q96RI1, Q06124, P06213, P37231, P06737, P31749
GOTERM_BP_DIREC	GO:0050900~leukocyte migration	7	7.87	4.3E-05	P27986, P12931, Q06124, P14780, P14174, P16109, P00734
GOTERM_BP_DIREC	GO:0018105~peptidyl-serine phosphorylation	7	7.87	5E-05	P12931, O15530, P28482, Q16539, P24941, P31749, P45983
GOTERM_BP_DIREC	GO:0001934~positive regulation of protein phosphorylation	7	7.87	5.4E-05	P35968, P14780, P06213, P00533, P12643, P00734, P31749
GOTERM_BP_DIREC	GO:0007568~aging	7	7.87	0.00023	P05164, Q96GD4, P05089, P04818, P04035, P15559, P31749
GOTERM_BP_DIREC	GO:0007596~blood coagulation	7	7.87	0.00041	P00742, P01009, P08758, P00749, P00747, P00734, P08709
GOTERM_BP_DIREC	GO:0006809~nitric oxide biosynthetic process	6	6.74	4.4E-09	P29474, Q16678, P15559, P35228, P35398, P31749
GOTERM_BP_DIREC	GO:0045429~positive regulation of nitric oxide biosynthetic process	6	6.74	2.9E-06	P07900, P03372, P04179, P06213, P00533, P31749
GOTERM_BP_DIREC	GO:0048661~positive regulation of smooth muscle cell proliferation	6	6.74	1.5E-05	P08246, P05019, P21802, P04035, P00533, P31749
GOTERM_BP_DIREC	GO:0014068~positive regulation of phosphatidylinositol 3-kinase signaling	6	6.74	2.3E-05	P05019, P36888, P35968, P11362, P16109, P00734
GOTERM_BP_DIREC	GO:0050728~negative regulation of inflammatory response	6	6.74	5.9E-05	P08246, P60568, Q13133, Q96RI1, Q07869, P35398
GOTERM_BP_DIREC	GO:0051897~positive regulation of protein kinase B signaling	6	6.74	7.9E-05	P00742, P12931, P48736, P06213, P00533, P08709
GOTERM_BP_DIREC	GO:0032355~response to estradiol	6	6.74	0.00011	P28907, P03372, P09211, P42574, P15559, P08709
GOTERM_BP_DIREC	GO:0046854~phosphatidylinositol phosphorylation	6	6.74	0.00013	P27986, P11362, Q06124, P21802, P48736, P00533
GOTERM_BP_DIREC	GO:0007169~transmembrane receptor protein tyrosine kinase signaling pathway	6	6.74	0.00015	P36888, P35968, P08581, Q07912, P06213, P00533
GOTERM_BP_DIREC	GO:0002576~platelet degranulation	6	6.74	0.00021	P05019, P02768, P01009, P16109, P05067, P00747

GOTERM_BP_DIREC	GO:0045471~response to ethanol	6	6.74	0.00023	P60568, P02753, P09211, P04818, P04035, P15559
GOTERM_BP_DIREC	GO:0000187~activation of MAPK	6	6.74	0.00025	P05019, Q06124, P28482, Q16539, P06213, P12643
GOTERM_BP_DIREC	GO:0044267~cellular protein metabolic process	6	6.74	0.00039	P05019, P08253, P02766, P05067, P00747, P00734
GOTERM_BP_DIREC	GO:1901796~regulation of signal transduction by p53 class mediator	6	6.74	0.00049	Q00987, Q96GD4, O60285, Q16539, P24941, P31749
GOTERM_BP_DIREC	GO:0032496~response to lipopolysaccharide	6	6.74	0.00171	P05164, P08246, P04179, P42574, P16109, P00797
GOTERM_BP_DIREC	GO:0007507~heart development	6	6.74	0.00275	P02753, P04179, Q06124, P37231, Q07869, P12643
GOTERM_BP_DIREC	GO:0042127~regulation of cell proliferation	6	6.74	0.00288	P12931, O60285, Q07912, P00749, P35228, P15056
GOTERM_BP_DIREC	GO:0010628~positive regulation of gene expression	6	6.74	0.0122	Q00987, Q16539, O75469, P12643, P15056, P45983
GOTERM_BP_DIREC	GO:0000165~MAPK cascade	6	6.74	0.0122	P60568, P11362, P28482, P21802, P00533, P15056
GOTERM_BP_DIREC	GO:0007166~cell surface receptor signaling pathway	6	6.74	0.01457	P08581, Q07912, P14174, Q16539, P00533, P00734
GOTERM_BP_DIREC	GO:0006810~transport	6	6.74	0.03604	P02768, P33527, P08183, P02766, Q9UNQ0, P49638
GOTERM_BP_DIREC	GO:0006954~inflammatory response	6	6.74	0.04891	Q96RI1, P48736, P14174, P16109, P12643, P31749
GOTERM_BP_DIREC	GO:0032148~activation of protein kinase B activity	5	5.62	9.6E-06	P05019, P12931, O15530, P03950, P06213
GOTERM_BP_DIREC	GO:0046326~positive regulation of glucose import	5	5.62	1.7E-05	P27986, P05019, Q16539, P06213, P31749
GOTERM_BP_DIREC	GO:0038128~ERBB2 signaling	5	5.62	4.5E-05	P07900, P27986, P12931, P00533, P31749
GOTERM_BP_DIREC	GO:0030522~intracellular receptor signaling pathway	5	5.62	4.5E-05	Q13133, Q96RI1, O75469, Q07869, P35398
GOTERM_BP_DIREC	GO:0036092~phosphatidylinositol-3-phosphate biosynthetic process	5	5.62	0.00012	P27986, P11362, Q06124, P21802, P48736
GOTERM_BP_DIREC	GO:0007173~epidermal growth factor receptor signaling pathway	5	5.62	0.00021	P27986, P12931, O15530, Q06124, P00533
GOTERM_BP_DIREC	GO:0043627~response to estrogen	5	5.62	0.00037	P07900, P03372, P28482, P37231, P08709
GOTERM_BP_DIREC	GO:0001938~positive regulation of endothelial cell proliferation	5	5.62	0.00047	P35968, P05089, P03950, P12643, P31749

GOTERM_BP_DIREC	GO:0048010~vascular endothelial growth factor receptor signaling	5	5.62	0.00055	P07900, P27986, P35968, P12931, Q16539
GOTERM_BP_DIREC	GO:0030324~lung development	5	5.62	0.00068	P02753, P05089, P29474, P21802, P00533
GOTERM_BP_DIREC	GO:0022617~extracellular matrix disassembly	5	5.62	0.00068	P08246, P08253, P14780, P00747, P39900
GOTERM_BP_DIREC	GO:0031295~T cell costimulation	5	5.62	0.00075	P27986, P12931, O15530, Q06124, P31749
GOTERM_BP_DIREC	GO:0030307~positive regulation of cell growth	5	5.62	0.00099	P28907, P60568, P00533, P00734, P31749
GOTERM_BP_DIREC	GO:0010629~negative regulation of gene expression	5	5.62	0.00583	P03372, P47989, P14174, P35228, P31749
GOTERM_BP_DIREC	GO:0010887~negative regulation of cholesterol storage	4	4.49	2.8E-06	Q13133, P37231, Q07869, P55055
GOTERM_BP_DIREC	GO:2000188~regulation of cholesterol homeostasis	4	4.49	1.1E-05	Q13133, Q96RI1, P35398, P55055
GOTERM_BP_DIREC	GO:0019430~removal of superoxide radicals	4	4.49	3E-05	P05164, P04179, P29474, P15559
GOTERM_BP_DIREC	GO:0010745~negative regulation of macrophage derived foam cell differentiation	4	4.49	3.8E-05	Q13133, P37231, Q07869, P55055
GOTERM_BP_DIREC	GO:0001878~response to yeast	4	4.49	3.8E-05	P05164, P08246, P03950, P05067
GOTERM_BP_DIREC	GO:0045861~negative regulation of proteolysis	4	4.49	0.00017	Q13133, P00734, P55055, P31749
GOTERM_BP_DIREC	GO:0050999~regulation of nitric-oxide synthase activity	4	4.49	0.00033	P07900, P29474, P00533, P31749
GOTERM_BP_DIREC	GO:0040014~regulation of multicellular organism growth	4	4.49	0.00033	P05019, Q06124, P21802, P05067
GOTERM_BP_DIREC	GO:0031663~lipopolysaccharide-mediated signaling	4	4.49	0.00062	P29474, P28482, Q16539, P31749
GOTERM_BP_DIREC	GO:0071364~cellular response to epidermal growth factor stimulus	4	4.49	0.00068	O15530, P09211, P00533, P31749
GOTERM_BP_DIREC	GO:0018107~peptidyl-threonine phosphorylation	4	4.49	0.00103	O15530, P28482, P31749, P45983
GOTERM_BP_DIREC	GO:0038083~peptidyl-tyrosine autophosphorylation	4	4.49	0.00119	P35968, P12931, Q07912, P06213

GOTERM_BP_DIREC	GO:0043124~negative regulation of I-kappaB kinase/NF-kappaB signaling	4	4.49	0.00119	P03372, P09211, Q96RI1, P35398
GOTERM_BP_DIREC	GO:0008202~steroid metabolic process	4	4.49	0.00148	Q16678, O75469, P11712, Q06520
GOTERM_BP_DIREC	GO:0031100~organ regeneration	4	4.49	0.00191	P36888, P09211, P37231, P08709
GOTERM_BP_DIREC	GO:0009408~response to heat	4	4.49	0.00203	P07900, P05019, P29474, P31749
GOTERM_BP_DIREC	GO:0042752~regulation of circadian rhythm	4	4.49	0.00215	Q13133, P37231, Q07869, P45983
GOTERM_BP_DIREC	GO:0048146~positive regulation of fibroblast proliferation	4	4.49	0.00284	P05019, P03372, P14174, P00533
GOTERM_BP_DIREC	GO:0006749~glutathione metabolic process	4	4.49	0.00315	P09211, P00390, P04179, Q04760
GOTERM_BP_DIREC	GO:0071407~cellular response to organic cyclic	4	4.49	0.00366	P42574, Q16678, P12643, P31749
GOTERM_BP_DIREC	GO:0006950~response to stress	4	4.49	0.00402	P07900, P28482, P00533, P45983
GOTERM_BP_DIREC	GO:0008217~regulation of blood	4	4.49	0.00481	P04179, P29474, P37231, P00797
GOTERM_BP_DIREC	GO:0035690~cellular response to drug	4	4.49	0.00568	P35228, P00533, P00797, P15056
GOTERM_BP_DIREC	GO:0030855~epithelial cell	4	4.49	0.00591	P21802, P23141, P37231, Q04828
GOTERM_BP_DIREC	GO:0007584~response to nutrient	4	4.49	0.0069	P49638, P04035, P37231, P15559
GOTERM_BP_DIREC	GO:0007623~circadian rhythm	4	4.49	0.00716	P04818, P35228, P00533, P08709
GOTERM_BP_DIREC	GO:0008286~insulin receptor signaling pathway	4	4.49	0.00797	P27986, P06213, P18031, P31749
GOTERM_BP_DIREC	GO:0008543~fibroblast growth factor receptor signaling pathway	4	4.49	0.00914	P11362, Q06124, P28482, P21802
GOTERM_BP_DIREC	GO:0050731~positive regulation of peptidyl-tyrosine phosphorylation	4	4.49	0.00914	P05019, P12931, Q07912, P14174
GOTERM_BP_DIREC	GO:0048013~ephrin receptor signaling pathway	4	4.49	0.01041	P12931, P08253, Q06124, P14780
GOTERM_BP_DIREC	GO:0071456~cellular response to	4	4.49	0.014	P12931, Q00987, P35398, P31749
GOTERM_BP_DIREC	GO:0006979~response to oxidative	4	4.49	0.02007	P05164, P05067, P00533, P31749
GOTERM_BP_DIREC	GO:0006935~chemotaxis	4	4.49	0.02626	P15153, P28482, Q16539, P00749

GOTERM_BP_DIREC	GO:0038096~Fc-gamma receptor signaling pathway involved in phagocytosis	4	4.49	0.02911	P07900, P27986, P12931, P28482
GOTERM_BP_DIREC	GO:0007204~positive regulation of cytosolic calcium ion concentration	4	4.49	0.03336	P28907, P60568, P03372, P48736
GOTERM_BP_DIREC	GO:0001501~skeletal system	4	4.49	0.03528	P05019, P11362, P14780, P12643
GOTERM_BP_DIREC	GO:0042742~defense response to bacterium	4	4.49	0.04066	P05164, P08246, Q96RI1, P35228
GOTERM_BP_DIREC	GO:0060336~negative regulation of interferon-gamma-mediated signaling pathway	3	3.37	0.00027	Q13133, P37231, P55055
GOTERM_BP_DIREC	GO:0042908~xenobiotic transport	3	3.37	0.00056	P08183, Q9UNQ0, O75469
GOTERM_BP_DIREC	GO:0048762~mesenchymal cell differentiation	3	3.37	0.00074	P11362, P21802, P12643
GOTERM_BP_DIREC	GO:0006527~arginine catabolic process	3	3.37	0.00074	P05089, P29474, P35228
GOTERM_BP_DIREC	GO:0010518~positive regulation of phospholipase activity	3	3.37	0.00074	O15530, P11362, P21802
GOTERM_BP_DIREC	GO:0033628~regulation of cell adhesion mediated by integrin	3	3.37	0.00145	Q06124, P48736, P00749
GOTERM_BP_DIREC	GO:0017187~peptidyl-glutamic acid carboxylation	3	3.37	0.00145	P00742, P00734, P08709
GOTERM_BP_DIREC	GO:2001275~positive regulation of glucose import in response to insulin stimulus	3	3.37	0.00204	P27986, Q96RI1, Q06124
GOTERM_BP_DIREC	GO:0060065~uterus development	3	3.37	0.00237	P03372, P02753, P12931
GOTERM_BP_DIREC	GO:0048009~insulin-like growth factor receptor signaling pathway	3	3.37	0.00237	P27986, P05019, P31749
GOTERM_BP_DIREC	GO:0043254~regulation of protein complex assembly	3	3.37	0.00237	P07900, P11142, Q06124
GOTERM_BP_DIREC	GO:0032270~positive regulation of cellular protein metabolic process	3	3.37	0.00273	Q13133, P55055, P31749

GOTERM_BP_DIREC	GO:1904707~positive regulation of vascular smooth muscle cell proliferation	3	3.37	0.00273	Q00987, P08253, P14780
GOTERM_BP_DIREC	GO:0045725~positive regulation of glycogen biosynthetic process	3	3.37	0.00273	P05019, P06213, P31749
GOTERM_BP_DIREC	GO:0048011~neurotrophin TRK receptor signaling	3	3.37	0.00311	P12931, Q06124, P42574
GOTERM_BP_DIREC	GO:0033189~response to vitamin A	3	3.37	0.00393	P05089, P04818, P37231
GOTERM_BP_DIREC	GO:1900182~positive regulation of protein localization to nucleus	3	3.37	0.00534	P12931, P00734, P31749
GOTERM_BP_DIREC	GO:0035902~response to immobilization	3	3.37	0.00534	P04179, P37231, P00797
GOTERM_BP_DIREC	GO:0042730~fibrinolysis	3	3.37	0.00534	P00749, P00747, P00734
GOTERM_BP_DIREC	GO:0051000~positive regulation of nitric-oxide synthase activity	3	3.37	0.00586	P03372, P31749, P00374
GOTERM_BP_DIREC	GO:0060045~positive regulation of cardiac muscle cell proliferation	3	3.37	0.00586	P11362, P21802, Q16539
GOTERM_BP_DIREC	GO:0035924~cellular response to vascular endothelial growth factor stimulus	3	3.37	0.0064	P35968, Q16539, P31749
GOTERM_BP_DIREC	GO:0051090~regulation of sequence-specific DNA binding transcription factor	3	3.37	0.00753	P28482, Q16539, P45983
GOTERM_BP_DIREC	GO:0006465~signal peptide processing	3	3.37	0.00753	P00742, P00734, P08709
GOTERM_BP_DIREC	GO:0042327~positive regulation of phosphorylation	3	3.37	0.00753	P03950, P14174, P00533
GOTERM_BP_DIREC	GO:0015721~bile acid and bile salt transport	3	3.37	0.00875	P02768, Q96RI1, Q04828
GOTERM_BP_DIREC	GO:0014065~phosphatidylinositol 3-kinase signaling	3	3.37	0.00875	P27986, P05019, P48736
GOTERM_BP_DIREC	GO:0045931~positive regulation of mitotic cell cycle	3	3.37	0.00939	Q00987, Q06124, P05067
GOTERM_BP_DIREC	GO:0045909~positive regulation of vasodilation	3	3.37	0.01006	P29474, P35228, P00533

GOTERM_BP_DIREC	GO:2000379~positive regulation of reactive oxygen species metabolic process	3	3.37	0.01074	P47989, Q16539, P00734
GOTERM_BP_DIREC	GO:0045776~negative regulation of blood pressure	3	3.37	0.01074	P29474, P35228, Q07869
GOTERM_BP_DIREC	GO:0042572~retinol metabolic process	3	3.37	0.01074	P02753, P02766, Q16678
GOTERM_BP_DIREC	GO:0090004~positive regulation of establishment of protein localization to plasma membrane	3	3.37	0.01074	P27986, O15530, P31749
GOTERM_BP_DIREC	GO:0043200~response to amino acid	3	3.37	0.01144	P09211, P05089, P42574
GOTERM_BP_DIREC	GO:0046677~response to antibiotic	3	3.37	0.01216	P07900, Q00987, P42574
GOTERM_BP_DIREC	GO:0045907~positive regulation of vasoconstriction	3	3.37	0.01216	P28907, P00533, P31749
GOTERM_BP_DIREC	GO:0001890~placenta development	3	3.37	0.01444	P03950, Q16539, P37231
GOTERM_BP_DIREC	GO:0031648~protein destabilization	3	3.37	0.01444	P12931, Q00987, P12643
GOTERM_BP_DIREC	GO:0009409~response to cold	3	3.37	0.01524	P07900, P04179, P37231
GOTERM_BP_DIREC	GO:0043407~negative regulation of MAP kinase activity	3	3.37	0.01524	P09211, P04035, P18031
GOTERM_BP_DIREC	GO:0050714~positive regulation of protein secretion	3	3.37	0.01524	P05019, P03950, P22303
GOTERM_BP_DIREC	GO:0048469~cell maturation	3	3.37	0.01524	P11362, P37231, P00797
GOTERM_BP_DIREC	GO:2001237~negative regulation of extrinsic apoptotic signaling pathway	3	3.37	0.01689	P05019, P12931, P09211
GOTERM_BP_DIREC	GO:0000302~response to reactive oxygen species	3	3.37	0.01774	P09211, P00390, P04179
GOTERM_BP_DIREC	GO:0030890~positive regulation of B cell proliferation	3	3.37	0.01774	P28907, P60568, P14174
GOTERM_BP_DIREC	GO:0002062~chondrocyte	3	3.37	0.01774	P11362, Q16539, P12643
GOTERM_BP_DIREC	GO:0032570~response to progesterone	3	3.37	0.01774	P28907, Q13133, P04818
GOTERM_BP_DIREC	GO:0030163~protein catabolic process	3	3.37	0.0195	P08246, P31749, P00797
GOTERM_BP_DIREC	GO:0044255~cellular lipid metabolic process	3	3.37	0.0195	Q13133, Q07869, P55055
GOTERM_BP_DIREC	GO:0032526~response to retinoic acid	3	3.37	0.0195	P28907, P02753, P37231

GOTERM_BP_DIREC	GO:0045740~positive regulation of DNA replication	3	3.37	0.02041	P05019, P06213, P00533
GOTERM_BP_DIREC	GO:0008542~visual learning	3	3.37	0.02323	P04035, P05067, P15056
GOTERM_BP_DIREC	GO:0045165~cell fate commitment	3	3.37	0.02421	P21802, P37231, P12643
GOTERM_BP_DIREC	GO:0045600~positive regulation of fat cell differentiation	3	3.37	0.0252	P37231, P12643, P31749
GOTERM_BP_DIREC	GO:0042542~response to hydrogen peroxide	3	3.37	0.02932	P12931, P04179, P42574
GOTERM_BP_DIREC	GO:0006417~regulation of translation	3	3.37	0.03039	P04818, P05067, P31749
GOTERM_BP_DIREC	GO:0050829~defense response to Gram-negative	3	3.37	0.0337	P16109, P05067, P35228
GOTERM_BP_DIREC	GO:0070301~cellular response to hydrogen peroxide	3	3.37	0.03598	Q00987, P05089, Q16678
GOTERM_BP_DIREC	GO:0032091~negative regulation of protein binding	3	3.37	0.03598	Q96GD4, Q07869, P45983
GOTERM_BP_DIREC	GO:0009612~response to mechanical stimulus	3	3.37	0.03831	P05164, P12931, P37231
GOTERM_BP_DIREC	GO:0050679~positive regulation of epithelial cell proliferation	3	3.37	0.0395	P05019, P21802, P00533
GOTERM_BP_DIREC	GO:0001523~retinoid metabolic process	3	3.37	0.0407	P02753, P02766, Q04828
GOTERM_BP_DIREC	GO:0030574~collagen catabolic process	3	3.37	0.04439	P08253, P14780, P39900
GOTERM_BP_DIREC	GO:0042632~cholesterol homeostasis	3	3.37	0.04439	Q13133, Q04828, P55055
GOTERM_BP_DIREC	GO:0051384~response to	3	3.37	0.04565	P04818, P42574, P06276
GOTERM_BP_DIREC	GO:0035607~fibroblast growth factor receptor signaling pathway involved in orbitofrontal cortex development	2	2.25	0.01045	P11362, P21802
GOTERM_BP_DIREC	GO:0070141~response to UV-A	2	2.25	0.01564	P00533, P31749
GOTERM_BP_DIREC	GO:0010871~negative regulation of receptor biosynthetic process	2	2.25	0.01564	P37231, Q07869
GOTERM_BP_DIREC	GO:0002679~respiratory burst involved in defense response	2	2.25	0.01564	P05164, P48736
GOTERM_BP_DIREC	GO:0021847~ventricular zone neuroblast division	2	2.25	0.01564	P11362, P21802

GOTERM_BP_DIREC	GO:0010641~positive regulation of platelet-derived growth factor receptor signaling pathway	2	2.25	0.01564	P12931, P08709
GOTERM_BP_DIREC	GO:0031281~positive regulation of cyclase activity	2	2.25	0.01564	Q16539, P45983
GOTERM_BP_DIREC	GO:0061684~chaperone-mediated autophagy	2	2.25	0.01564	P07900, P11142
GOTERM_BP_DIREC	GO:0060523~prostate epithelial cord elongation	2	2.25	0.01564	P03372, P21802
GOTERM_BP_DIREC	GO:0032376~positive regulation of cholesterol transport	2	2.25	0.0208	Q13133, P55055
GOTERM_BP_DIREC	GO:0046683~response to organophosphorus	2	2.25	0.0208	P04818, Q04828
GOTERM_BP_DIREC	GO:0032369~negative regulation of lipid transport	2	2.25	0.0208	Q13133, P55055
GOTERM_BP_DIREC	GO:0038127~ERBB signaling pathway	2	2.25	0.0208	Q06124, P28482
GOTERM_BP_DIREC	GO:0048550~negative regulation of pinocytosis	2	2.25	0.0208	Q13133, P55055
GOTERM_BP_DIREC	GO:1900015~regulation of cytokine production involved in inflammatory response	2	2.25	0.0208	Q16539, P35228
GOTERM_BP_DIREC	GO:0060850~regulation of transcription involved in cell fate commitment	2	2.25	0.02593	P37231, P35398
GOTERM_BP_DIREC	GO:0010891~negative regulation of sequestering of triglyceride	2	2.25	0.02593	P37231, Q07869
GOTERM_BP_DIREC	GO:0035630~bone mineralization involved in bone maturation	2	2.25	0.02593	P05019, P12643
GOTERM_BP_DIREC	GO:0048562~embryonic organ morphogenesis	2	2.25	0.02593	P02753, P21802
GOTERM_BP_DIREC	GO:0008088~axo-dendritic transport	2	2.25	0.02593	P10636, P05067
GOTERM_BP_DIREC	GO:0033591~response to L-ascorbic	2	2.25	0.02593	P09211, P04179
GOTERM_BP_DIREC	GO:0007598~blood coagulation, extrinsic pathway	2	2.25	0.02593	P00742, P08709
GOTERM_BP_DIREC	GO:0010042~response to manganese	2	2.25	0.03104	P05089, P04179

GOTERM_BP_DIREC	GO:0060527~prostate epithelial cord arborization involved in prostate glandular acinus morphogenesis	2	2.25	0.03104	P03372, P21802
GOTERM_BP_DIREC	GO:0071638~negative regulation of monocyte chemotactic protein-1 production	2	2.25	0.03104	P09211, Q96RI1
GOTERM_BP_DIREC	GO:0046321~positive regulation of fatty acid oxidation	2	2.25	0.03104	P37231, Q07869
GOTERM_BP_DIREC	GO:0035726~common myeloid progenitor cell	2	2.25	0.03612	P36888, P09211
GOTERM_BP_DIREC	GO:0002318~myeloid progenitor cell differentiation	2	2.25	0.03612	P36888, P15056
GOTERM_BP_DIREC	GO:0051593~response to folic acid	2	2.25	0.03612	P04818, P06276
GOTERM_BP_DIREC	GO:0060687~regulation of branching involved in prostate gland	2	2.25	0.03612	P03372, P21802
GOTERM_BP_DIREC	GO:0097011~cellular response to granulocyte macrophage colony-stimulating factor stimulus	2	2.25	0.04117	P28482, P31749
GOTERM_BP_DIREC	GO:0033629~negative regulation of cell adhesion mediated by integrin	2	2.25	0.04117	Q06124, Q16678
GOTERM_BP_DIREC	GO:0002674~negative regulation of acute inflammatory response	2	2.25	0.04117	P09211, P37231
GOTERM_BP_DIREC	GO:0010544~negative regulation of platelet activation	2	2.25	0.04117	P29474, P00734
GOTERM_BP_DIREC	GO:0010269~response to selenium ion	2	2.25	0.04117	P05089, P04179
GOTERM_BP_DIREC	GO:0032872~regulation of stress-activated MAPK cascade	2	2.25	0.04117	P09211, P28482
GOTERM_BP_DIREC	GO:0043304~regulation of mast cell degranulation	2	2.25	0.04117	O15530, P15153
GOTERM_BP_DIREC	GO:0002903~negative regulation of B cell apoptotic	2	2.25	0.04117	P60568, Q96GD4

GOTERM_BP_DIREC	GO:0031659~positive regulation of cyclin-dependent protein serine/threonine kinase activity involved in G1/S transition of mitotic cell cycle	2	2.25	0.04117	P00533, P31749
GOTERM_BP_DIREC	GO:2000641~regulation of early endosome to late endosome transport	2	2.25	0.04117	P12931, P28482
GOTERM_BP_DIREC	GO:0043030~regulation of macrophage activation	2	2.25	0.0462	P14174, P35398
GOTERM_BP_DIREC	GO:0034405~response to fluid shear stress	2	2.25	0.0462	P29474, P31749
GOTERM_BP_DIREC	GO:0060020~Bergmann glial cell differentiation	2	2.25	0.0462	Q06124, P28482
GOTERM_BP_DIREC	GO:0010907~positive regulation of glucose metabolic process	2	2.25	0.0462	P12931, P31749
GOTERM_BP_DIREC	GO:0060068~vagina development	2	2.25	0.0462	P03372, P02753
GOTERM_BP_DIREC	GO:0097267~omega-hydroxylase P450 pathway	2	2.25	0.0462	Q16678, P11712
GOTERM_BP_DIREC	GO:0010863~positive regulation of phospholipase C activity	2	2.25	0.0462	P03372, P11362
GOTERM_BP_DIREC	GO:0042473~outer ear morphogenesis	2	2.25	0.0462	P11362, P28482
GOTERM_BP_DIREC	GO:0032930~positive regulation of superoxide anion generation	2	2.25	0.0462	P09211, P00533
GOTERM_CC_DIREC	GO:0005829~cytosol	42	47.19	9.11E-10	P09917, P02753, P12931, P11362, P48736, P35228, P06737, Q06520, P07900, P30043, P11142, P27986, Q00987, O15530, P15153, Q96GD4, P29474, Q06124, P28482, P37231, P15559, Q04828, P15056, P00374, P10636, P36888, P47989, P05089, P00390, Q04760, P23141, P49638, P05067, P24941, P18031, Q15746, P09211, P04818,

GOTERM_CC_DIREC	GO:0005634~nucleus	42	47.19	6.00E-04	P12931, P11362, P35228, P09874, P05164, P07900, P11142, P27986, Q00987, Q96GD4, P29474, Q06124, Q96RI1, P28482, P21802, Q9UNQ0, P22303, P37231, Q07869, P55055, P15056, Q13133, P36888, P02768, P05089, O60285, O75469, P24941, P35398, P28907, P03372, P35968, P09211, P08253, P04818, P03950, Q07912, P42574,
GOTERM_CC_DIREC	GO:0005886~plasma membrane	41	46.07	1.75E-06	P05019, P12931, P11362, P08581, P48736, P06737, P00797, P07900, P30043, P11142, P27986, Q00987, O15530, P15153, P29474, P21802, Q9UNQ0, P22303, P16109, P06213, P00734, P15056, P10636, P36888, P05067, P18031, P28907, P00742, P03372, P35968, P33527, P09211, P08183, P08253, Q07912, P42574, P00749, P00533, P00747,
GOTERM_CC_DIREC	GO:0005737~cytoplasm	38	42.70	0.00483	P12931, P48736, P14174, P35228, P06737, Q06520, P08246, P07900, P30043, P27986, Q00987, O15530, P15153, P29474, Q06124, P28482, P21802, P16109, P08758, P15559, P55055, P15056, P10636, P05089, P02766, Q04760, O60285, P05067, P24941, P03372, Q15746, P09211, P04818, Q07912, P42574, Q16539,
GOTERM_CC_DIREC	GO:0070062~extracellular exosome	34	38.20	3.55E-07	P02753, P12931, P14780, P14174, P06737, P05164, P08246, P07900, P11142, P30043, P15153, P01009, P28482, P06213, P15559, P08758, P00734, Q04828, P02768, P00390, P05089, P02766, Q04760, P05067, P28907, Q15746, P33527, P09211, P08183, P04179, P03950, P00749,
GOTERM_CC_DIREC	GO:0005615~extracellular space	28	31.46	9.08E-11	P05019, P09917, P02753, P14174, P14780, P00797, P05164, P08246, P11142, P01009, P16109, P22303, P12643, P00734, P47989, P02768, P05089, P02766, P23141, P05067, P60568, P09211, P08253, P03950,
GOTERM_CC_DIREC	GO:0005576~extracellular region	27	30.34	2.16E-08	P05019, P02753, P11362, P08581, P14174, P14780, P00797, P08246, P07900, P21802, P01009, P22303, P12643, P00734, P02768, P02766, P05067, P39900, P60568, P00742, P35968, P08253, P03950, P00749,

GOTERM_CC_DIREC	GO:0005654~nucleoplasm	27	30.34	4.94E-04	P14174, P09874, P11142, P30043, P07900, O15530, Q00987, Q96GD4, Q96RI1, P21802, P28482, P16109, P37231, Q07869, P55055, P00374, Q13133, O60285, O75469, P24941, P35398, P03372,
GOTERM_CC_DIREC	GO:0016020~membrane	19	21.35	0.0167	P48736, P09874, P28907, P07900, P03372, P11142, P27986, O15530, P15153, P33527, P08183, P21802, Q07912, P16109, P22303, P06213, P08758, P00533, P06276
GOTERM_CC_DIREC	GO:0005739~mitochondrion	16	17.98	0.00167	P12931, P00390, P09874, P05164, P09211, P04179, P08253, Q06124, P04818, P28482, P21397, Q16678, Q16539, P31749, P45983, P15056
GOTERM_CC_DIREC	GO:0009986~cell surface	13	14.61	1.11E-05	P08581, P14174, P05067, P28907, P08246, P07900, P08183, P21802, P22303, P00749, P00533, P00747, P12643
GOTERM_CC_DIREC	GO:0043234~protein complex	10	11.24	1.67E-04	P07900, P36888, P02753, Q00987, P02768, P02766, Q06124, P28482, P09874, P31749
GOTERM_CC_DIREC	GO:0005788~endoplasmic reticulum lumen	7	7.87	3.35E-04	P00742, P36888, P01009, P23141, P06276, P00734, P08709
GOTERM_CC_DIREC	GO:0005925~focal adhesion	7	7.87	0.01164	P11142, O15530, P15153, P28482, P08758, P00749, P00533
GOTERM_CC_DIREC	GO:0031093~platelet alpha granule	5	5.62	1.43E-04	P05019, P02768, P01009, P05067, P00747
GOTERM_CC_DIREC	GO:0043235~receptor complex	5	5.62	0.00334	Q13133, P11362, P05067, P06213, P00533
GOTERM_CC_DIREC	GO:0016023~cytoplasmic, membrane-bounded vesicle	5	5.62	0.00449	P35968, O15530, P11362, P21802, P03950
GOTERM_CC_DIREC	GO:0072562~blood microparticle	5	5.62	0.00631	P11142, P02768, P06276, P00747, P00734
GOTERM_CC_DIREC	GO:0043209~myelin sheath	5	5.62	0.00631	P07900, P11142, P02768, P04179, P14174
GOTERM_CC_DIREC	GO:0005768~endosome	5	5.62	0.02357	P35968, Q07912, P05067, P24941, P00533
GOTERM_CC_DIREC	GO:0005901~caveola	4	4.49	0.00383	P12931, P29474, P28482, P06213
GOTERM_CC_DIREC	GO:0005770~late endosome	4	4.49	0.02127	P11142, P12931, P28482, P49638
GOTERM_CC_DIREC	GO:0031982~vesicl	4	4.49	0.0241	P09211, P14174, P08709, P31749
GOTERM_CC_DIREC	GO:0005641~nuclear envelope lumen	3	3.37	6.33E-04	P09917, P05067, P06276
GOTERM_CC_DIREC	GO:0097443~sorting endosome	2	2.25	0.01918	P35968, P18031

GOTERM_MF_DIREC	GO:0005515~protein binding	69	77.53	8.9E-07	P05019, P12931, P48736, P14780, P14174, P06737, P11142, P27986, O15530, Q00987, Q9UNQ0, P08758, P15559, P06213, Q07869, P00734, P55055, Q04828, P15056, O60285, O75469, P04035, P35398, P00742, P35968, P09211, Q07912, P00749, Q16539, P00747, P45983, P09917, P02753, P11362, P08581, P35228, P09874, Q06520, P08246, P07900, Q96GD4, P29474, Q96RI1, Q06124, P01009, P28482, P21802, P16109, P22303, P37231, P12643, P10636, Q13133, P36888, P02768, P02766, P49638, P24941, P05067, P18031, P03372, Q15746,
GOTERM_MF_DIREC	GO:0005524~ATP binding	26	29.21	9.8E-08	P12931, P11362, P08581, P48736, P06737, P11142, P07900, O15530, Q96GD4, P21802, Q9UNQ0, P28482, P06213, P15056, P36888, O60285, P24941, P35968, Q15746, P33527, P08183, Q07912, Q16539,
GOTERM_MF_DIREC	GO:0042802~identical protein binding	19	21.35	4.7E-08	P02768, P11362, P02766, P14780, P05067, P09874, P03372, P07900, Q00987, P04179, P01009, P28482, Q07912, P15559, P37231, P00533, [illegible]
GOTERM_MF_DIREC	GO:0008270~zinc ion binding	15	16.85	0.00279	Q13133, Q04760, P14780, O75469, P09874, P35398, P18031, P39900, P03372, Q00987, Q96RI1, P08253, [illegible]
GOTERM_MF_DIREC	GO:0019899~enzyme binding	14	15.73	1.7E-08	P10636, P12931, P05067, P09874, P18031, P03372, P11142, Q00987, P37231, Q16539, P00533, P06276, [illegible]
GOTERM_MF_DIREC	GO:0016301~kinase activity	13	14.61	4.3E-09	P27986, Q15746, O15530, P12931, P28482, P21802, P48736, Q07912, P00749, P24941, P31749, P15056, [illegible]
GOTERM_MF_DIREC	GO:0004672~protein kinase activity	12	13.48	2.6E-06	P12931, O15530, Q96GD4, P08581, P48736, O60285, Q07912, [illegible]
GOTERM_MF_DIREC	GO:0042803~protein homodimerization activity	12	13.48	0.00141	P07900, P36888, P47989, P11362, P04818, P21802, Q9UNQ0, [illegible]
GOTERM_MF_DIREC	GO:0004674~protein serine/threonine kinase activity	11	12.36	2.6E-05	O15530, Q96GD4, P28482, P48736, O60285, Q07912, Q16539, [illegible]
GOTERM_MF_DIREC	GO:0005102~receptor binding	10	11.24	9.2E-05	P12931, P03950, P14174, P05067, P35228, P00747, P12643, P00734, [illegible]
GOTERM_MF_DIREC	GO:0008144~drug binding	9	10.11	5.6E-09	P09211, P02768, P04818, O75469, P37231, P06737, P11712, Q07869, [illegible]

GOTERM_MF_DIREC	GO:0004713~protein tyrosine kinase activity	9	10.11	4.6E-07	P07900, P35968, P12931, P11362, P08581, P21802, Q07912, P06213, P00533
GOTERM_MF_DIREC	GO:0004252~serine-type endopeptidase activity	9	10.11	5.5E-05	P08246, P00742, P08253, P14780, P00749, P00747, P00734, P39900, P08709
GOTERM_MF_DIREC	GO:0004879~RNA polymerase II transcription factor activity, ligand-activated sequence-specific DNA binding	8	8.99	6.1E-10	Q13133, P03372, Q96RI1, O75469, P37231, Q07869, P35398, P55055
GOTERM_MF_DIREC	GO:0003707~steroid hormone receptor activity	8	8.99	1.6E-08	Q13133, P03372, Q96RI1, O75469, P37231, Q07869, P35398, P55055
GOTERM_MF_DIREC	GO:0043565~sequence-specific DNA binding	8	8.99	0.01846	Q13133, P03372, Q96RI1, O75469, P37231, Q07869, P35398, P55055
GOTERM_MF_DIREC	GO:0008201~heparin binding	7	7.87	0.00019	P05164, P08246, P11362, P21802, P03950, P16109, P05067
GOTERM_MF_DIREC	GO:0008134~transcription factor	7	7.87	0.00368	P27986, P03372, P28482, P37231, P09874, Q07869, P35398
GOTERM_MF_DIREC	GO:0005158~insulin receptor binding	6	6.74	5.2E-07	P27986, P05019, P12931, O15530, Q06124, P18031
GOTERM_MF_DIREC	GO:0004714~transmembrane receptor protein tyrosine kinase activity	6	6.74	1.5E-06	P36888, P35968, P08581, P21802, P06213, P00533
GOTERM_MF_DIREC	GO:0046934~phosphatidylinositol-4,5-bisphosphate 3-kinase activity	6	6.74	1.8E-05	P27986, P11362, Q06124, P21802, P48736, P00533
GOTERM_MF_DIREC	GO:0019903~protein phosphatase	6	6.74	1.9E-05	P07900, P27986, P08581, Q16539, P37231, P00533
GOTERM_MF_DIREC	GO:0020037~heme binding	6	6.74	0.00075	P05164, P12931, P29474, Q16678, P35228, P11712
GOTERM_MF_DIREC	GO:0050661~NADP binding	5	5.62	3.2E-05	P00390, P29474, P04035, P35228, P00374
GOTERM_MF_DIREC	GO:0016303~1-phosphatidylinositol-3-kinase activity	5	5.62	7.3E-05	P27986, P11362, Q06124, P21802, P48736
GOTERM_MF_DIREC	GO:0001948~glycoprotein binding	5	5.62	0.00037	P07900, P01009, P16109, P00533, P08709
GOTERM_MF_DIREC	GO:0005506~iron ion binding	5	5.62	0.0084	P09917, P47989, P29474, Q16678, P11712
GOTERM_MF_DIREC	GO:0016491~oxidoreductase activity	5	5.62	0.0206	P00390, P29474, P21397, P11712, O04828
GOTERM_MF_DIREC	GO:0005215~transporter activity	5	5.62	0.02128	P02753, P33527, P08183, Q9UNQ0, P49638
GOTERM_MF_DIREC	GO:0001077~transcriptional activator activity, RNA polymerase II core promoter proximal region sequence-specific binding	5	5.62	0.03491	Q13133, P03372, Q96RI1, Q07869, P55055

GOTERM_MF_DIREC	GO:0030235~nitric-oxide synthase regulator activity	4	4.49	7.5E-06	P07900, P03372, P00533, P31749
GOTERM_MF_DIREC	GO:0005159~insulin-like growth factor receptor binding	4	4.49	6E-05	P27986, P05019, P06213, P00797
GOTERM_MF_DIREC	GO:0019825~oxygen binding	4	4.49	0.00188	P02768, P04179, Q16678, P11712
GOTERM_MF_DIREC	GO:0050660~flavin adenine dinucleotide binding	4	4.49	0.00453	P47989, P00390, P29474, P35228
GOTERM_MF_DIREC	GO:0008233~peptidase activity	4	4.49	0.01161	P08246, P42574, P05067, P00797
GOTERM_MF_DIREC	GO:0005178~integrin binding	4	4.49	0.01752	P05019, P35968, P12931, P00533
GOTERM_MF_DIREC	GO:0005088~Ras guanyl-nucleotide exchange factor activity	4	4.49	0.02224	P60568, P11362, P21802, P00533
GOTERM_MF_DIREC	GO:0032052~bile acid binding	3	3.37	0.00074	Q96RI1, P06737, Q04828
GOTERM_MF_DIREC	GO:0004716~receptor signaling protein tyrosine kinase activity	3	3.37	0.00118	P35968, P06213, P00533
GOTERM_MF_DIREC	GO:0043560~insulin receptor substrate binding	3	3.37	0.00143	P27986, Q06124, P06213
GOTERM_MF_DIREC	GO:0004707~MAP kinase activity	3	3.37	0.00235	P28482, Q16539, P45983
GOTERM_MF_DIREC	GO:0046965~retinoid X receptor	3	3.37	0.0027	Q96RI1, P37231, P55055
GOTERM_MF_DIREC	GO:0046875~ephrin receptor binding	3	3.37	0.00805	P12931, P48736, P18031
GOTERM_MF_DIREC	GO:0051721~protein phosphatase 2A binding	3	3.37	0.00866	P04035, P18031, P31749
GOTERM_MF_DIREC	GO:0004712~protein serine/threonine/tyr	3	3.37	0.00996	Q96GD4, Q07912, P31749
GOTERM_MF_DIREC	GO:0030331~estrogen receptor binding	3	3.37	0.0159	P12931, P37231, P09874
GOTERM_MF_DIREC	GO:0001046~core promoter sequence-specific DNA binding	3	3.37	0.02113	P03372, P37231, P35398
GOTERM_MF_DIREC	GO:0042626~ATPase activity, coupled to transmembrane movement of substances	3	3.37	0.02206	P33527, P08183, Q9UNQ0
GOTERM_MF_DIREC	GO:0097110~scaffold protein binding	3	3.37	0.02595	P12931, Q00987, P29474
GOTERM_MF_DIREC	GO:0004175~endopeptidase activity	3	3.37	0.03227	P08246, P14780, P39900
GOTERM_MF_DIREC	GO:0003990~acetylcholinesterase	2	2.25	0.0104	P22303, P06276

GOTERM_MF_DIREC	GO:0004104~cholinesterase activity	2	2.25	0.01556	P22303, P06276
GOTERM_MF_DIREC	GO:0004517~nitric-oxide synthase activity	2	2.25	0.01556	P29474, P35228
GOTERM_MF_DIREC	GO:0070851~growth factor receptor binding	2	2.25	0.02069	P12931, P05067
GOTERM_MF_DIREC	GO:0043208~glycosphingolipid	2	2.25	0.02069	P60568, P16109
GOTERM_MF_DIREC	GO:0051425~PTB domain binding	2	2.25	0.02069	P05067, P06213
GOTERM_MF_DIREC	GO:0034617~tetrahydrobiopterin	2	2.25	0.02069	P29474, P35228
GOTERM_MF_DIREC	GO:0008559~xenobiotic-transporting ATPase activity	2	2.25	0.0258	P08183, Q9UNQ0
GOTERM_MF_DIREC	GO:0051525~NFAT protein binding	2	2.25	0.0258	Q16539, Q07869
GOTERM_MF_DIREC	GO:0005007~fibroblast growth factor-activated receptor activity	2	2.25	0.0258	P11362, P21802
GOTERM_MF_DIREC	GO:0043559~insulin binding	2	2.25	0.0258	P27986, P06213
GOTERM_MF_DIREC	GO:0004784~superoxide dismutase activity	2	2.25	0.0258	P04179, P15559
GOTERM_MF_DIREC	GO:0034618~arginine binding	2	2.25	0.03593	P29474, P35228
GOTERM_MF_DIREC	GO:0005021~vascular endothelial growth factor-activated receptor	2	2.25	0.03593	P36888, P35968
GOTERM_MF_DIREC	GO:0004887~thyroid hormone receptor activity	2	2.25	0.04096	Q96RI1, O75469
